# Synthesis, Spectral Characterization of Several Novel Pyrene-Derived Aminophosphonates and Their Ecotoxicological Evaluation Using *Heterocypris incongruens* and *Vibrio fisheri* Tests

**DOI:** 10.3390/molecules21070936

**Published:** 2016-07-19

**Authors:** Jarosław Lewkowski, Maria Rodriguez Moya, Marta Chmielak, Diana Rogacz, Kamila Lewicka, Piotr Rychter

**Affiliations:** 1 Department of Organic Chemistry, Faculty of Chemistry, University of Łódź, Tamka 12, 91-403 Łódź, Poland; MRM_chem@wp.pl (M.R.M.); martachmielak1@gmail.com (M.C.); 2 Institute of Chemistry, Environmental Protection and Biotechnology, Jan Długosz University in Częstochowa, 13/15 Armii Krajowej Av., 42-200 Częstochowa, Poland; diana.rogacz@gmail.com (D.R.); lewickakamilla@gmail.com (K.L.)

**Keywords:** pyrene-1-carboxaldehyde, aminophosphonates, ecotoxicity, ostracode test, *Vibrio fisheri* test

## Abstract

Four diphenyl pyrene-derived aminophosphonates were synthesized. Attempts were made to synthesize diphenyl *N*-(*R*)-α-methylbenzylamino(pyren-1-yl)methylphosphonate (**3e**) in order to obtain the chiral aminophosphonate bearing a pyrene moiety. Because these attempts failed, dimethyl and dibenzyl *N*-(*R*)-α-methylbenzyl substituted aminophosphonates **4** and **5** were synthesized and the predominant diastereoisomer of dimethyl aminophosphonate **4** was isolated. The resolution of the diastereomeric mixture of **5** failed. Aminophosphonates **3a**–**d** and the predominant diastereoisomer of **4** were investigated in terms of their ecotoxicity using tests performed on the ostracode *Heterocypris incongruens* and the fluorescent bacterium *Vibrio fisheri*. The tests confirmed the moderate-to-high ecotoxicity of aminophosphonates **3a**–**d** and **4**, but no evident correlation between the structure and toxicity has been found.

## 1. Introduction

Aminophosphonic derivatives are well known for their biological activity. They are described as effective inhibitors of various enzymes and, therefore they find applications as crop protective [1], antibacterial [2] or anticancer [3] agents. Analogues of *C*-phenylphosphonoglycine, have been demonstrated to show activity in various fields as herbicides and plant growth regulators [4], agrochemical fungicides [5,6] and glutamate receptor agonists and antagonists [7].

In a sort of logical consequence, a lot of compounds from this group have been synthesized, including those bearing benzene and its polycyclic aromatic analogues [8], five- and six-membered heteroaromatic rings [9,10], and even the ferrocene [11] moiety. Their syntheses are generally based on the Kabachnik-Fields and the aza-Pudovik reactions [12,13,14].

In contrast, aminophosphonic derivatives of 1-pyrene, i.e., amino(1-pyrene)methylphosphonic compounds have been described only twice in the chemical literature. Hudson et al. [15] reported the synthesis of diethyl *N*‑benzhydrylamino(1-pyrene)methyl phosphonate, and Jayaprakash and co-workers [16] described the synthesis of a series of *N*‑aryl substituted, diethyl amino(1-pyrene)methyl phosphonates and results of preliminary studies on their fluorescence properties.

We have tried to fill this void and very recently have published results of our studies on the synthesis, photophysical properties and cytotoxic action of a series of α-aminophosphonates bearing pyren-1-yl moieties [17]. They demonstrated moderate-to-satisfactory cytotoxicities against two colon cancer cell lines. Such interesting biological properties may result in potential future applications and their biological hazards should be evaluated using typical biotoxicological tests.

Herein, we wish to report the synthesis of a new series of *N*-substituted-*C*-(pyren-1-yl)-phosphonoglycine derivatives and their ecotoxicological properties evaluated using ostracode (*Heterocypris incongruens*) and fluorescence bacteria *Vibrio fisheri* tests.

Nowadays, ecotoxicology faces the challenge of evaluating and predicting the effects of a variety of chemicals on aquatic species and ecosystems. Since a chemical pollutant may follow various routes from a source to the target, there is an urgent need to follow their potential ecotoxicological impact using different biotests [18,19,20,21].

Toxkit microbiotests are presently used in many countries worldwide for biomonitoring of contaminated waters and soils. These small-scale bioassays are becoming more and more popular in laboratories all over the world because of their various useful advantages, such as high degree of standardization and precision, simple testing procedures which are very clearly documented down to the last detail, operation with small sample volumes, the repeatability as well as giving comparable results to equivalent ISO and OECD full-scale tests. However, the major advantage of Toxkit microbiotests in comparison to conventional bioassays is that the tested microorganisms are incorporated in the kits in a dormant or immobilized form, from which they can be hatched or activated “on demand” prior to carrying out the toxicity tests [22].

The Microtox^®^ system test with the luminescent bacterium *Vibrio fischeri* and the crustacean *Heterocypris incongruens*, when used as bioindicator allows one to monitor potential toxicity of soil or water contaminants in a rapid, simple and inexpensive way. This bioanalytical technique allows one to overcome some disadvantages of the traditional physicochemical methods such as concentration of toxic substances below analytical limits or potential chemical interactions leading to additive, synergistic or antagonistic effect which cannot be identified using the abovementioned methods. The *Vibrio fischeri* bioassay can be applied in all types of water, including surface and ground water, wastewater, drilling sump fluids and many other aqueous solutions [23]. Both *Vibrio fischeri* and *Heterocypris incongruens* bioassay tests have been already successfully used for ecotoxicity determination of various pharmaceuticals, pesticides, ionic liquids, detergents, heavy metals, road dust, eluates and organic extracts of wastes, etc. [24,25,26,27,28,29,30].

As it was mentioned above, the aim of the study was the preliminary evaluation of the potential toxicity of newly synthesized aminophosphonates **3a**–**d** and **4**, if introduced to the environment. In order to perform it, the Microtox^®^ test using the bioluminescent bacterium *Vibrio fischeri* was employed to examine the acute toxicity of sediment eluates loaded with tested compounds. Due to the cost effective, simple operation and time-savings, the biotest based on the bioluminescence inhibition of *Vibrio fischeri* is probably the most widely applied bacterial test used for environmental pollution monitoring.

However, due to fact that the obtained substances are almost insoluble in water, the Microtox^®^ solid phase test (MSPT), as a one of the most popular tests for sediment toxicity assessments due to its simplicity, reproducibility, ecological relevance and sensitivity, has been used [26,31,32,33].

Additionally, the standardized crustacean biotest with *Heterocypris incongruens* was selected as a representative benthic invertebrate species for screening the toxicity of sediments containing the tested substances. Crustaceans are promising representative species of the benthic environment as they spend their lives swimming in water and in direct contact with the sediment. Therefore, the Ostracodtoxkit^®^ test has been used for toxicity evaluation. The toxicity test using mentioned freshwater ostracodes is a sub-chronic static test that exposes individuals to whole sediments over a period of 6 days, the endpoints being mortality and growth changes [34].

## 2. Results and Discussion

### 2.1. Synthesis of Aminophosphonic Derivatives ***3a***–***e***, ***4*** and ***5*** Bearing 1-Pyrene Moieties 

Schiff bases **1a**–**e** were synthesized following the published and commonly known procedure by simple mixing pyrene-1-carboxaldehyde with an appropriate amine in hexane (in a case of *p*-anisidine, *p*-toluidine and aniline) or methanol (for benzylamine and (*R*)-α-methylbenzylamine) and stirring them at room temperature for 24 h; the reaction with *p*-anisidine required 48 h [14]. This procedure gave imines **1a**–**e**, which were isolated and used for further conversions without any purification. ^1^H-NMR spectra were recorded only to verify the identity, based on the diagnostic ^1^H-NMR singlet corresponding to the proton of the azomethine group (–CH=N–) above 8 ppm [17].

**Scheme 1:**
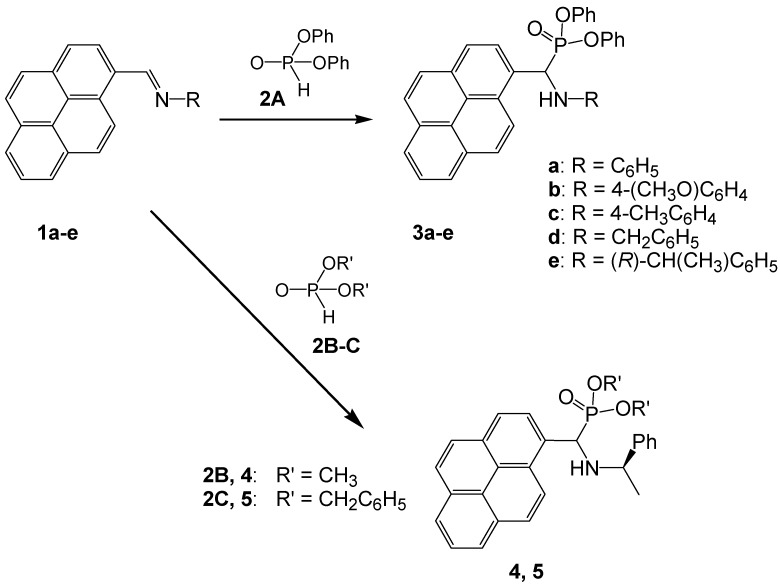
Synthesis of *N*-substituted amino(1-pyrene)methylphosphonates **3a**–**e**, **4** and **5**.

Aminophosphonates **3a**–**d**, **4** and **5** were synthesized basing on the aza-Pudovik reaction, i.e., the addition of diphenyl phosphite to imines **1a**–**d**. However, important modifications were necessary to be introduced, whereby reactions were carried out in a small amount of dichloromethane and diphenyl phosphite was used in three-fold excess. The reaction mixtures were heated for 5 min then stirred for 24 h at room temperature. After workup as described in the Experimental Section, the resulting aminophosphonates **3a**–**d** were obtained in 60%–70% yields (Scheme 1). They were purified by column chromatography on neutral aluminum oxide, because diphenyl amino(1-pyrene)methylphosphonates **3a**–**d** decomposed on silica gel.

The identity of synthesized compounds was verified by means of ^1^H-, ^31^P- and ^13^C-NMR as well as by FT-IR spectroscopy. The proton NMR spectra of diphenyl pyrene-derived aminophosphonates **3a**–**d** showed the characteristic signals of all compounds, i.e., the doublet corresponding to the proton from the CH-P bond and the system of signals attributable to the pyrene ring protons. The ^13^C-NMR spectra revealed the presence of two doublets at around 150 ppm with a coupling constant close to 10 Hz, which are attributed to the phenyl ring carbons from the P-O-C groups. Doublets in the 50–55 ppm range with coupling constants between 150 and 160 Hz are easily recognizable as C-P bond signals. IR bands at around 1250 cm^−1^ correspond to the bending vibrations of P=O bonds, while the presence of aromatic carbon-carbon bonds was confirmed by characteristic bands at around 1600, 1570, 1500 and 1450 cm^−1^. Scans of NMR spectra are collected in the Supplementary Materials as Figures S1–S7.

In order to obtain the chiral, non-racemic diphenyl ester of a phosphonoglycine derivative bearing a pyren-1-yl moiety, the synthesis of diphenyl *N*-(*R*)-α-methylbenzylamino(pyren-1-yl)-methylphosphonate (**3e**) was performed using the method described above. The resulting mixture contained two diastereomers in a 100:15 ratio, but all attempts to purify the product by column chromatography on neutral aluminum oxide caused extensive decomposition of the product. From among available adsorbents, silica gel was totally inconvenient (*vide supra*), and chromatography on microcrystalline cellulose with chloroform allowed us to get rid of impurities only roughly, i.e., crude diphenyl *N*-(*R*)-α-methylbenzylamino(pyren-1-yl)methylphosphonate (**3e**) was obtained.

Thus, in order to investigate the stereochemistry of phosphite addition to the azomethine bond of pyrene-1-carboxaldimine **1e**, addition of dimethyl and dibenzylphosphites has been performed. Dimethyl *N*-(*R*)-α-methylbenzylamino(pyren-1-yl)methylphosphonate (**4**) was obtained as a mixture of two diastereoisomers in a 100:13 ratio. We succeeded in separation of the predominant diastereoisomer using column chromatography on silica gel. Its absolute configuration however could not be determined, because the predominant isomer of aminophosphonate **4** is a dense oily liquid, thus an X-ray study could not be performed. Isolation of the minor diastereoisomer of **4** failed, it was always obtained in a mixture with the major one.

Dibenzyl *N*-(*R*)-α-methylbenzylamino(pyren-1-yl)methylphosphonate (**5**) was obtained as a mixture of two diastereoisomers in a 100:26 ratio. Unfortunately, the obtained mixture of diastereoisomers was not separable by column chromatography, as all applied solvent systems led to mixtures with different ratios of isomers. However, the crude diastereomeric mixture of **5** was purified by column chromatography on silica gel to give satisfactory elemental analysis results.

Thus the obtained diphenyl aminophosphonates **3a**–**d** and the isolated predominant diastereoisomer of **4** were tested in terms of their ecotoxicity using two popular ecotoxicological tests, the ostracode *Heterocypris incongruens* and fluorescence bacteria *Vibrio fisheri*.

### 2.2. Ecotoxicological Properties of Aminophosphonates ***3a**–**d*** and ***4***

#### 2.2.1. Microtox Toxicity Assay

**Figure 1:**
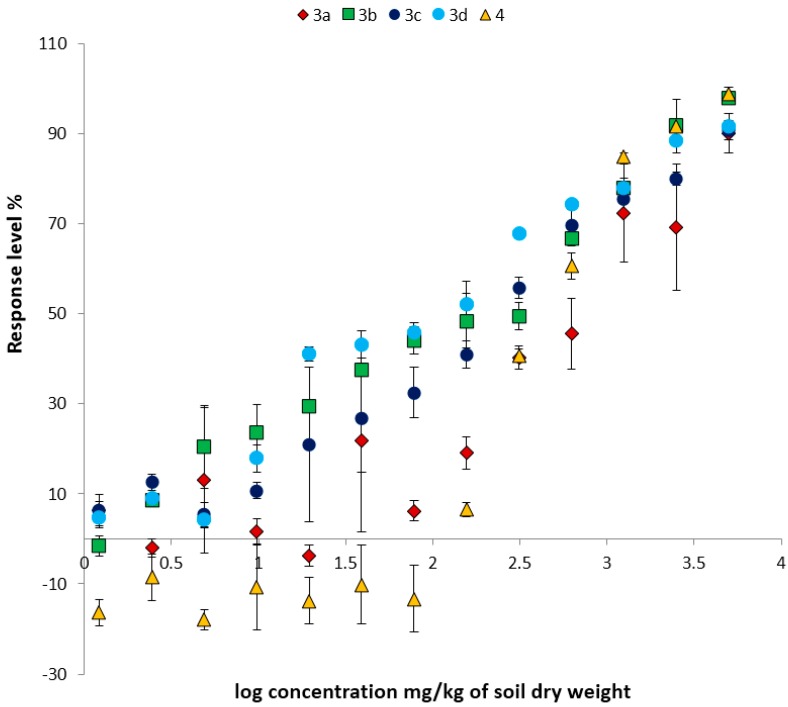
The EC_50_ graded dose-response curves for the five tested compounds **3a**–**d** and **4**. Overlapped curves are plotted by the Microtox^®^ Analyzer 500 software. Error bars represent standard deviation error (SD, *n* = 3 determinations).

Bioassays are the complementary tool for characterizing the biological effects and hazards of contaminated sediments or soils. During the solid phase Microtox^®^ assay the bioluminescent bacteria are exposed to a sediment suspension and their response reflects totally the action of toxic agents along with synergists and antagonists present in a given sample. The effects on light emission are evaluated in the liquid phase that remains after removal of the sediments by filtration. Effective concentration (EC_50_) values (Table 1) were calculated from the concentration–response curves (Figure 1).

Obtained EC_50_ values ranged between 92 and 533 mg/kg of dry weight of soil, depending on the tested compound. Among the compounds **3a**–**c** with aromatic substituents on the amine groups, diphenyl *N*-(4-methoxyphenyl)amino(pyren-1-yl)methylphosphonate (**3b**) was found to be the most toxic, with the lowest EC_50_ value (92.63 mg/kg). Much less harmful action against *Vibrio fischeri* was found for the *N*-(4-methylphenyl) derivative **3c** (EC_50_ = 214.5 mg/kg), while diphenyl *N*-phenylamino(pyren-1-yl)methylphosphonate (**3a**) demonstrated nearly a lack of toxicity. Thus, the presence of a substituent at the phenyl group linked to nitrogen seems to be crucial in terms of toxicological properties of the studied compounds **3a**–**c**. The results also showed that a methoxy substituent increases the toxicity of the compound **3b** in comparison with the methyl substituted aminophosphonate **3c**.

Among the aminophosphonates **3d** and **4** having the amino groups substituted with non-aromatic groups, results were indeed surprising. Diphenyl *N*-benzylamino(pyren-1-yl)methylphosphonate (**3d**) with a *N*-benzyl substituent demonstrated five times higher toxicity (EC_50_ = 102.7 mg/kg) as compared to the *N*-(*R*)-α-methylbenzylamino derivative **4** (EC_50_ = 533.1 mg/kg). Therefore we can conclude that the presence of a methyl substituent in the benzyl moiety dramatically decreases the toxicity of compound **4**.

#### 2.2.2. Ostracod Test Kit

Ostracodtoxkit F^TM^ is the very first sediment-contact microbiotest with a crustacean test species for the assessment of the total toxicity of sediments, hence covering the toxic hazards of both dissolved and not dissolved pollutants. Considering the very low solubility of synthesized aminophosphonates **3a**–**d** and **4**, it seemed justified to evaluate reliably their potential toxicity.

**Figure 2:**
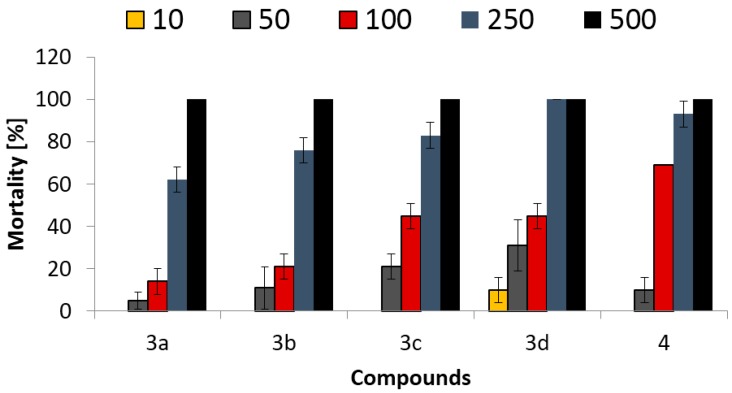
The mortality (%) of *Heterocypris incongruens* living in soil contaminated with pyrene-derived aminophosphonates **3a**–**d** and **4** at concentrations: 10, 50, 100 and 500 mg/kg of soil dry weight. Error bars represent standard deviation error (SD, *n* = 6 determinations).

The first criterion of the effect on tested crustaceans is the percent of mortality. Toxicity was expressed in % of mortality, i.e., the lethal effect of the sample on the number of ostracods compared with the control plate. As it was expected, the mortality of ostracodes increased with growing concentration of tested substance in soil (Figure 2). The concentration 500 mg/kg of soil was found to be lethal for all samples. However, among all tested compounds, *N*-benzylamino(pyren-1-yl)methylphosphonate (**3d**) demonstrated the highest toxicity at every concentration when compared to other samples (with the sole exception of aminophosphonate **4** at a concentration of 100 mg/kg of soil). Even at a concentration of 10 mg/kg of dry weight of soil, compound **3d** caused 10% mortality of crustaceans, while other samples at this concentration did not show any mortal effect.

**Figure 3:**
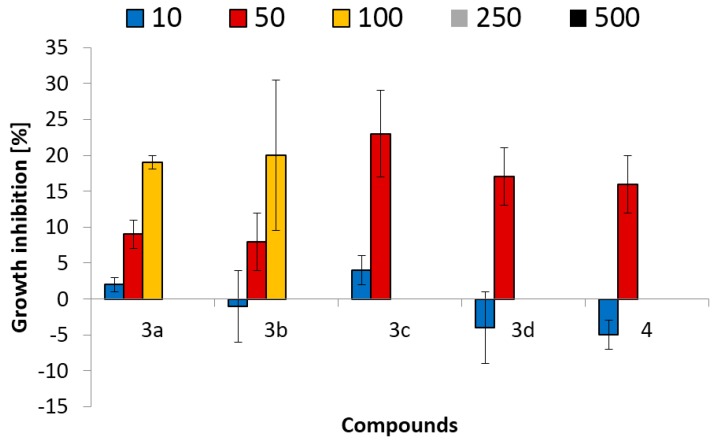
Growth inhibition (%) of *Heterocypris incongruens* living in soil contaminated with pyrene-derived aminophosphonates **3a**–**d** and **4** at concentrations of 10, 50, 100 and 500 mg/kg of soil dry weight. Error bars represent standard deviation error (SD, *n* = 6 determinations).

Growth inhibition is the second criterion of the toxic effect indicated by the Ostracodtoxkit F™ microbiotest. This criterion allows one to evaluate the sub-lethal toxicity of sediments. The growth inhibition is determined by comparing the size of surviving ostracods living in the test sediment with the size of ostracods living in the reference sediment at the end of the test. Determination of the sub-lethal impact of sediment toxicants is justified only for sediments which do not cause a high ostracod mortality. It is advised that the growth inhibition should only be determined for sediments which mortality was found to be less than 30%. Therefore, the growth inhibition was chosen as a criterion of sub-lethal effects to determine toxicity not inducing substantial mortality in the test organisms. Consequently, measurements of lengths were only performed when the mortality was less than 30%. In this respect, the growth inhibition rates have not been measured for aminophosphonates **3c**, **3d** and **4** at concentrations of 100, 250 and 500 mg per kg of soil dry weight. Crustaceans were more resistant against compounds **3a** and **3b** and their growth inhibition reached ca. 20% for a concentration of 100 mg/kg of soil, nevertheless it was not measured at 250 and 500 mg per kg of soil dry weight. Values of growth inhibition for compounds **3c**, **3d** and **4** at 50 mg/kg of soil concentration varied between 16% and 23% (Figure 3).

Comparison of results of luminescence inhibition for *Vibrio fischeri* tests with growth inhibition of crustaceans for all concentration of compounds did not show any reasonable relationship. Only diphenyl *N*-benzylamino(pyren-1-yl)methylphosphonate (**3d**), which was strongly toxic for *Vibrio fischeri* (EC_50_ ~ 103 mg/kg of soil) also revealed the highest mortality of ostracods even at a concentration of 10 mg/kg of soil.

However, diphenyl *N*-(4-methoxyphenyl)amino(pyren-1-yl) methylphosphonate (**3b**) that induced a very strong luminescence inhibition in *V. fischeri* was only moderately toxic for crustaceans considering both the mortality and the growth inhibition index. On the other hand, diphenyl *N*-phenylamino(pyren-1-yl)methylphosphonate (**3a**), being nearly non-toxic for *V. fischeri* (EC_50_ close to 500 mg/kg of soil) is also very weakly toxic for ostracods (with respect to both criteria, the mortality and the growth inhibition).

Thus, based on the obtained results, *Vibrio fischeri* bacteria were very sensitive to aminophosphonates **3b** and **3d**, since relatively low effective concentration (EC_50_) values were determined, while for ostracods, the highest negative impact considering both mortality and inhibition growth have been found for diphenyl *N*-benzylamino(pyren-1-yl)methylphosphonate (**3d**). Therefore, being the most toxic against both tested organisms, the aminophosphonate **3d** should be considered as a potentially hazardous compound for environment, which may be toxic against many other living species. Its high toxicity may be related with its *N*-benzyl group, which is more flexible than other substituents and may bind more easily to enzymes or receptors of the tested organisms. Nevertheless, this is only a very preliminary hypothesis, which requires further verification.

The usefulness of bioassays for the assessment of areas affected by phosphate industry wastes has already been reported and discussed [35,36]. Authors performing risk assessments in such contaminated areas have proven that the development of sensitive and observable organisms as bio-indicators for monitoring and management of the environmental pollution is a very useful tool to complement chemical analyses. Biological analysis allows one to reflect the harmful effects of contaminants on organisms and provides data to reveal the mechanisms of the toxic effects including formation, development and removal of contaminants.

Since the soil is generally the reservoir for agrochemical residues, the evaluation of any xenobiotics including pesticides using a simple battery of assay tests is strongly needed to control level and fate of their residues. In a successful study focused on the ecotoxicological evaluation of select pesticides (chlorpyrifos, glyphosate, vinclozolin, endosulfan) present in agricultural soils, Antunes et al have reported the use of standard aquatic bioassays for testing of soil eluates [30].

Our obtained effective concentration values are very close to the inhibition concentration (IC_50_) values for herbicides examined by Joly et al. [28]. Toxicity values of the pesticides *S*-metolachlor, nicosulfuron and benoxacor were 178.4, 167.8 and 93.3 mg/L, respectively. In this respect, the toxicity of the first two herbicides are close to that of the tested aminophosphonate **3c**, while the last IC_50_ value matches the values of compounds **3b** and **3d**. Nevertheless, the action of aminophosphonates **3a**–**d** and **4** against *V. fischeri* demonstrated much lower toxicity when compared to the herbicides tested by the mentioned authors.

Ostracodtoxit tests using *Heterocypris incongruens* are also a very useful tool for environmental toxicity determination. Results related to mortality and growth inhibition of ostracods correlated with various types of polycyclic aromatic hydrocarbons (PAHs) have been reported by Cvancarova et al. [37]. They found that *H. incongruens* was strongly sensitive to bioavailable PAHs, while luminescence inhibition of *V. fischeri* varied widely between ca. 15%–90% depending on the type of soil examined.

Oleszczuk and Hollert [38], in their studies devoted to determination of the influence of different soils on sewage sludge toxicity, have found that the mortality values of *Heterocypris incongruens* ranged from 0.26% to 11.5% depending on the sludge tested [39]. Growth inhibition values ranged from 10.7% to 36.2% depending on the type of sludge. Płaza et al. [39] applied Microtox^®^, Ostracodtoxkit F^™^, to assay bioremediation processes in soils heavily contaminated with petroleum. The test species demonstrated varying sensitivity to soils and the effects on test organisms exposed to tested soils correlated with the soil contaminants’ concentrations [39].

Results of toxicity against *H. incongruens* obtained for the studied aminophosphonates **3a**–**d** and **4** were at a similar level. The mortality varied from ca. 5% until 70% for the concentration range 10–100 mg per kg of soil. At higher concentrations, the tested compounds were practically lethal for crustaceans. The growth inhibition varied from 16% up to 23% for a concentration of 50 mg per kg of soil. All the data presented above lead us to stress the necessity of handling pyrene-derived aminophosphonic derivatives with special care, as they may be potentially hazardous for the environment.

## 3. Materials and Methods

### 3.1. Chemistry

All solvents (POCh, Gliwice, Poland) were applied routinely and dried prior to use. Commercial reagents (Aldrich, Poznań, Poland) were generally used as received. NMR spectra were recorded on an Avance III 600 MHz apparatus (Bruker, Billerica, MA, USA) operating at 600 MHz (^1^H-NMR), 150 MHz (^13^C-NMR) and 243 MHz (^31^P-NMR). IR spectra were recorded on a Nexus FT-IR spectrometer (Thermo Nicolet, Waltham, MA, USA). Rotations were measured using a 241 MC polarimeter (Perkin-Elmer, Waltham, MA, USA). Melting points were measured using a MelTemp II apparatus (Bibby Scientific Limited, Staffordshire, UK), in a capillary and were not corrected. Elemental analyses were performed in the Laboratory of Microanalysis, the Center of Molecular and Macromolecular Studies PAS in Łódź and the Laboratory of Molecular Spectroscopy at the Faculty of Chemistry, University of Łódź.

#### 3.1.1. General Procedure for Synthesis of Phosphonates **3a**–**d**, **4** and **5**

Equimolar quantities of 1-pyrenecarboxaldehyde and amine (2 mmol each) were dissolved in hexane (**1a**–**c**) or methanol (**1d**–**e**) (20 mL) and stirred at reflux for 24 h (**1b**–**c**, **1d**–**e**) or 48 h (**1a**). The reaction was monitored by ^1^H-NMR and after the completion, the solvent was removed under reduced pressure, and obtained products were used directly for further conversion. Imine (1 mmol) and the appropriate phosphite **2A**–**C** (3 mmol or 5–6 mmol in a case of dimethyl phosphite due to the low solubility of the imine in it) were placed in a 25 mL round-bottom flask, which was heated with stirring on a water bath for 5 min until all of the imine was dissolved in the phosphite. In the case of derivatives **3a**–**d**, dichloromethane (2 mL) was added in order to improve the solubility. Then the mixture was stirred at room temperature for 24 h. The crude aminophosphonates were purified by column chromatography on neutral aluminum oxide using chloroform as eluent.

*Diphenyl N-phenylamino(pyren-1-yl)methylphosphonate* (**3a**). Y = 0.41 g (76%), yellow solid, m.p. 184–185 °C. ^1^H-NMR (CDCl_3_): δ 8.38 (d, ^3^*J*^HH^ = 9.3 Hz, H_pyr_, 1H); 8.28 (dd, ^3^*J*^HH^ = 8.0 and ^4^*J*_HH_ = 2.6 Hz, H_pyr_, 1H); 8.20 (t, H_pyr_, ^3^*J*^HH^ = 8.0 Hz, 2H); 8.12 (d, ^3^*J*^HH^ = 8.0 Hz, H_pyr_, 1H); 8.10 (d, ^3^*J*^HH^ = 9.2 Hz, H_pyr_, 1H); 8.07–8.06 (m, H_pyr_, 1H); 8.04–8.01 (m, H_pyr_, 2H); 7.30–7.29 (m, H_Ph_, 5H); 6.94 (t, ^3^*J*^HH^ = 7.4 Hz, H_Ph_, 1H); 6.84 (dd, ^3^*J*^HH(1)^ = ^3^*J*^HH(2)^ = 7.8 Hz, H_Ph_, 2H); 6.79 (app. d, ^3^*J*^HH^ = 8.5 Hz, *p*-C_6_H_4_, 2H); 6.67 (d, ^3^*J*^HH^ = 7.4 Hz, H_Ph_, 2H); 6.46 (app. d, ^3^*J*^HH^ = 8.5 Hz, *p*-C_6_H_4_, 2H); 5.92 (d, ^2^*J*_PH_ = 24.1 Hz, CHP, 1H); 5.10–5.08 (m, POCH_2_, 2H); 4.65 and 4.22 (Part of AMX system, ^2^*J*_HH_ = 11.6 Hz and ^3^*J*_PH_ = 7.8 and 8.5 Hz, POCH_2_, 2H). ^13^C-NMR (CDCl_3_): δ 150.7 (d, ^2^*J*_CP_ = 9.9 Hz, C_PO*C*_); 150.3 (d, ^2^*J*_CP_ = 9.5 Hz, C_PO*C*_); 146.1 (d, ^3^*J*_CP_ = 15.27 Hz, C_Ph_); 131.6 (C_pyr_); 131.5 (d, ^4^*J*_CP_ = 3.1 Hz, C_pyr_); 130.8 (C_pyr_); 130.2 (C_pyr_) 130.0 (C_Ph_); 129.6 (C_pyr_); 129.5 (d, ^4^*J*_CP_ = 2.0 Hz, C_Ph_); 128.7 (C_pyr_); 128.6 (d, ^4^*J*_CP_ = 2.1 Hz, C_pyr_); 127.9 (C_pyr_); 127.7 (C_pyr_); 127.4 (C_pyr_); 127.0 (C_pyr_); 126.8 (C_pyr_); 126.3 (C_pyr_); 125.8 (C_Ph_); 125.7 (C_Ph_); 125.6 (d, ^4^*J*_CP_ = 3.4 Hz, C_pyr_); 125.5 (C_pyr_); 125.2 (C_pyr_); 125.0 (C_pyr_); 122.1 (C_pyr_); 120.9 (d, ^3^*J*_CP_ = 4.0 Hz, C_Ph_); 119.9 (d, ^3^*J*_CP_ = 4.4 Hz, C_Ph_); 119.1 (C_Ph_); 114.2 (C_Ph_); 52.2 (d, ^1^*J*_CP_ = 154.6 Hz, P*C*). ^31^P-NMR (CDCl_3_): δ 16.16. IR (KBr): 3313 (νCH); 3037; 2923 (CH_3_); 1600, 1581; 1524, 1483 (C=C); 1249 (P=O); 1214; 1185; 1062; 1021 (P-O); 941; 853; 824; 622.Elemental analysis: Calcd. for C_35_H_26_NO_3_P: C, 77.91; H, 4.86; N, 2.60. Found: C, 77.76, H, 4.86, N, 2.79.

*Diphenyl N-(4-methoxyphenyl)amino(pyren-1-yl)methylphosphonate* (**3b**). Y = 0.3 g (52%), yellow solid, m.p. 161 °C. ^1^H-NMR (CDCl_3_): δ 8.49 (d, ^3^*J*^HH^ = 9.2 Hz, H_pyr_, 1H); 8.34 (dd, ^3^*J*^HH^ = 7.7 and ^4^*J*_HH_ = 1.9 Hz, H_pyr_, 1H); 8.24-8.21 (m, H_pyr_, 3H); 8.14 (d, H_pyr_, ^3^*J*^HH^ = 8.1 Hz, 1H); 8.08 (d, ^3^*J*^HH^ = 8.9 Hz, H_pyr_, 1H); 8.04 (d, ^3^*J*^HH^ = 7.7 Hz, H_pyr_, 1H); 8.02–8.01 (m, H_pyr_, 2H); 7.31–7.28 (m, H_Ph_, 2H); 7.19–7.16 (m, H_Ph_, 3H); 6.95–6.92 (m, H_Ph_, 2H); 6.89–6.87 (m, H_Ph_, 1H); 6.62–6.58 (m, H_Ph_, *p*-C_6_H_4_, 6H); 6.23 (d, ^2^*J*_PH_ = 24.4 Hz, CHP, 1H); 3.61 (s, CH_3_, 3H). ^13^C-NMR (CDCl_3_): δ 153.2 (d, ^2^*J*_CP_ = 5.4 Hz, C_para_); 150.7 (d, ^2^*J*_CP_ = 9.9 Hz, C_PO*C*_); 150.3 (d, ^2^*J*_CP_ = 9.7 Hz, C_PO*C*_); 131.5 (C_pyr_); 131.2 (d, ^4^*J*_CP_ = 3.2 Hz, C_pyr_); 130.8 (C_pyr_); 130.0 (C_Ph_); 129.7 (C_pyr_); 129.6 (d, ^3^*J*_CP_ = 7.2 Hz, C_pyr_); 129.5 (C_Ph_); 128.8 (C_pyr_); 128.6 (C_pyr_); 127.9 (C_pyr_); 127.7 (C_pyr_); 126.3 (C_Ph_); 125.8 (C_Ph_); 125.6 (C_pyr_); 125.6 (C_pyr_); 125.5 (C_pyr_); 125.2 (d, ^4^*J*_CP_ = 1.8 Hz, C_pyr_); 125.0 (C_pyr_); 124.9 (C_pyr_); 122.2 (C_pyr_); 121.0 (d, ^3^*J*_CP_ = 4.2 Hz, C_Ph_); 120.0 (d, ^4^*J*_CP_ = 4.4 Hz, C_Ph_); 115.7 (d, ^4^*J*_CP_ = 9.7 Hz, C_Ph_); 115.1 (C_para_); 55.7 (C_Ar_-O*C*); 53.0 (d, ^1^*J*_CP_ = 154.3 Hz, P*C*). ^31^P-NMR (CDCl_3_): δ 16.32. IR (KBr): 3383 (νNH); 3294 (νCH); 3037; 2958 (CH_3_); 1591, 1511, 1486; 1451 (C=C); 1245 (P=O); 1211; 1068; 1024 (P-O); 941; 834; 815; 618. Elemental analysis: Calcd. for C_36_H_28_NO_4_P: C, 75.91; H, 4.95; N, 2.46. Found: C, 75.76, H, 5.06, N, 2.54.

*Diphenyl N-(4-methylphenyl)amino(pyren-1-yl)methylphosphonate* (**3c**). Y = 0.44 g (79%), yellow solid, m.p. 181–182 °C. ^1^H-NMR (CDCl_3_): δ 8.51 (d, ^3^*J*^HH^ = 9.3 Hz, H_pyr_, 1H); 8.34 (dd, ^3^*J*^HH^ = 8.2 and ^4^*J*_HH_ = 2.5 Hz, H_pyr_, 1H); 8.25-8.21 (m, H_pyr_, 3H); 8.13 (d, H_pyr_, ^3^*J*^HH^ = 8.2 Hz, 1H); 8.08 (d, ^3^*J*^HH^ = 8.9 Hz, H_pyr_, 1H); 8.04 (t, ^3^*J*^HH^ = 7.6 Hz, H_pyr_, 1H); 8.01 (d, ^3^*J*^HH^ = 8.8 Hz, H_pyr_, 2H); 7.31–7.29 (m, H_Ph_, 2H); 7.20–7.15 (m, H_Ph_, 3H); 7.05–7.02 (m, H_Ph_, 2H); 6.94–6.91 (m, H_Ph_, 2H); 6.88-6.86 (m, H_Ph_, 1H); 6.68-6.64 (m, H_Ph_, 3H); 6.58–6.57 (m, H_Ph_, 2H); 6.31 (d, ^2^*J*_PH_ = 24.6 Hz, CHP, 1H). ^13^C-NMR (CDCl_3_): δ 150.7 (d, ^2^*J*_CP_ = 9.8 Hz, C_PO*C*_); 150.3 (d, ^2^*J*_CP_ = 9.7 Hz, C_PO*C*_); 143.7 (d, ^2^*J*_CP_ = 15.9 Hz, C_para_); 131.6 (C_pyr_); 131.2 (d, ^4^*J*_CP_ = 3.2 Hz, C_pyr_); 130.8 (C_pyr_); 130.0 (C_Ph_); 129.8 (C_pyr_); 129.6 (d, ^3^*J*_CP_ = 7.3 Hz, C_pyr_); 129.5 (C_para_); 128.8 (d, ^4^*J*_CP_ = 2.1 Hz, C_pyr_); 128.6 (C_pyr_); 128.3 (C_pyr_); 127.8 (C_para_); 127.7 (d, ^4^*J*_CP_ = 1.6 Hz, C_pyr_); 126.3 (C_pyr_); 125.8 (C_pyr_); 125.6 (C_Ph_); 125.5 (d, ^4^*J*_CP_ = 2.3 Hz, C_pyr_); 125.5 (C_Ph_); 125.2 (d, ^4^*J*_CP_ = 1.9 Hz, C_pyr_); 125.0 (C_pyr_); 124.9 (C_pyr_); 122.2 (C_pyr_); 121.0 (d, ^3^*J*_CP_ = 4.0 Hz, C_Ph_); 120.0 (d, ^3^*J*_CP_ = 4.4 Hz, C_Ph_); 114.3 (C_para_); 52.5 (d, ^1^*J*_CP_ = 155.1 Hz, P*C*); 20.5 (C_Ar_-O*C*). ^31^P-NMR (CDCl_3_): δ 15.23. IR (KBr): 3303 (νCH); 2923 (CH_3_); 1591, 1489, 1458 (C=C); 1268 (P=O); 1024 (P-O); 926, 840; 770; 688. Elemental analysis: Calcd. for C_36_H_28_NO_3_P: C, 78.11; H, 5.10; N, 2.53. Found: C, 77.86; H, 5.31; N, 2.47.

*Diphenyl N-benzylamino(pyren-1-yl)methylphosphonate* (**3d**). Y = 0.43 g (77%), yellow solid, m.p. 118–119 °C. ^1^H-NMR (CDCl_3_): δ 8.51–8.49 (m, H_pyr_, 1H); 8.26 (d, ^3^*J*^HH^ = 8.0 Hz, H_pyr_, 1H); 8.24–8.21 (m, H_pyr_, 3H); 8.13–8.09 (m, H_pyr_, 3H); 8.04 (t, ^3^*J*^HH^ = 7.6 Hz, H_pyr_, 1H); 7.29–7.25 (m, H_Ph_, 5H); 7.20–7.19 (m, H_Ph_, 2H); 7.15–7.13 (m, H_Ph_, 3H); 7.02–7.00 (m, H_Ph_, 2H); 6.93 (app. t, ^3^*J*^HH^ = 7.4 Hz, H_Ph_, 1H); 6.73 (app. d, ^3^*J*^HH^ = 8.2 Hz, H_Ph_, 2H); 5.58 (d, ^2^*J*_PH_ = 20.8 Hz, CHP, 1H); 3.93 (d, ^3^*J*^HH^ = 13.3 Hz, NCH_2_, 1H); 3.66 (d, ^3^*J*^HH^ = 13.3 Hz, NCH_2_, 1H). ^13^C-NMR (CDCl_3_): δ 151.0 (d, ^2^*J*_CP_ = 9.9 Hz, C_PO*C*_); 150.5 (d, ^2^*J*_CP_ = 9.7 Hz, C_PO*C*_); 139.1 (C_Ph_); 131.6 (C_pyr_); 131.4 (d, ^4^*J*_CP_ = 3.0 Hz, C_pyr_); 130.8 (C_pyr_); 130.4 (C_pyr_); 130.3 (C_pyr_) 130.0 (C_pyr_); 129.9 (C_pyr_); 129.8 (C_Ph_); 129.5 (C_Ph_); 128.8 (C_Ph_); 128.6 (C_Ph_); 128.2 (C_pyr_); 128.0 (C_pyr_); 127.7 (d, ^4^*J*_CP_ = 1.5 Hz, C_pyr_); 127.5 (C_pyr_); 126.3 (C_pyr_); 125.7 (C_Ph_); 125.5 (d, ^4^*J*_CP_ = 2.8 Hz, C_pyr_); 125.4 (C_Ph_); 125.2 (C_pyr_); 125.0 (C_pyr_); 122.7 (C_pyr_); 120.8 (d, ^3^*J*_CP_ = 4.3 Hz, C_Ph_); 120.3 (d, ^3^*J*_CP_ = 4.3 Hz, C_Ph_); 51.6 (d, ^3^*J*_CP_ = 17.8 Hz, PCN*C*). ^31^P-NMR (CDCl_3_): δ 16.22. IR (KBr): 3386 (νNH); 3041, 2917 (CH_3_); 1616, 1587, 1518, 1451 (C=C); 1245 (P=O); 1066; 1024 (P-O); 941, 843; 777; 618. Elemental analysis: Calcd. for C_36_H_28_NO_3_P: C, 78.11; H, 5.10; N, 2.53. Found: C, 77.81; H, 5.24; N, 2.40.

#### 3.1.2. *Dimethyl N-(R)-α-Methylbenzylamino(pyren-1-yl)methylphosphonate* (**4**)

The reaction was carried out as it is described above, but the post-reaction mixture was dissolved in dichloromethane and washed three times with a saturated aqueous sodium bicarbonate solution. The organic layer was dried, the solvent was removed in vacuo and the residual oil was purified by column chromatography on silica gel with chloroform as eluent. The procedure gave 440 mg of **4** as a dense yellow oil. *Isolated predominant diastereomer*: Y = 0.4 g (92%, after column chromatography), dense yellow oil. $[\alpha {]}_{D}^{20}$ = −48.3° (*c* = 0.1, CHCl_3_). ^1^H-NMR (CDCl_3_): δ 8.29–8.27 (m, H_pyr_, 1H); 8.22 (d, ^3^*J*^HH^ = 8.0 Hz, H_pyr_, 1H); 8.21–8.18 (m, H_pyr_, 3H); 8.10–8.06 (m, H_pyr_, 3H); 8.02 (t, ^3^*J*^HH^ = 7.6 Hz, H_pyr_, 1H); 7.25–7.23 (m, PhH, 2H); 7.22–7.20 (m, PhH, 2H); 7.19–7,16 (m, PhH, 1H); 5.33 (d, ^2^*J*_PH_ = 21.1 Hz, CHP, 1H); 3.91 (q, ^3^*J*^HH^ = 6.5 Hz, *CH*Ph, 1H); 3.86 (d, ^3^*J*_PH_ = 10.5 Hz, POCH_3_, 3H); 3.26 (d, ^3^*J*_PH_ = 10.5 Hz, POCH_3_, 3H); 1.39 (d, ^3^*J*^HH^ = 6.5 Hz, CH*CH_3_*, 3H). ^13^C-NMR (CDCl_3_): δ 145.2 (C_Ph_); 131.6 (C_pyr_); 131.1 (d, ^4^*J*_CP_ = 2.3 Hz, C_pyr_); 130.8 (C_pyr_); 130.3 (C_pyr_); 129.4 (d, ^3^*J*_CP_ = 7.7 Hz, C_pyr_); 128.6 (C_Ph_); 128.2 (C_pyr_); 127.8 (C_pyr_); 127.7 (C_pyr_); 127.3 (C_Ph_); 127.0 (C_Ph_); 126.2 (C_pyr_); 126.1 (C_pyr_); 125.6 (C_pyr_); 125.4 (d, ^4^*J*_CP_ = 2.8 Hz, C_pyr_); 125.3 (C_pyr_); 125.1 (C_pyr_); 125.0 (C_pyr_); 55.8 (d, ^3^*J*_CP_ = 11.9 Hz, PCN*C*); 54.0 (d, ^2^*J*_CP_ = 7.4 Hz, PO*C*); 53.6 (d, ^2^*J*_CP_ = 7.2 Hz, PO*C*); 29.9 (C_aliph_); 27.8 (C_aliph_). ^31^P-NMR (CDCl_3_): δ 26.70. IR (KBr): 3440 (νNH); 3309 (νCH); 2949 (CH_2_); 1600, 1584, 1511, 1454 (C=C); 1242 (P=O); 1055; 1027 (P-O); 846; 815; 761; 609. Elemental analysis: Calcd. for C_27_H_26_NO_3_P: C, 73.12; H, 5.91; N, 3.16. Found: C, 73.15, H, 5.72, N, 2.96. *Mixture of diastereomers—the minor diastereomer is denoted with an asterisk*. ^1^H-NMR (CDCl_3_): δ 8.29–8.28 (m, H_pyr_, 1H); 8.23–8.22 (m, H_pyr_, 1H); 8.21–8.17 (m, H_pyr_, 3H); 8.17–8.16* (m, H_pyr_, 1H); 8.10–8.06 (m, H_pyr_, 3H); 8.03–8.00 (m, H_pyr_, 1H); 7.95–7.94* (m, H_pyr_, 1H); 7.30–7.26* (m, PhH); 7.24–7.22 (m, PhH, 2H); 7.22–7.20 (m, PhH, 2H); 7.19–7.16 (m, PhH, 1H); 7.12–7.10 (m, PhH); 5.33 (d, ^2^*J*_PH_ = 21.0 Hz, CHP, 1H); 4.98* (d, ^2^*J*_PH_ = 21.7 Hz, CHP, 1H); 3.91 (q, ^3^*J*^HH^ = 6.5 Hz, *CH*Ph, 1H); 3.86 (d, ^3^*J*_PH_ = 10.6 Hz, POCH_3_, 3H); 3.83* (d, ^3^*J*_PH_ = 10.5 Hz, POCH_3_, 3H); 3.50* (q, ^3^*J*^HH^ = 6.6Hz, *CH*Ph, 1H); 3.26 (d, ^3^*J*_PH_ = 10.4 Hz, POCH_3_, 3H); 3.23* (d, ^3^*J*_PH_ = 10.4 Hz, POCH_3_, 3H); 1.39 (d, ^3^*J*^HH^ = 6.5 Hz, CH*CH_3_*, 3H); 1.34* (d, ^3^*J*^HH^ = 6.6 Hz, CH*CH_3_*, 3H). ^31^P-NMR (CDCl_3_): δ 26.70 and 26.18* (10:4). Elemental analysis: Calctd for C_27_H_26_NO_3_P: C, 73.12; H, 5.91; N, 3.16. Found: C, 73.17, H, 5.85, N, 3.10.

#### 3.1.3. *Dibenzyl N-(R)-α-Methylbenzylamino(pyren-1-yl)methylphosphonate* (**5**)

The procedure was identical as in a case of aminophosphonate **4**. *Mixture of diastereoisomers—the predominant diastereoisomer is denoted with an asterisk* (^13^C-NMR data are quoted with two decimal digits due to the large number of signals situated very close one to another). Y = 0.12 g, (20%, after column chromatography), dense yellow oil (mixture). ^1^H-NMR (CDCl_3_): δ 8.21–8.20 (m, H_pyr_, 2H); 8.16* (d, ^3^*J*^HH^ = 7.5 Hz, H_pyr_, 1H); 8.15–8.12 (m, H_pyr_); 8.10–8.06 (m, H_pyr_, 4H); 8.03–8.01 (m, H_pyr_, 1H); 7.98* (d, ^3^*J*^HH^ = 9.3 Hz, H_pyr_, 1H); 7.38–7.36 (m, PhH, 2H); 7.34–7.32 (m, PhH, 3H); 7.22–7.17 (m, PhH, 5H); 6.97–6.94* (m, PhH, 2H); 6.92–6.90 (m, PhH); 6.89–6.86* (m, PhH, 2H); 6.83–6.81 (m, PhH); 6.76–6.74 (m, PhH, 2H); 6.68–6.66 (m, PhH); 5.38* (d, ^2^*J*_PH_ = 21.0 Hz, CHP, 1H); 5.26* and 5.10* (Part of ABX system, ^2^*J*_HH_ = 11.8 and ^3^*J*_PH_ = 8.6 and 7.0 Hz, O*CH_2_*Ph, 2H); 5.24–5.22 (m, CHP); 5.13–5.03 (m, O*CH_2_*Ph); 4.72* and 4.39* (part of AMX system, ^2^*J*_HH_ = 11.8 and ^3^*J*_PH_ = 8.4 and 7.3 Hz, O*CH_2_*Ph, 2H); 4.67-4.64 (m, O*CH_2_*Ph); 4.40–4.35 (m, O*CH_2_*Ph); 3.93* (q, ^3^*J*^HH^ = 6.4 Hz, *CH*Ph, 1H); 1.33* (d, ^3^*J*^HH^ = 6.4 Hz, CH*CH_3_*, 3H); 1.30–1.29 (m, CH*CH_3_*). ^13^C-NMR (CDCl_3_): δ 145.21 (C_Ar_); 144.28 (C_Ar_); 136.83 (C_Ar_); 136.79 (C_Ar_); 136.01 and 135.98 (C_Ar_); 131.58 (C_Ar_); 131.06 and 131.05 (C_Ar_); 130.87 (C_Ar_); 130.41 (C_Ar_); 129.57 and 129.52 (C_Ar_); 128.67 and 128.66 (C_Ar_); 128.63 (C_Ar_); 128.45 (C_Ar_); 128.41 (C_Ar_); 128.12 (C_Ar_); 128.03 (C_Ar_); 127.98 (C_Ar_); 127.85 (C_Ar_); 127.75, 127.74, 127.71 and 127.70 (C_Ar_); 127.49 and 127.48 (C_Ar_); 127.43 (C_Ar_); 127.29 (C_Ar_); 126.98 (C_Ar_); 126.13 (C_Ar_); 125.49 (C_Ar_); 125.40 (C_Ar_); 125.32 and 125.30 (C_Ar_); 125.19 (C_Ar_); 125.16 (C_Ar_); 125.09 (C_Ar_); 125.00 (C_Ar_); 124.97 (C_Ar_); 68.79 and 68.75 (PO*C*); 68.64 and 68.60 (PO*C*); 68.34 and 68.30 (PO*C*); 68.10 and 68.06 (PO*C*); 55.94 (C_aliph_); 55.86 (C_aliph_); 55.58 (C_aliph_); 55.46 (C_aliph_); 24.92 (C_aliph_); 22.85 (C_aliph_). ^31^P-NMR (CDCl_3_): δ 25.31*; 24.64 (4:1).Elemental analysis: Calcd. for C_39_H_34_NO_3_P: C, 78.64; H, 5.75; N, 2.35. Found: C, 78.48, H, 5.96, N, 2.16.

#### 3.1.4. *Diphenyl N-(R)-α-Methylbenzylamino(pyren-1-yl)methylphosphonate* (**3e**)

To a solution of the imine **1e** (1mmol) and diphenyl phosphite (1 mmol) in dichloromethane (ca. 2 mL) was slowly added BF_3_·OEt_2_ (24 mg, 0.67 mmol) via syringe. The mixture was stirred at room temperature for 24 h while monitoring the course of the reaction using ^31^P-NMR spectroscopy or TLC. The solvent was removed in vacuo and the residual oil was roughly purified by column chromatography on microcrystalline cellulose with chloroform as eluent to give **3e** as a brown oil in 0.9 g (72%) yield of a crude product. Further attempts to purify and to analyze the product resulted in its decomposition.

### 3.2. Toxicity Tests

#### 3.2.1. Microtox^®^ Toxicity Assay

The method is based on the analysis of light emission reduction of luminescent bacteria (*Vibrio fischeri*) under toxic stress. The tests were carried out in a Microtox^®^ M500 analyzer according to the 1992 Microtox^®^ Manual. The Microtox^®^ Solid-Phase Test (MSPT) was adopted to report of Doe et al. [31]. The MSPT procedure allows the test organisms to come direct contact with the solid sample in an aqueous suspension of the test sample. Thus it is possible to detect toxicity which is due to the insoluble solids that are not in the solution.

All materials and reagents were purchased from MODERNWATER (New Castle, DE, USA). Toxicity was determined by using the marine luminescent bacterium, *Vibrio fischeri*, naturally adapted to a saline environment. Briefly, bacteria were regenerated with 1 mL of Reconstitution Solution (0.01%) and placed in the reagent well of the Microtox^®^. A suspension of 7 g of the sediment was prepared in 35 mL of a Solid Phase Diluent (3.5% NaCl) and was magnetically stirred for 10 min. Then a series of dilutions were made and bacteria (approx. 1 × 10^6^ cell·mL^−1^ per assay) were exposed to these dilutions and to a blank (3.5% NaCl solution) for 20 min. Next, after filtration, the light output of supernatants containing exposed bacteria was measured after 5 min with a Microtox^®^ Analyzer 500. Inhibition was calculated as the concentration of compound loaded to sediment (mg/L) that caused a 50% reduction in the light emitted by the bacteria, and EC_50_ along with 95% confidence limit determined by the software provided with the Analyzer.

#### 3.2.2. Ostracod Test Kit

Ecotoxicity evaluation of synthesized compounds was performed in a short term contact test using Ostracodtoxkit F™ provided by MicroBiotests Inc. (Gent, Belgium). This direct sediment contact bioassay was performed in multiwell test plates using neonates of the benthic ostracod crustacean *Heterocypris incongruens* hatched from cysts [35]. After 6 days of contact with the sediment (or soil) the percentage mortality and the growth of the crustaceans were determined and compared to the results obtained in a non-treated reference sediment (soil).

Briefly, according to manual of Ostracodtoxkit test, the cysts (*Heterocypris incongruens*) were transferred into a Petri dish filled with 10 mL standard fresh water (reconstituted water) and were incubated at 25 °C for 52 h under continuous illumination (approx. 3000–4000 lux). 

After 48 h of cysts incubation, pre-feeding of the freshly hatched ostracods was performed with algae (spirulina-powder) provided in the test kit. Next, after hatching, before feeding with algal food suspension, the length measurements of ostracod neonates has been done. Algae (*Selenastrum capricornutum*) used as feed in the test plate were reconstituted according to the manufacturer’s procedure. Each well of a test plate was filled in the following order: 2 mL standard freshwater, 2 × 500 μL of sediment (soil) treated and non-treated for comparison (blank), 2 mL already prepared algal suspension, 10 ostracods. The test plates were covered with Parafilm^®^ and closed by a lid. Then multiwall plates were incubated at 25 °C in darkness for 6 days. After 6 days of exposure, the ostracods have been recovered from the multiwells to determine the percentage mortality. To calculate the growth inhibition of survived organisms, their length measurements have been also done. Mortality of test organisms was determined in six replicates. The measurement of length was carried out by means of a micrometric strip placed on the bottom of a glass microscope plate. Growth inhibition (GI) of *H. incongruens* in the test sediment was calculated as follows:

% growth inhibition = 100 − [(growth in test sed./growth in ref. sed.) × 100]
(1)

Statistical differences between variables were analyzed with ANOVA.

## 4. Conclusions

To conclude, a series of diphenyl pyrene-derived aminophosphonates **3a**–**d**, **4** and **5** has been prepared. Our previous study [17] revealed that pyrene-derived aminophosphonates have interesting cytotoxic properties against colon cancer cell lines, therefore, the ecotoxicity of the newly synthesized compounds has been evaluated using the crustacean *Heterocypris incongruens* and bacterium *Vibrio fischeri* biotests. The evaluation demonstrated clearly that all compounds **3a**–**d** and **4** are toxic in a moderate to high degree and that diphenyl *N*-benzylamino(pyren-1-yl) methylphosphonate (**3d**) shows the highest degree of ecotoxicity. It has been also revealed that a weak correlation between the toxicity against *H. incongruens* and against *V. fischeri* has been found and the structure-toxicity correlation is rather problematic.

Although no evident correlations between the structure of the tested compounds and their toxicity have been observed, the combination of the different bioassays—the Microtox^®^ and Ostracodtoxkit F™ tests—has been proved to be a very useful tool for determining and comparing the potential toxicities of examined substances **3a**–**d** and **4** as well as for the assessment of their toxicity interactions.

## Figures and Tables

**Table 1:** Microtox^®^ EC_50_ values (mg/kg of soil dry weight) of exposure of the luminescent marine bacteria *Vibrio fischeri* to tested aminophosphonates **3a**–**d** and **4**, with respective 95% confidence limits (in brackets) obtained in the fit of the data.

Compounds	EC_50_ (Lower Limit; Upper Limit)	Coefficient of Determination (R^2^)
**3a**	495.9 (203.4; 1209)	0.7632
**3b**	92.63 (65.27; 131.4)	0.8912
**3c**	214.5 (165.0; 279.0)	0.9452
**3d**	102.7 (81.83; 128.9)	0.9571
**4**	533.1 (439.4; 646.8)	0.9516

## Supplementary Materials

The following are available online at: http://www.mdpi.com/1420-3049/21/7/936/s1, scans of ^1^H, ^13^C and ^31^P NMR of compounds **3a**–**d**, **4** and **5**.
