# Supplementary material for: Synthesis, Spectral Characterization of Several Novel Pyrene-Derived Aminophosphonates and Their Ecotoxicological Evaluation Using Heterocypris incongruens and Vibrio fisheri Tests
Source: Molecules. 2016 Jul 19;21(7):936. doi: 10.3390/molecules21070936 (PMC6273723; doi:10.3390/molecules21070936)
Supplement: Supplementary file 1 [file molecules-21-00936-s001.pdf]

Supplementary Materials: Synthesis, Spectral Characterization of Several Novel Pyrene-Derived Aminophosphonates and Their Ecotoxicological Evaluation Using *Heterocypris incongruens* and *Vibrio fisheri* Tests

Jarosław Lewkowski, Maria Rodriguez Moya, Marta Chmielak, Diana Rogacz, Kamila Lewicka, and Piotr Rychter

Figures 1–7—Spectra of compounds **3a–d**, **4** and **5**.

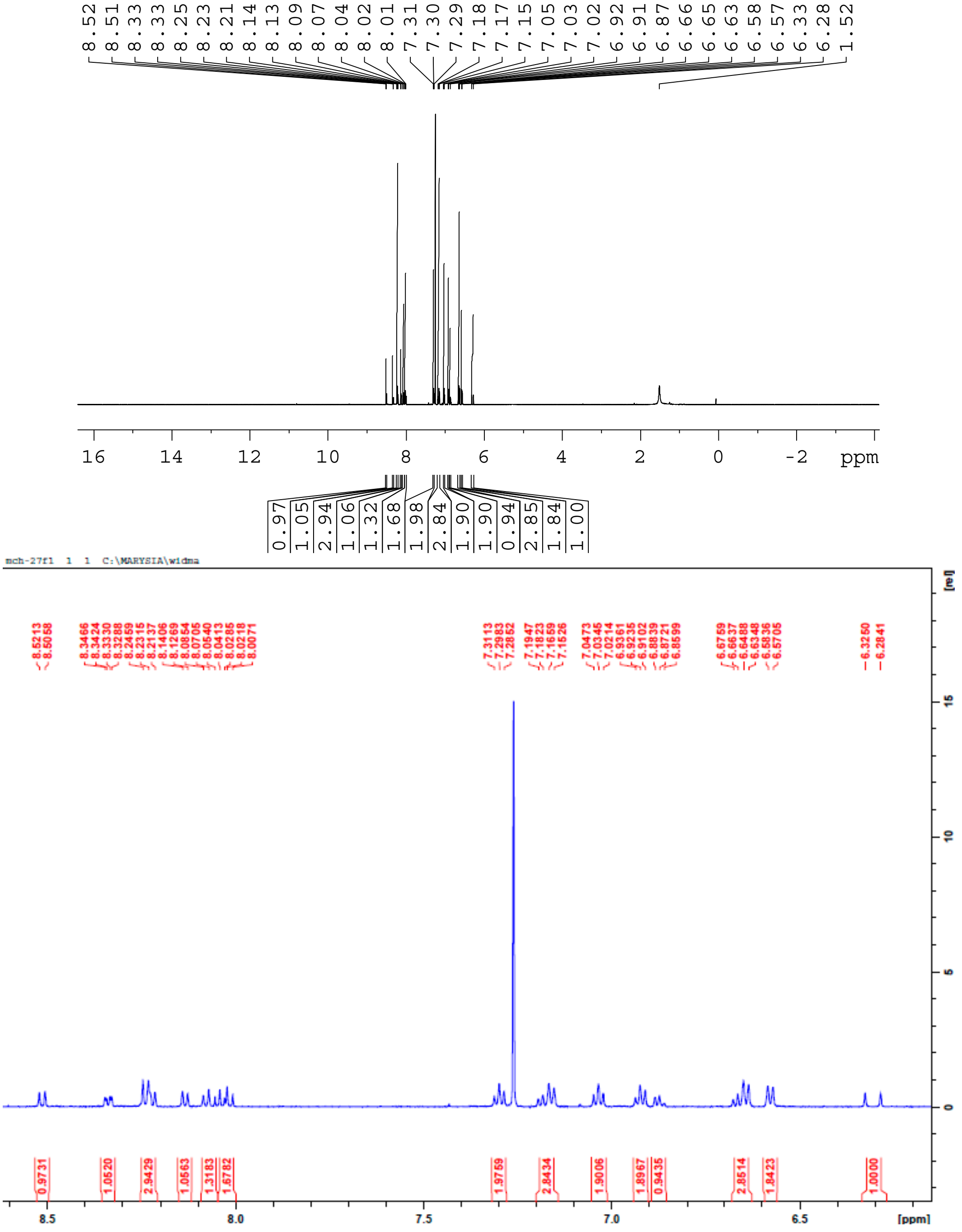

Figure S1. (**a**) Diphenyl *N*-phenylamino(pyren-1-yl)methylphosphonate (**3a**). ^{1}H-NMR—followed by enlarged fragments.

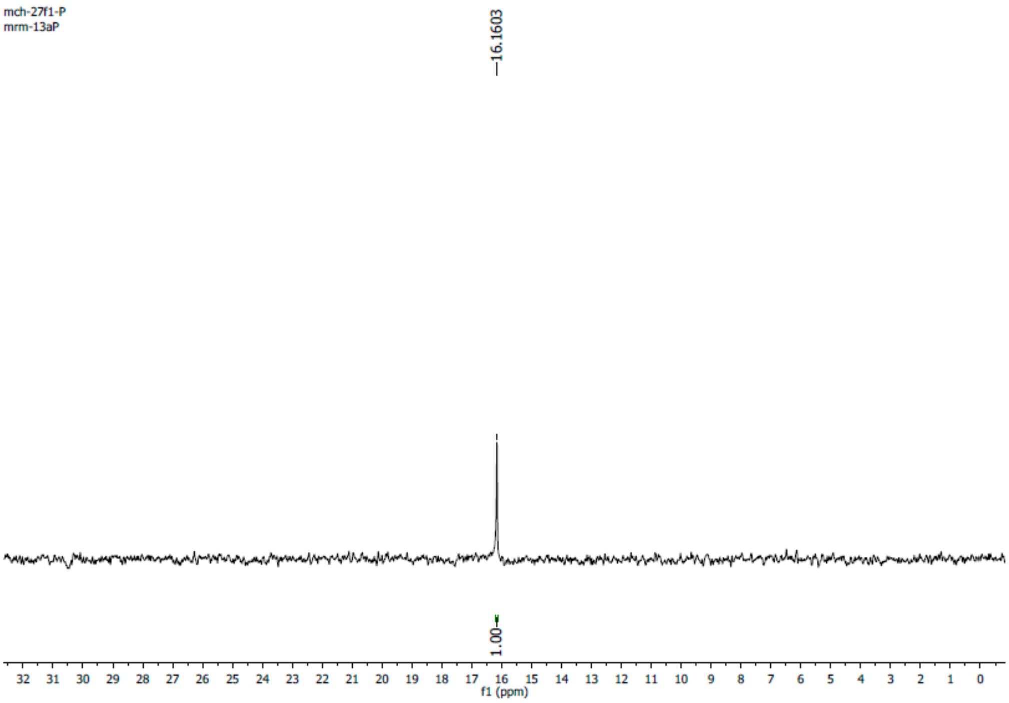

Figure S1. (**b**) Diphenyl *N*-phenylamino(pyren-1-yl)methylphosphonate (**3c**). ^{31}P-NMR.

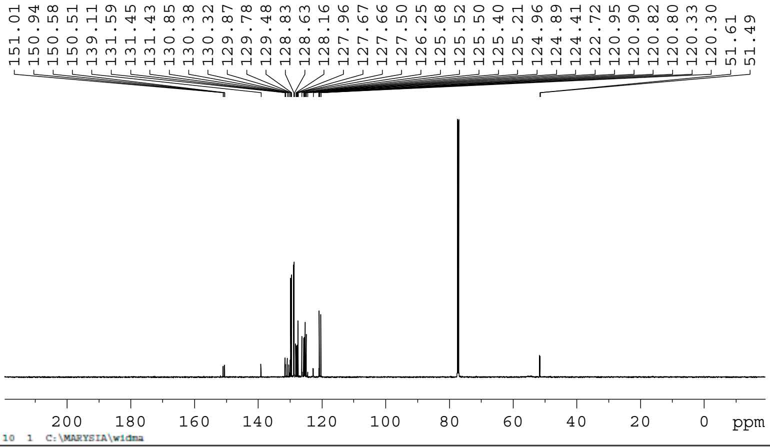

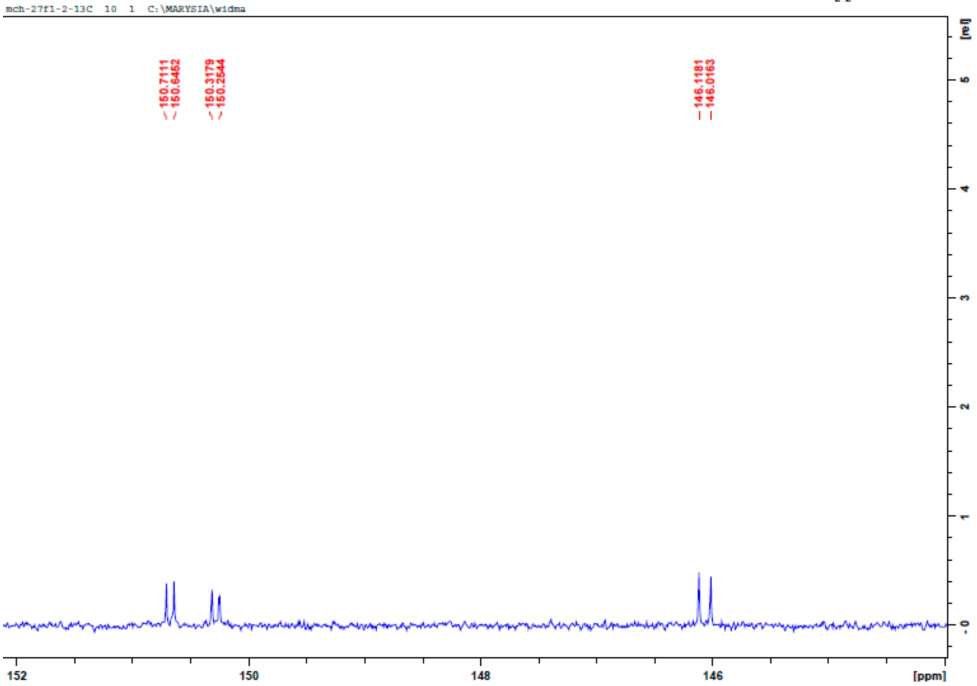

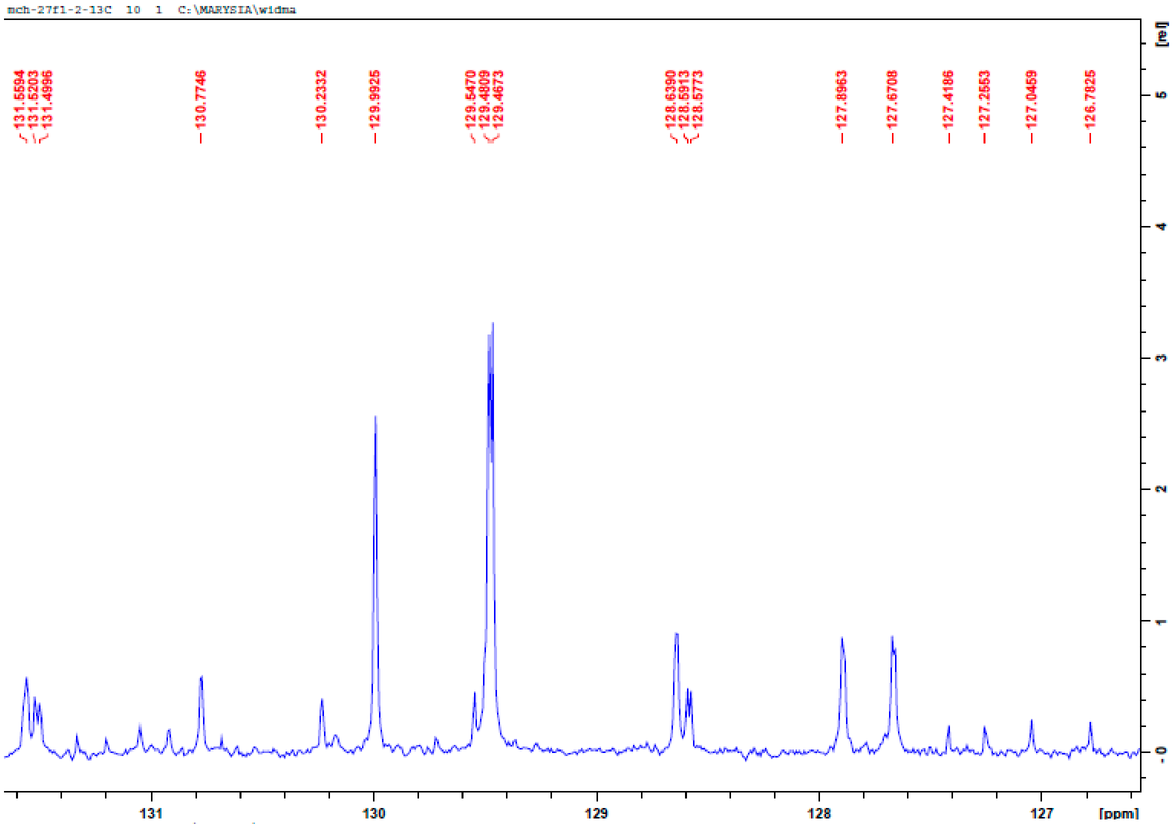
mch-27f1-2-13C 10 1 C:\MARYSIA\widma
131.5594
131.5203
131.4996
130.7746
130.2332
129.9925
129.5470
129.4809
129.4673
128.6390
128.5913
128.5773
127.8963
127.6708
127.4186
127.2553
127.0459
126.7825
[rel]
131
130
129
128
127
[ppm]

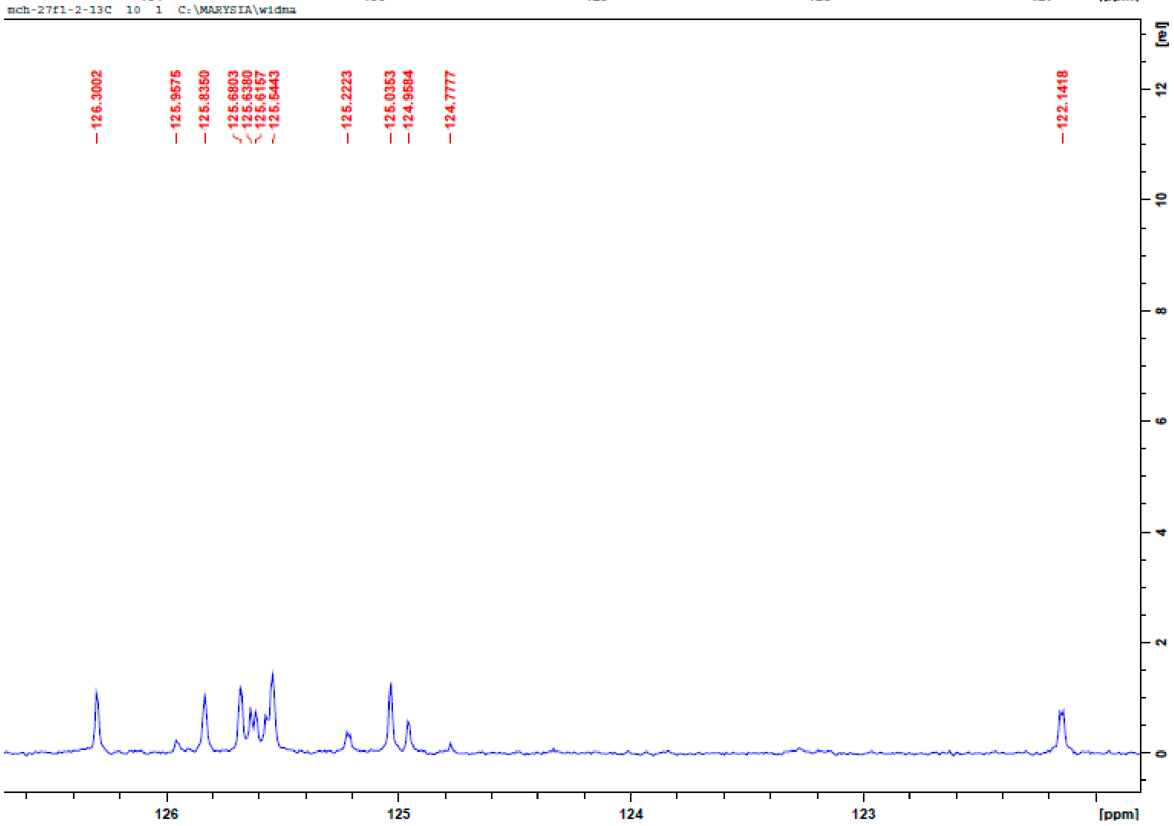
mch-27f1-2-13C 10 1 C:\MARYSIA\widma
126.3002
125.9575
125.8350
125.6803
125.6380
125.6157
125.5443
125.2223
125.0353
124.9584
124.7777
122.1418
[rel]
126
125
124
123
[ppm]

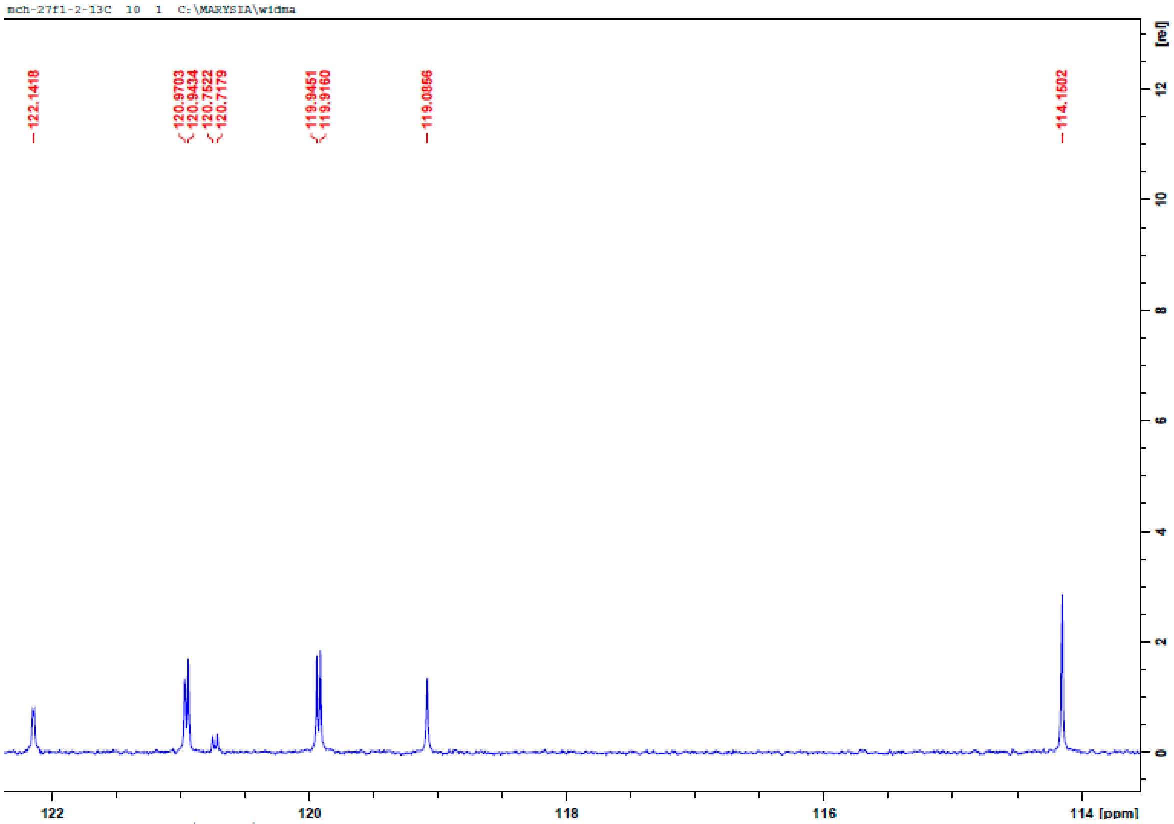

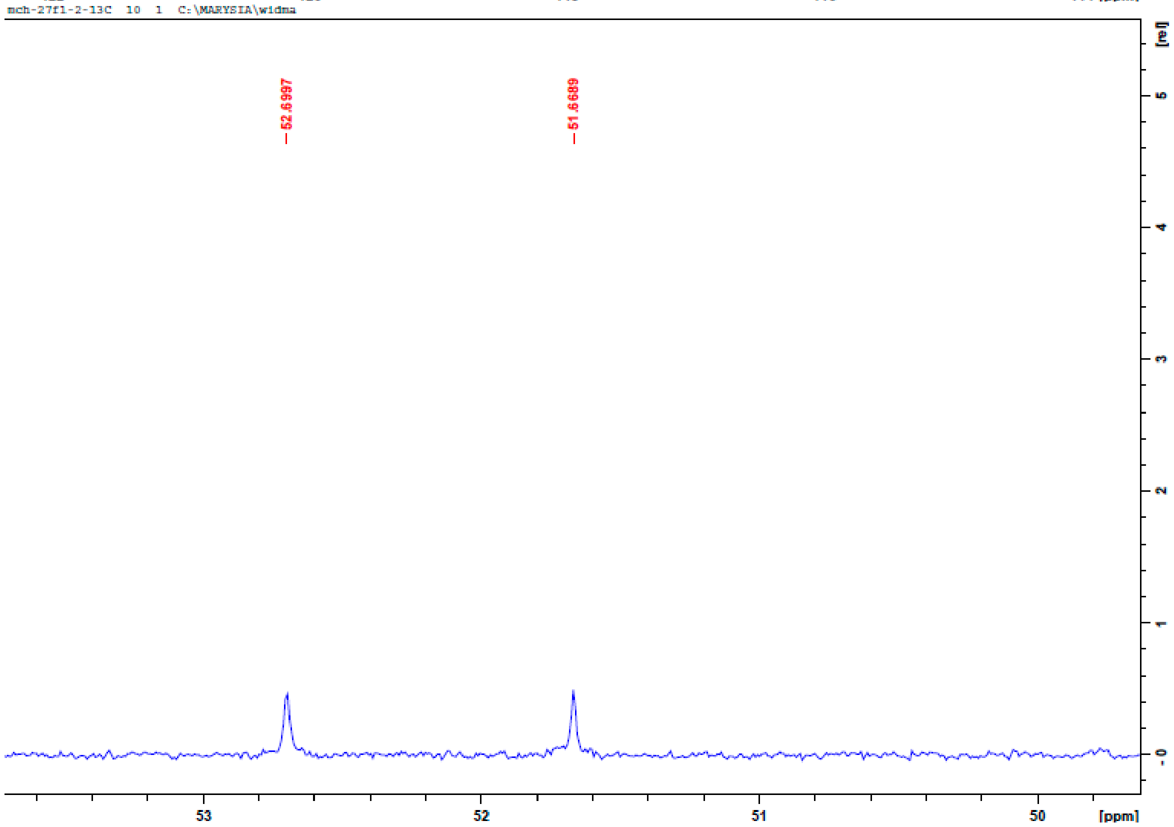

Figure S1. (**c**) Diphenyl *N*-phenylamino(pyren-1-yl)methylphosphonate (**3a**). ^{13}C-NMR—followed by enlarged fragments.

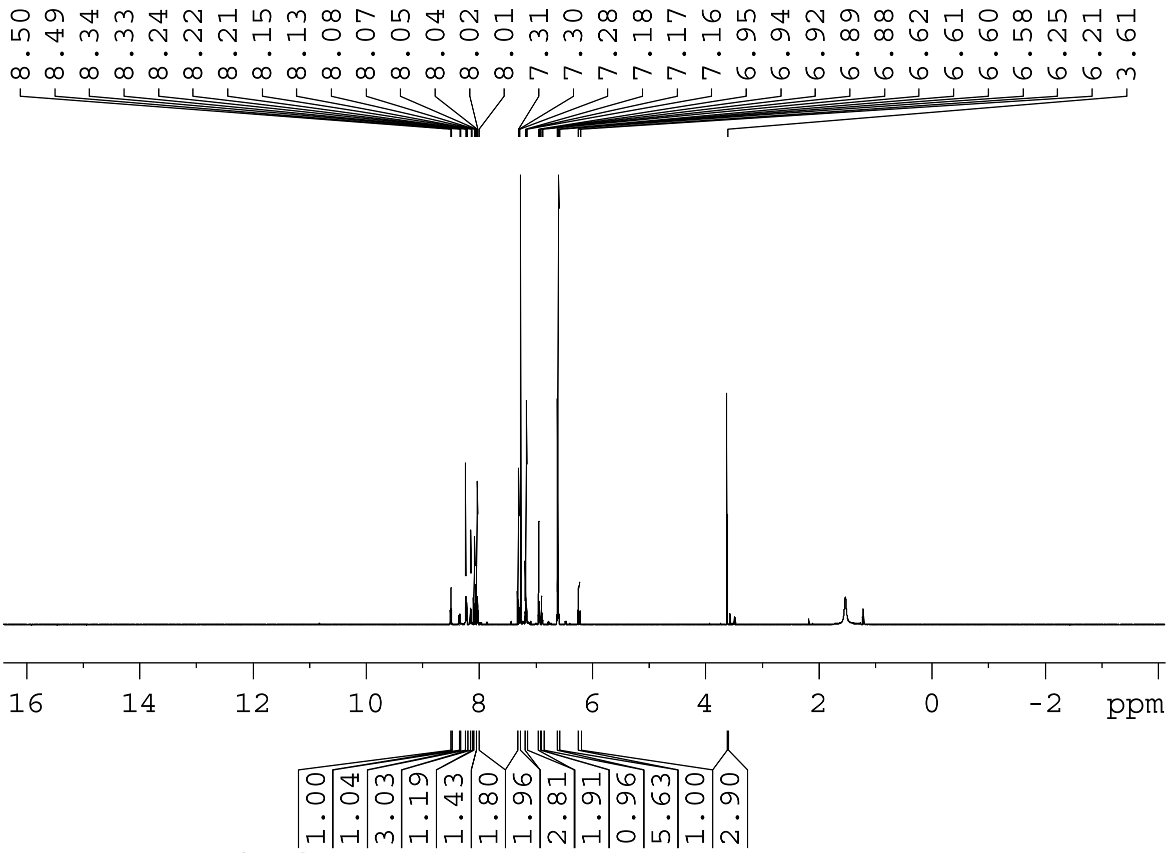
8.50
8.49
8.34
8.33
8.24
8.22
8.21
8.15
8.13
8.08
8.07
8.05
8.04
8.02
8.01
7.31
7.30
7.28
7.18
7.17
7.16
6.95
6.94
6.92
6.89
6.88
6.62
6.61
6.60
6.58
6.25
6.21
3.61
16
14
12
10
8
6
4
2
0
-2
ppm
1.00
1.04
3.03
1.19
1.43
1.80
1.96
2.81
1.91
0.96
5.63
1.00
2.90

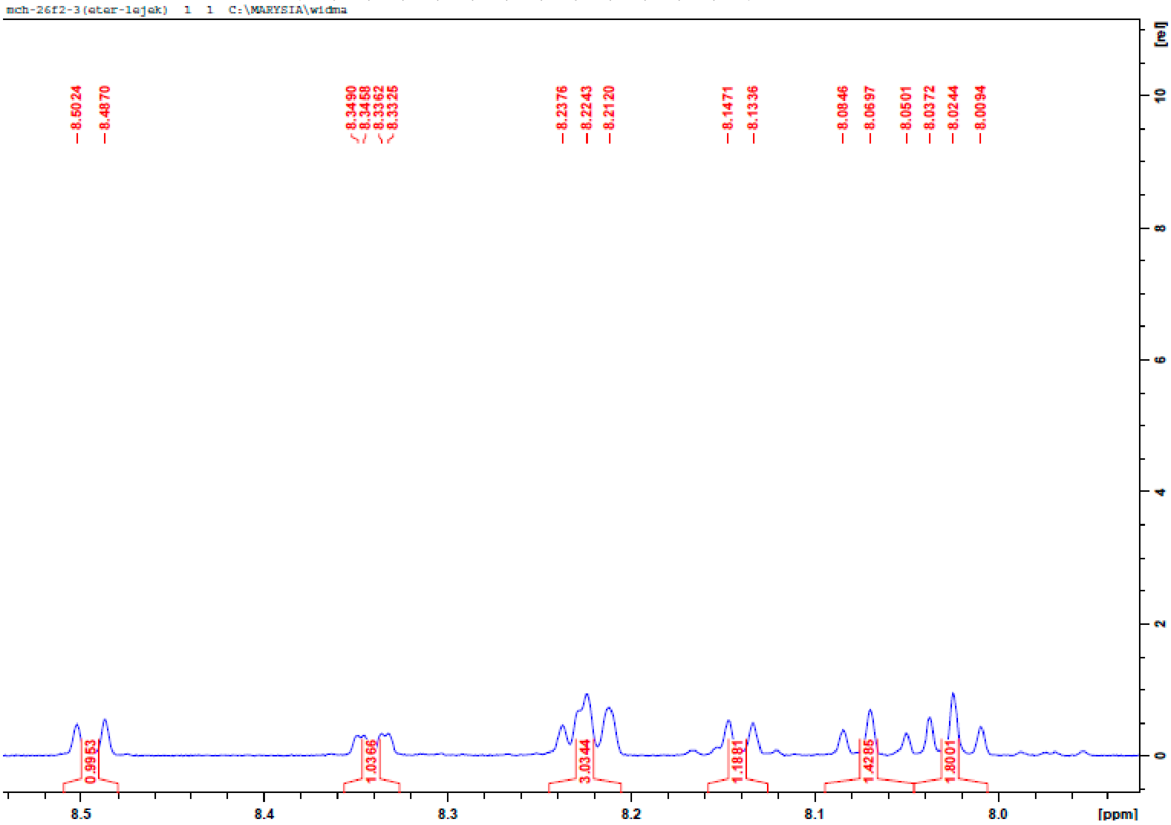
mch-26f2-3(eter-lejek) 1 1 C:\MARYSIA\widma
8.5024
8.4870
8.3490
8.3458
8.3362
8.3325
8.2376
8.2243
8.2120
8.1471
8.1336
8.0846
8.0697
8.0501
8.0372
8.0244
8.0094
0.9953
1.0366
3.0344
1.1881
1.4285
1.8001
8.5
8.4
8.3
8.2
8.1
8.0
[ppm]
[rel]
10
8
6
4
2
0

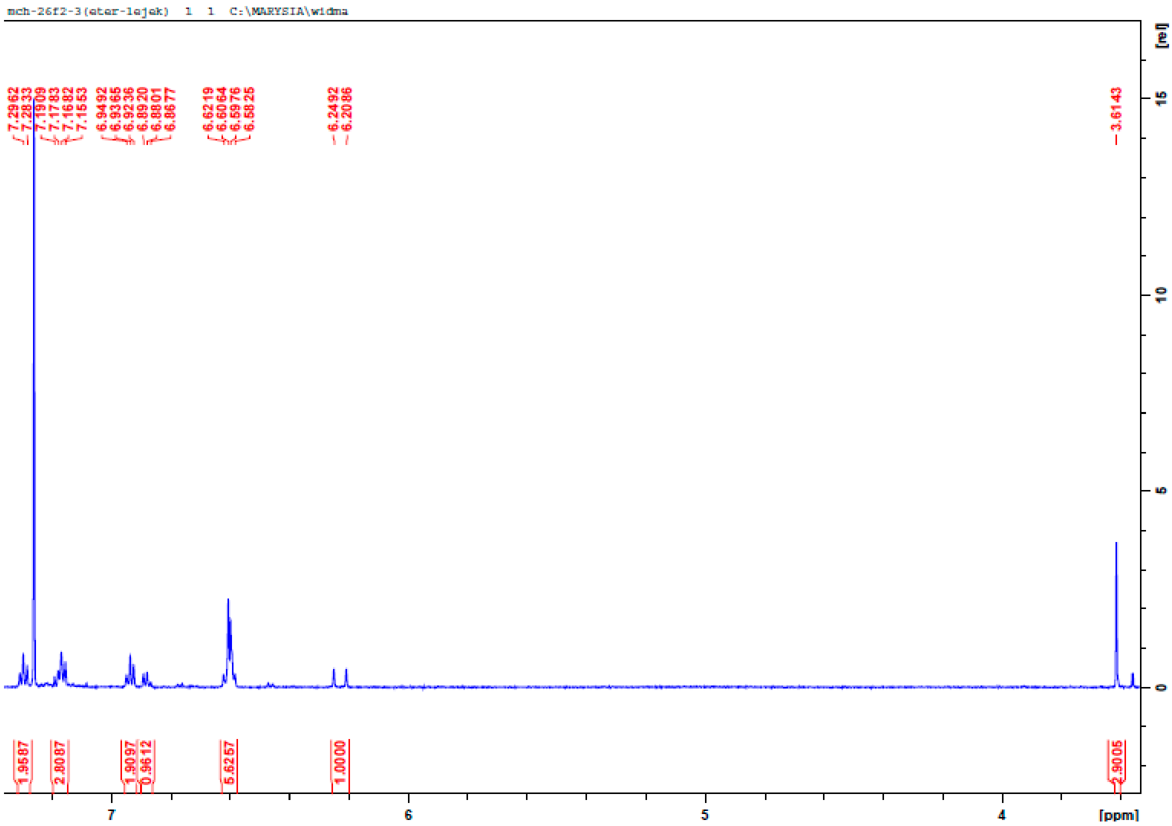

Figure S2. (**a**) Diphenyl *N*-(4-methoxyphenyl)amino(pyren-1-yl)methylphosphonate (**3b**). ^{1}H-NMR—followed by enlarged fragments.

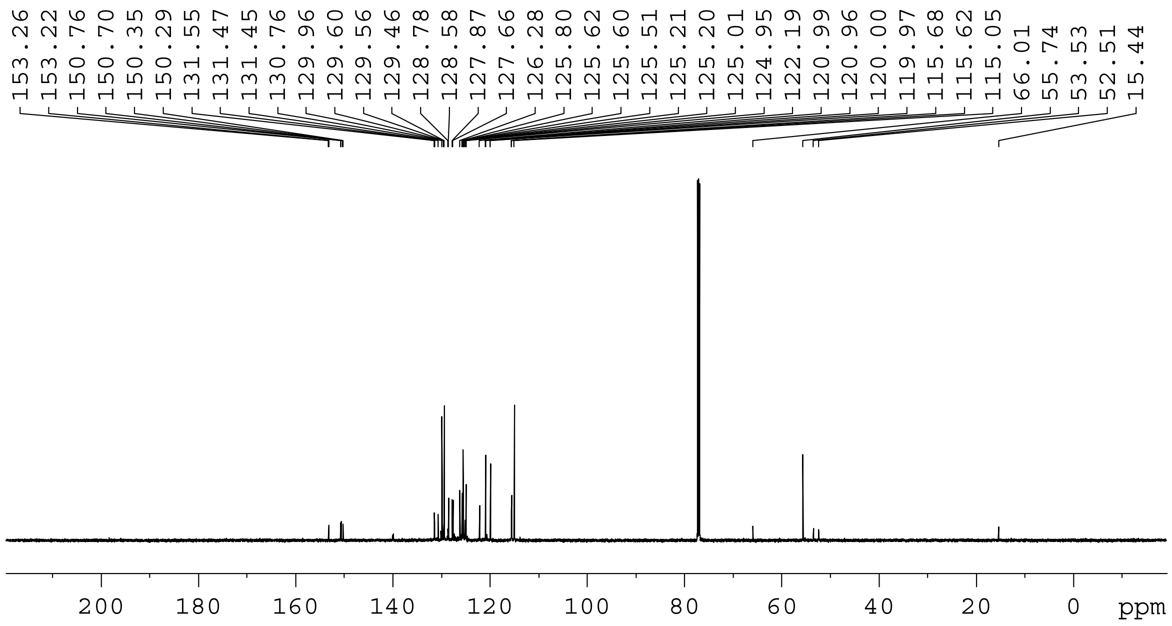

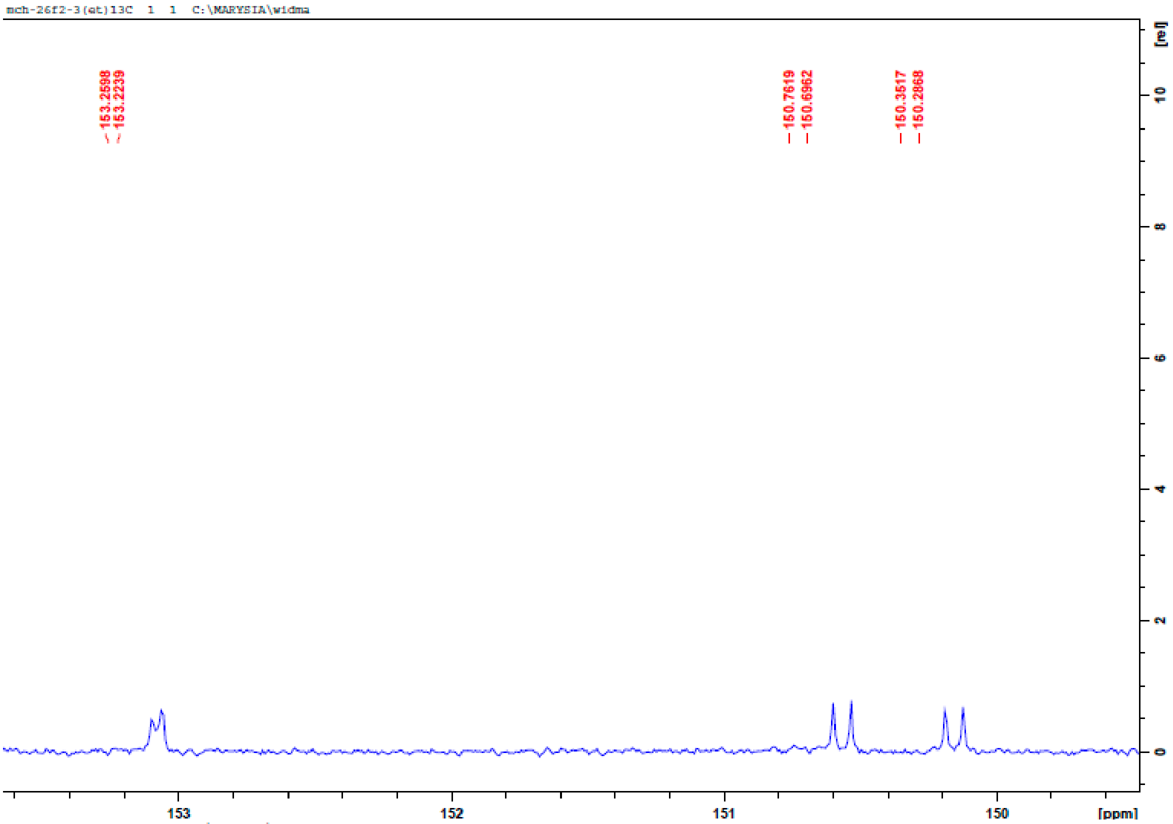
mch-26f2-3(et)13C 1 1 C:\MARYSIA\widma
153.2598
153.2239
150.7619
150.6962
150.3517
150.2868
[rel]
153
152
151
150
[ppm]

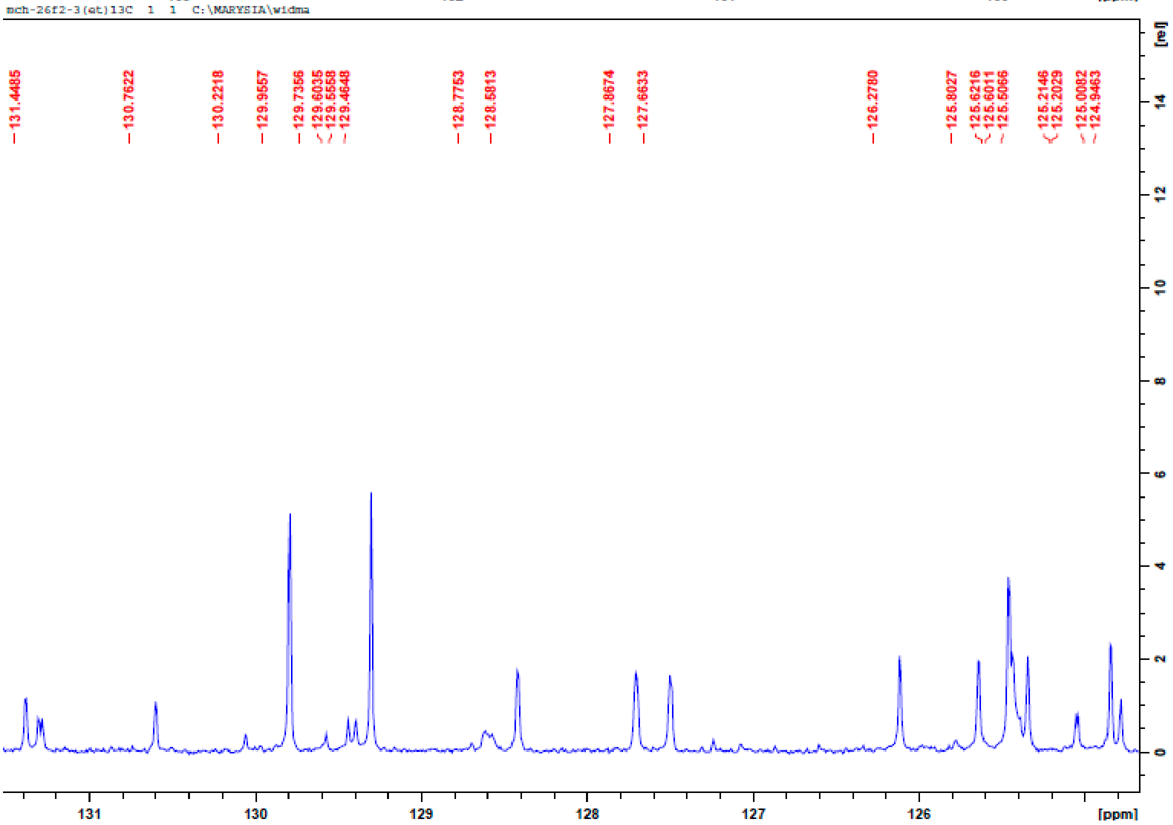
mch-26f2-3(et)13C 1 1 C:\MARYSIA\widma
131.4485
130.7622
130.2218
129.9557
129.7356
129.6035
129.5558
129.4648
128.7753
128.5813
127.8674
127.6633
126.2780
125.8027
125.6216
125.6011
125.5066
125.2146
125.2029
125.0082
124.9463
[rel]
131
130
129
128
127
126
[ppm]

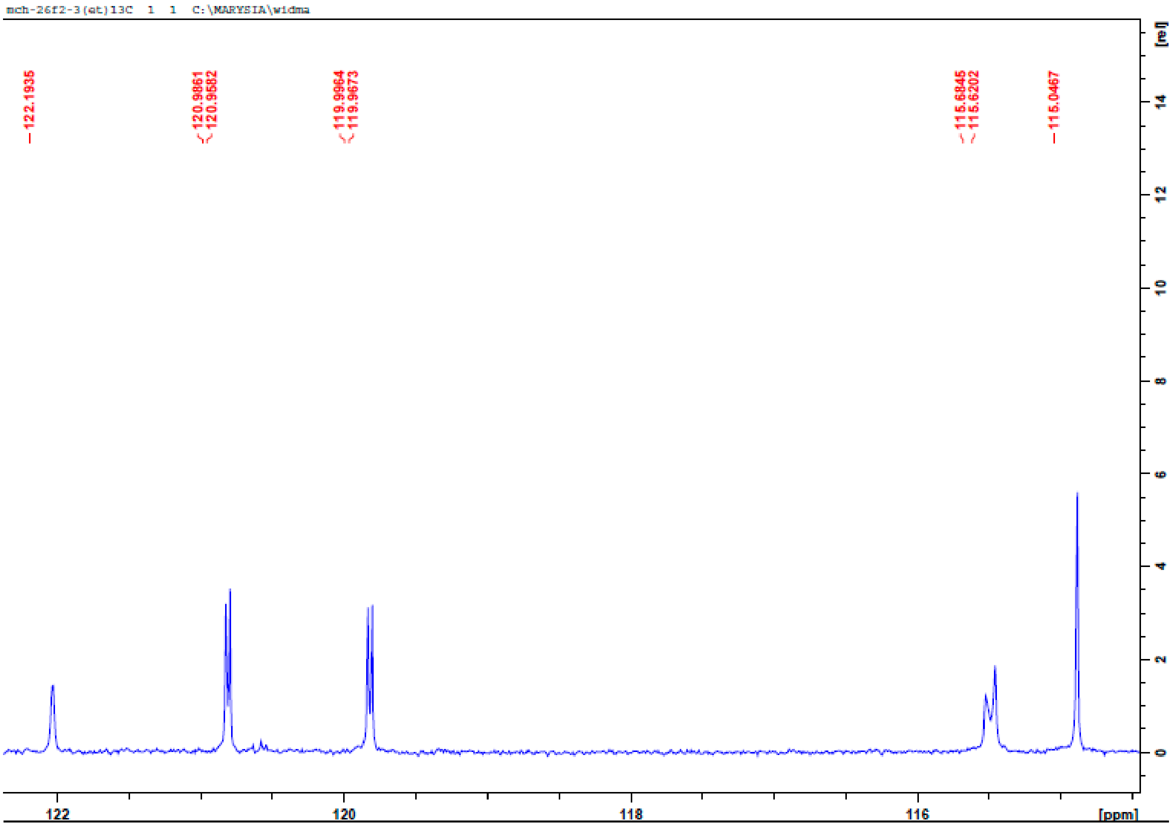

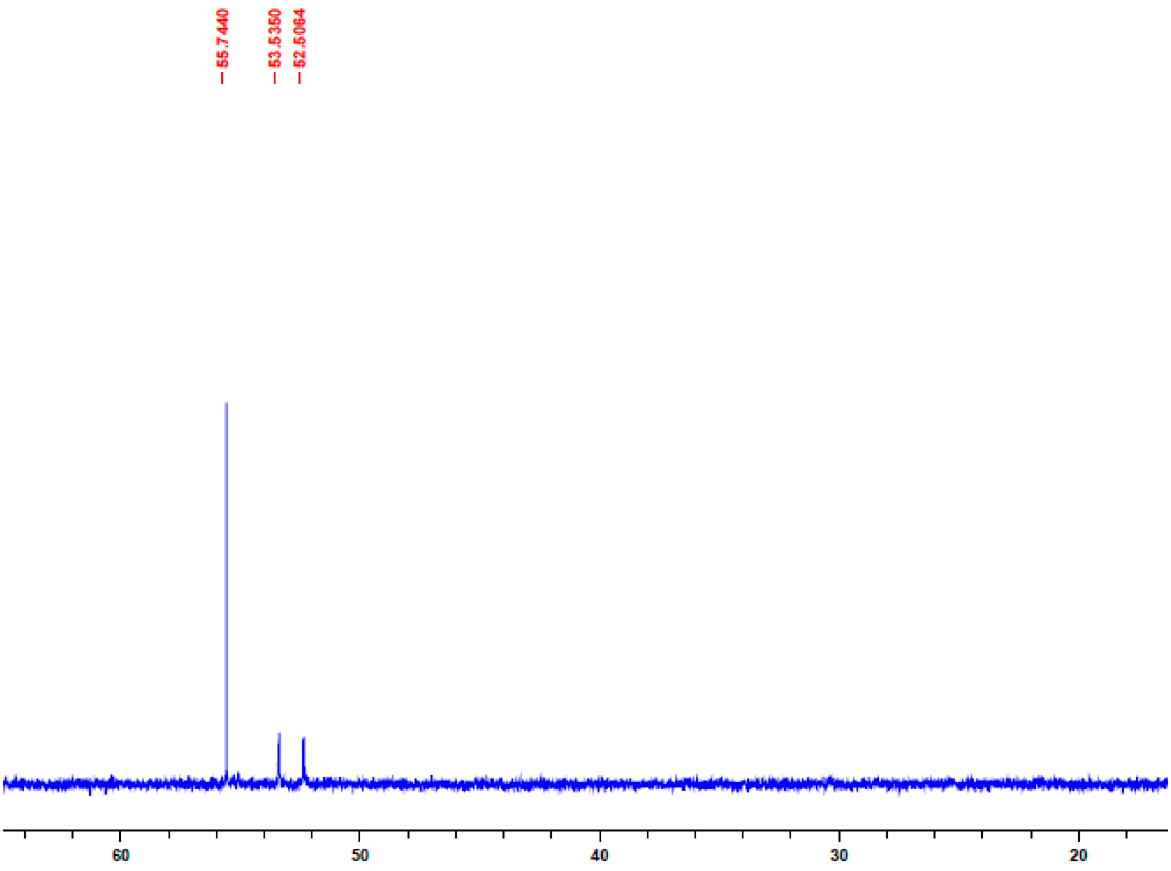

Figure S2. (**b**) Diphenyl *N*-(4-methoxyphenyl)amino(pyren-1-yl)methylphosphonate (**3b**). ^{13}C-NMR—followed by enlarged fragments.

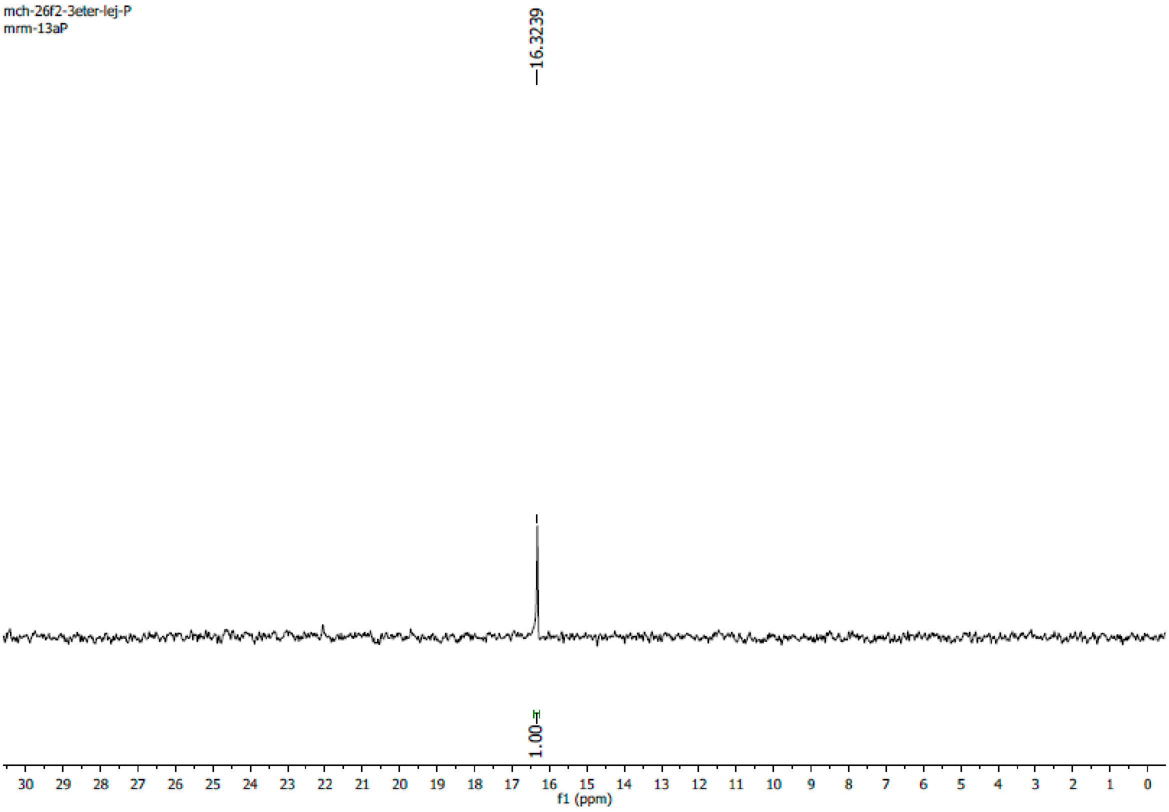

Figure S2. (**c**) Diphenyl *N*-(4-methoxyphenyl)amino(pyren-1-yl)methylphosphonate (**3b**). ^{31}P-NMR.

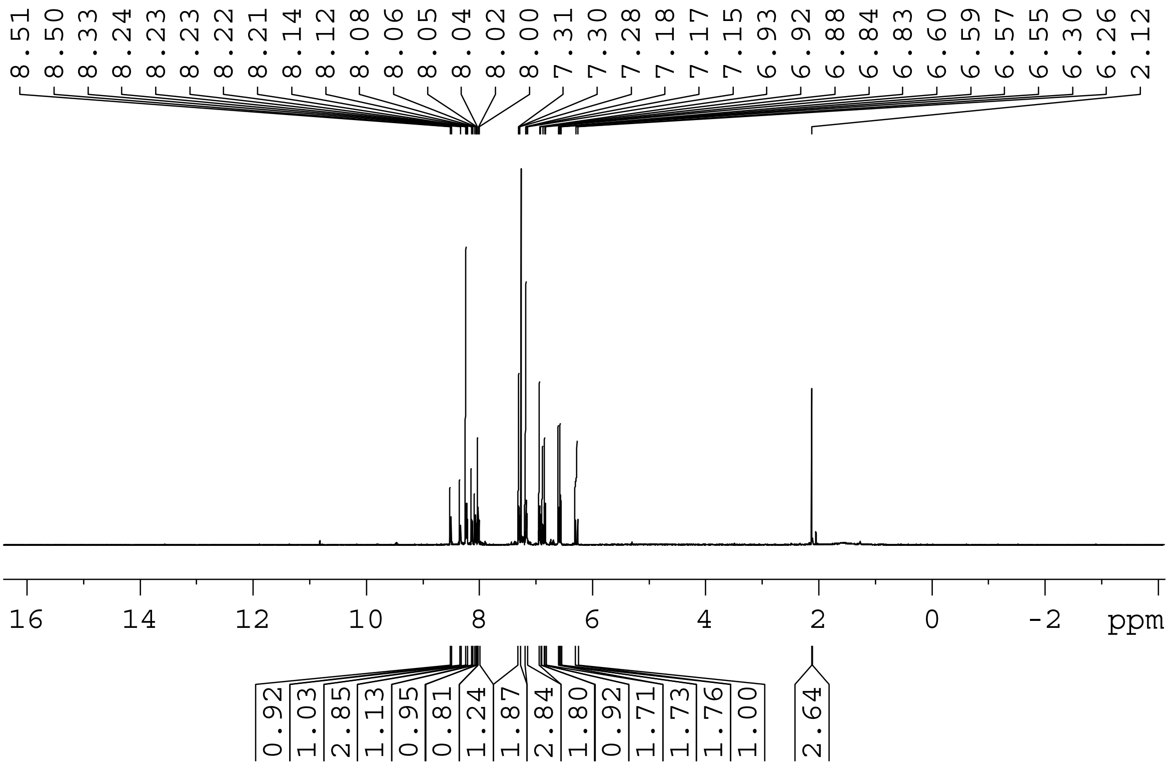

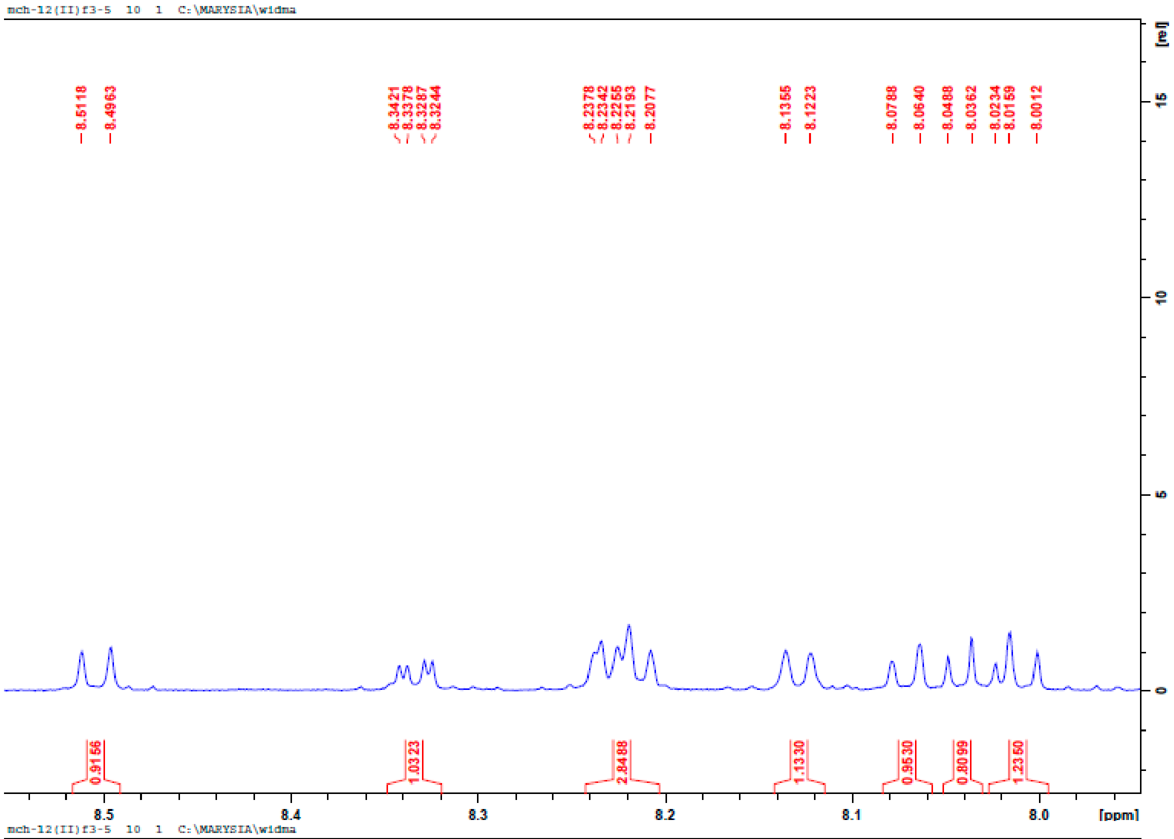
mch-12(II)f3-5 10 1 C:\MARYSIA\widma
8.5118
8.4963
8.3421
8.3378
8.3287
8.3244
8.2378
8.2342
8.2255
8.2193
8.2077
8.1355
8.1223
8.0788
8.0640
8.0488
8.0362
8.0234
8.0159
8.0012
0.9156
1.0323
2.8488
1.1330
0.9530
0.8099
1.2350
[ppm]
[rel]

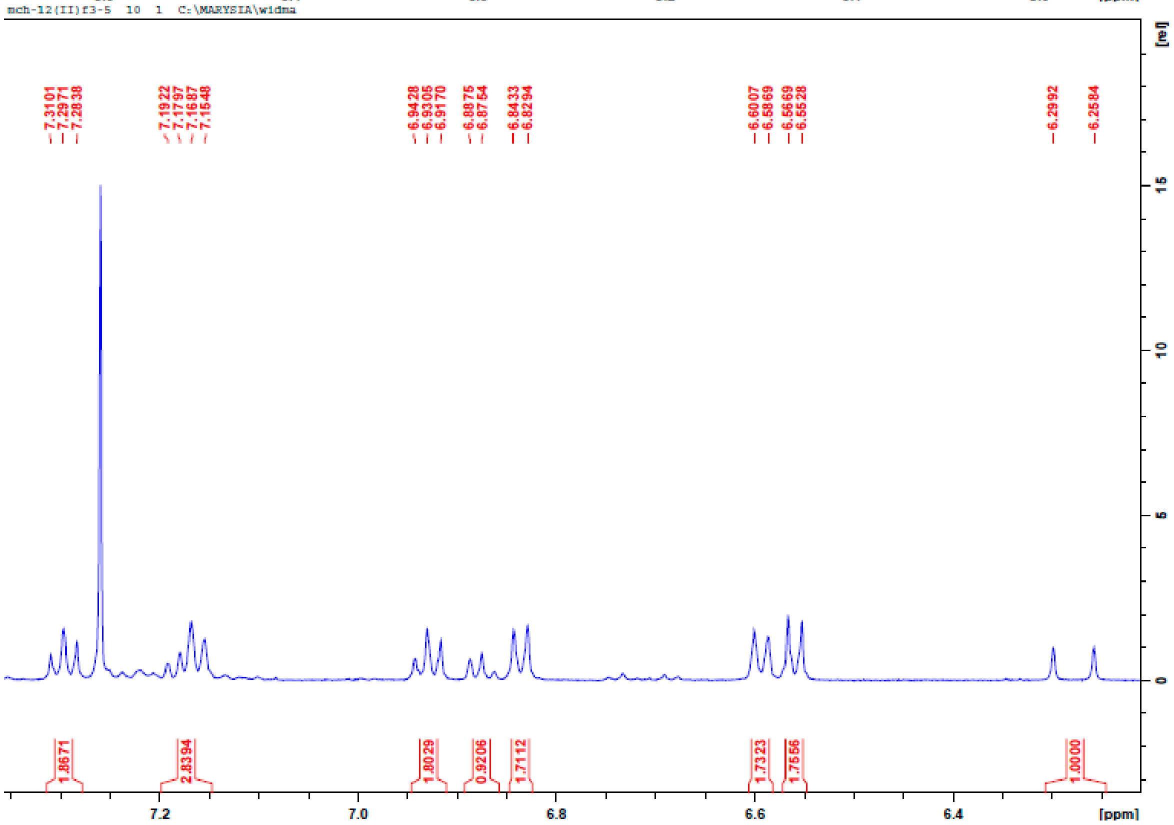
mch-12(II)f3-5 10 1 C:\MARYSIA\widma
7.3101
7.2971
7.2838
7.1922
7.1797
7.1687
7.1548
6.9428
6.9305
6.9170
6.8875
6.8754
6.8433
6.8294
6.6007
6.5869
6.5669
6.5528
6.2992
6.2584
1.8671
2.8394
1.8029
0.9206
1.7112
1.7323
1.7556
1.0000
[ppm]
[rel]

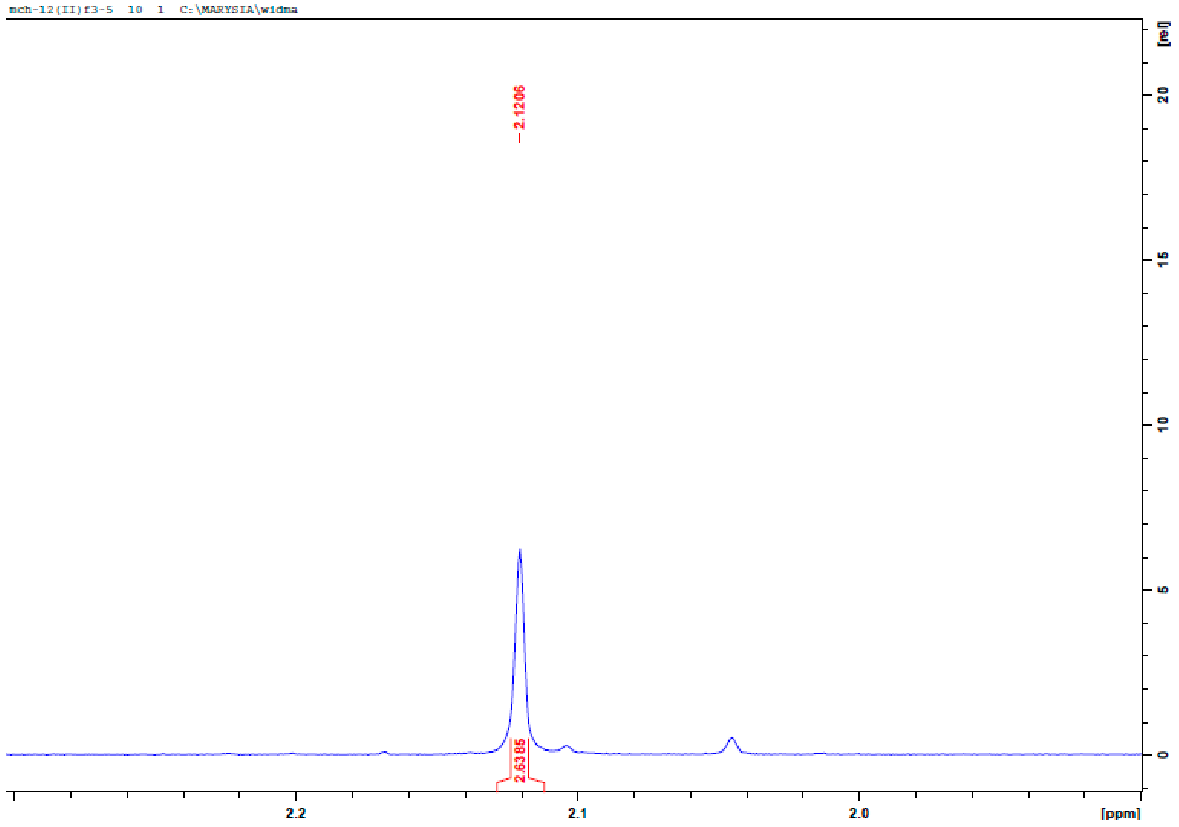

Figure S3. (**a**) Diphenyl *N*-(4-methylphenyl)amino(pyren-1-yl)methylphosphonate (**3c**). ^{1}H-NMR—followed by enlarged fragments.

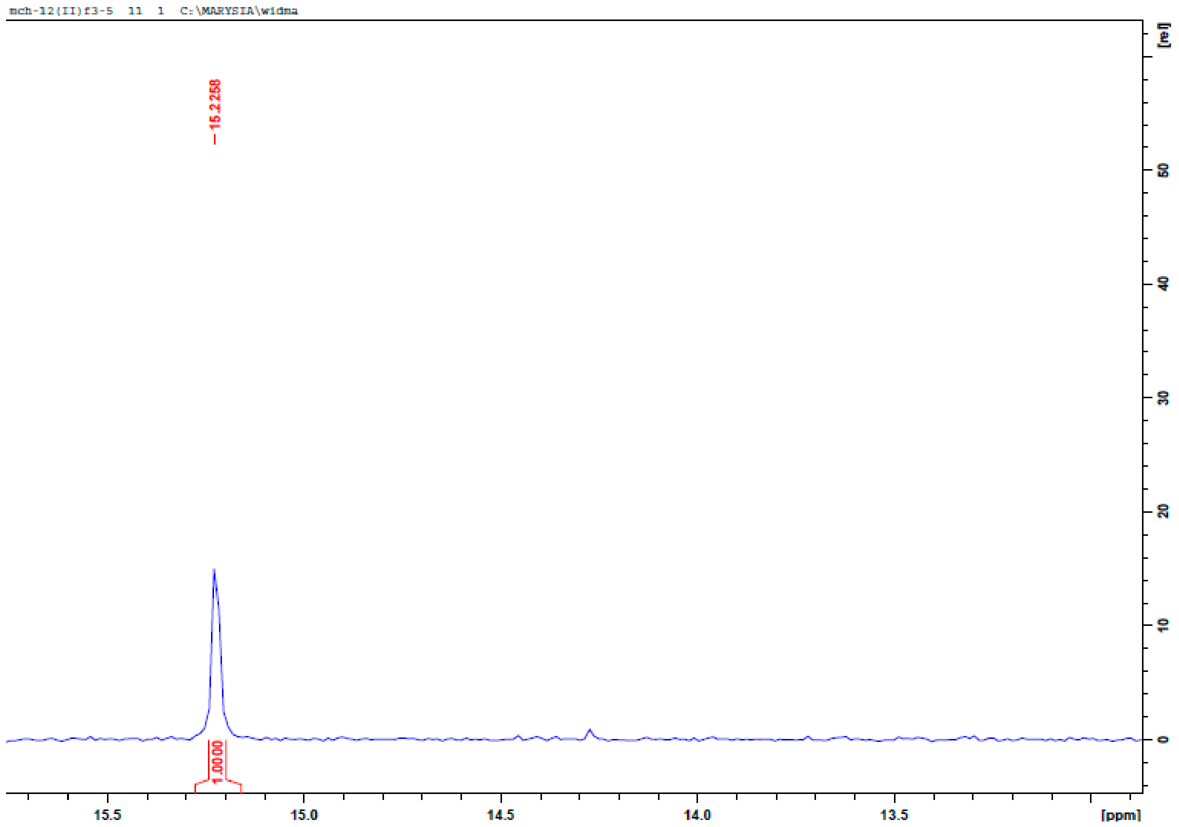

Figure S3. (**b**) Diphenyl *N*-(4-methylphenyl)amino(pyren-1-yl)methylphosphonate (**3c**). ^{31}P-NMR.

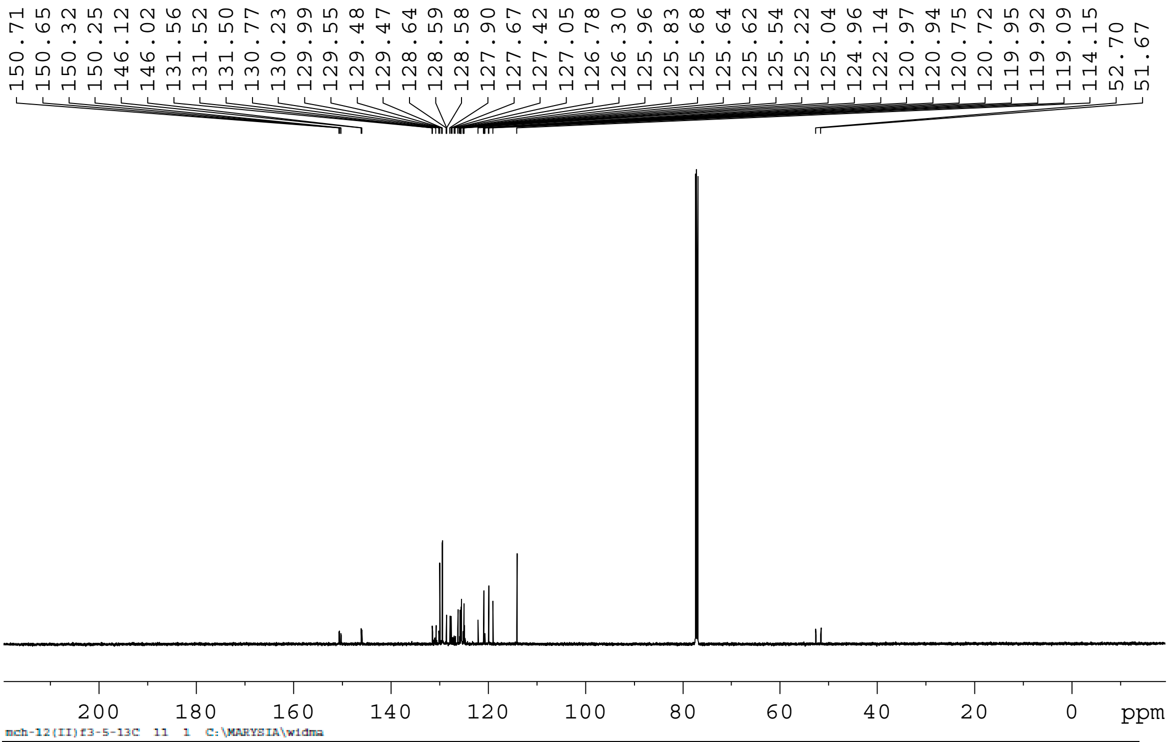

150.71
150.65
150.32
150.25
146.12
146.02
131.56
131.52
131.50
130.77
130.23
129.99
129.55
129.48
129.47
128.64
128.59
128.58
127.90
127.67
127.42
127.05
126.78
126.30
125.96
125.83
125.68
125.64
125.62
125.54
125.22
125.04
124.96
122.14
120.97
120.94
120.75
120.72
119.95
119.92
119.09
114.15
52.70
51.67
200
180
160
140
120
100
80
60
40
20
0
ppm
mch-12(II)f3-5-13C 11 1 C:\MARYSIA\widma

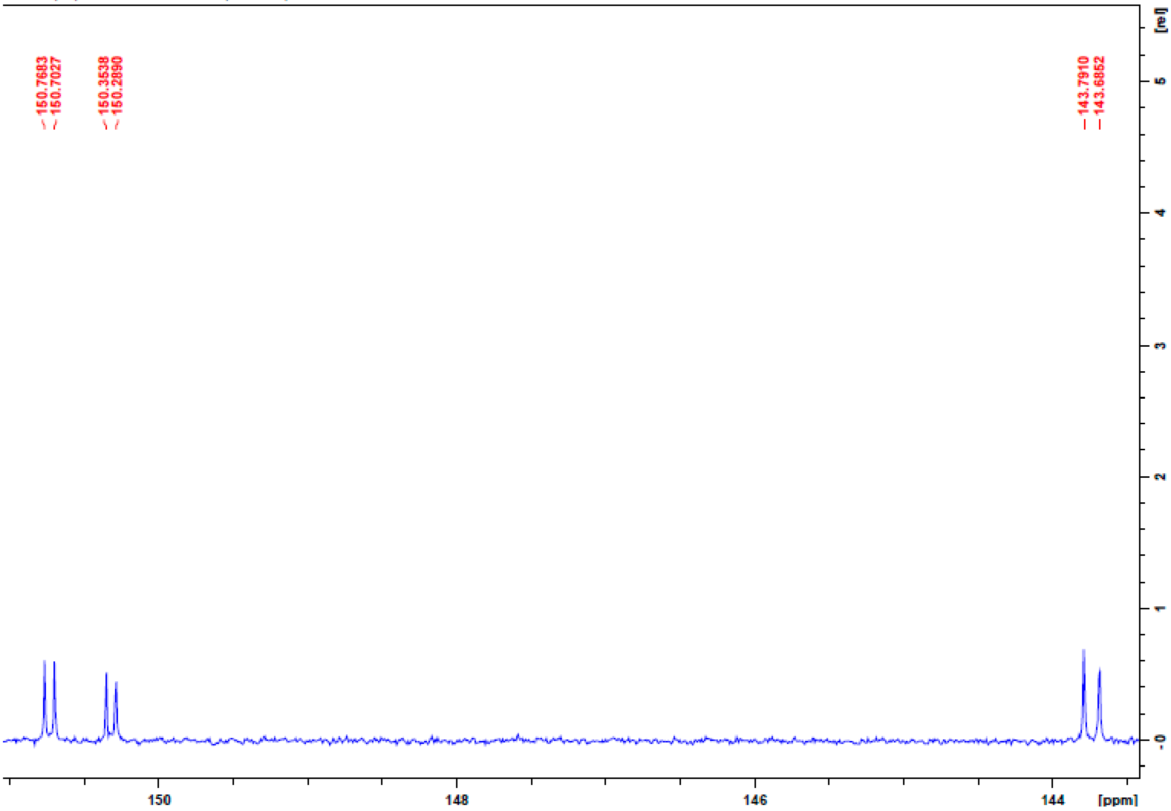

150.7683
150.7027
150.3538
150.2890
143.7910
143.6852
[rel]
5
4
3
2
1
0
150
148
146
144
[ppm]

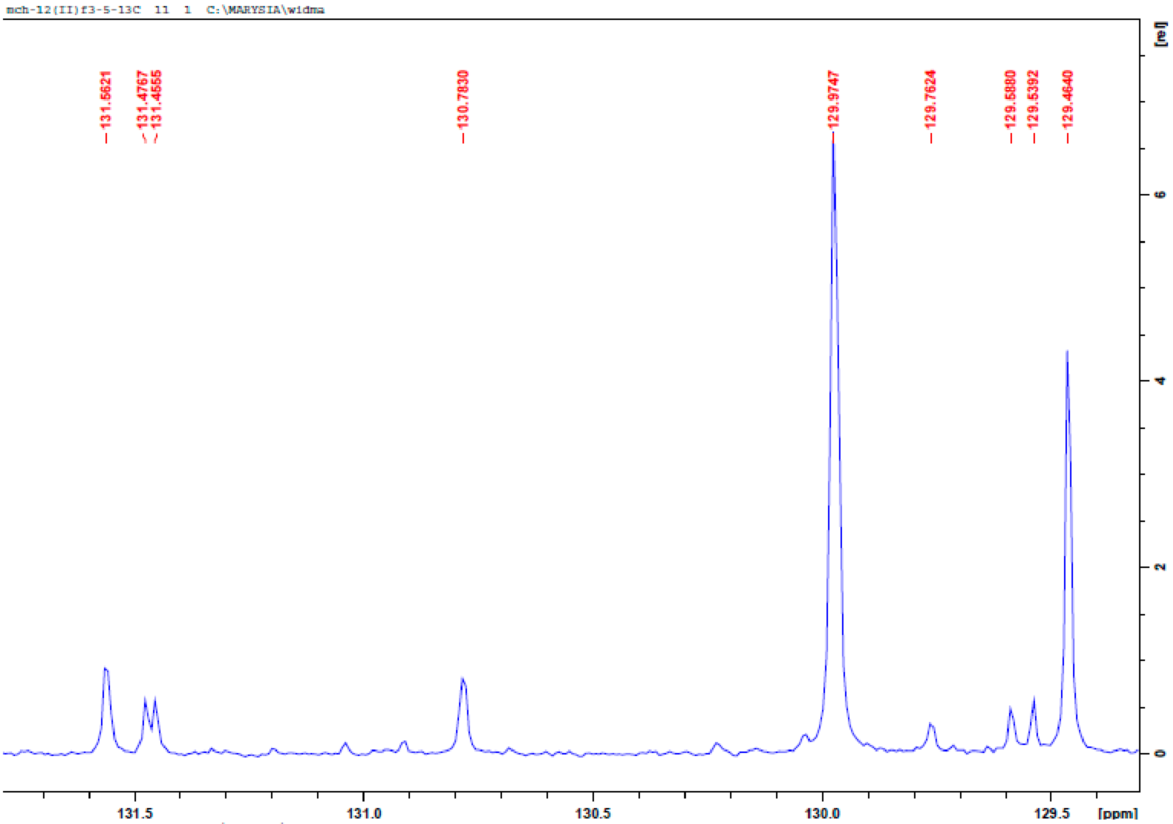
mch-12(II)f3-5-13C 11 1 C:\MARYSIA\widma
131.5621
131.4767
131.4555
130.7830
129.9747
129.7624
129.5880
129.5392
129.4640
131.5
131.0
130.5
130.0
129.5
[ppm]
[rel]

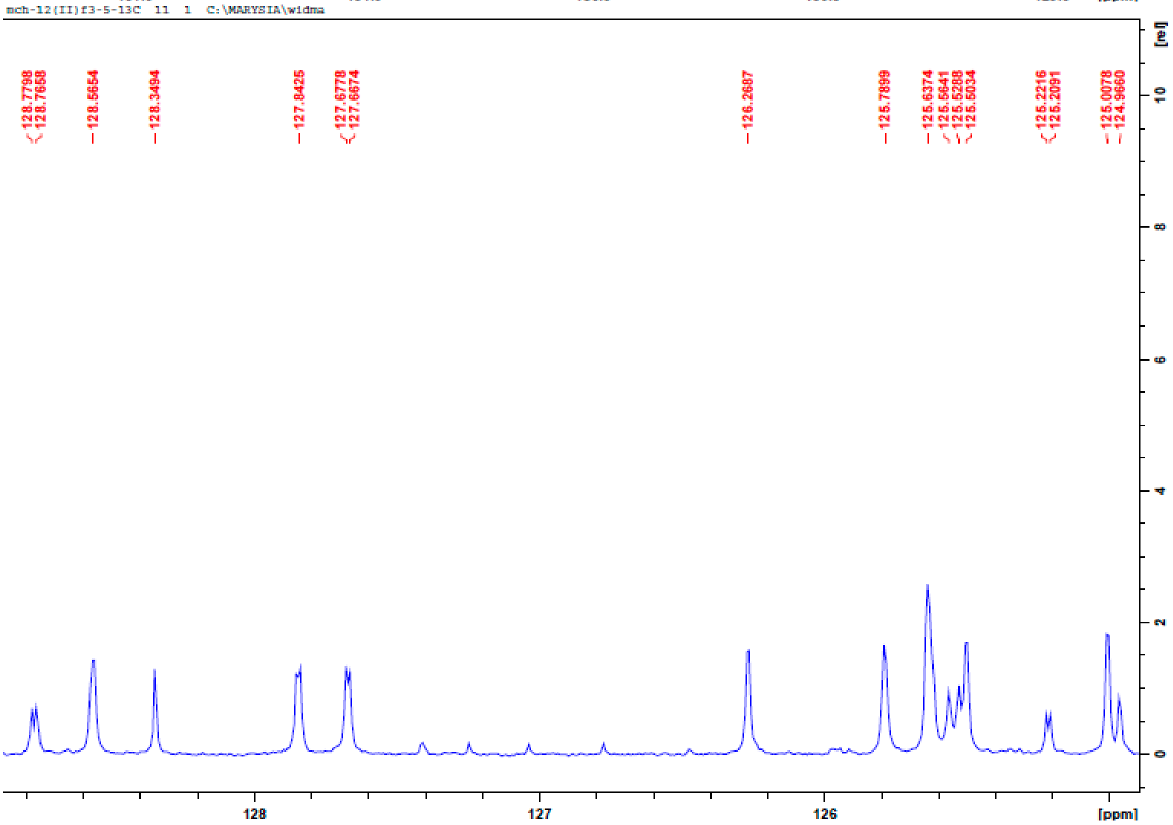
mch-12(II)f3-5-13C 11 1 C:\MARYSIA\widma
128.7798
128.7658
128.5654
128.3494
127.8425
127.6778
127.6674
126.2687
125.7899
125.6374
125.5641
125.5288
125.5034
125.2216
125.2091
125.0078
124.9660
128
127
126
[ppm]
[rel]

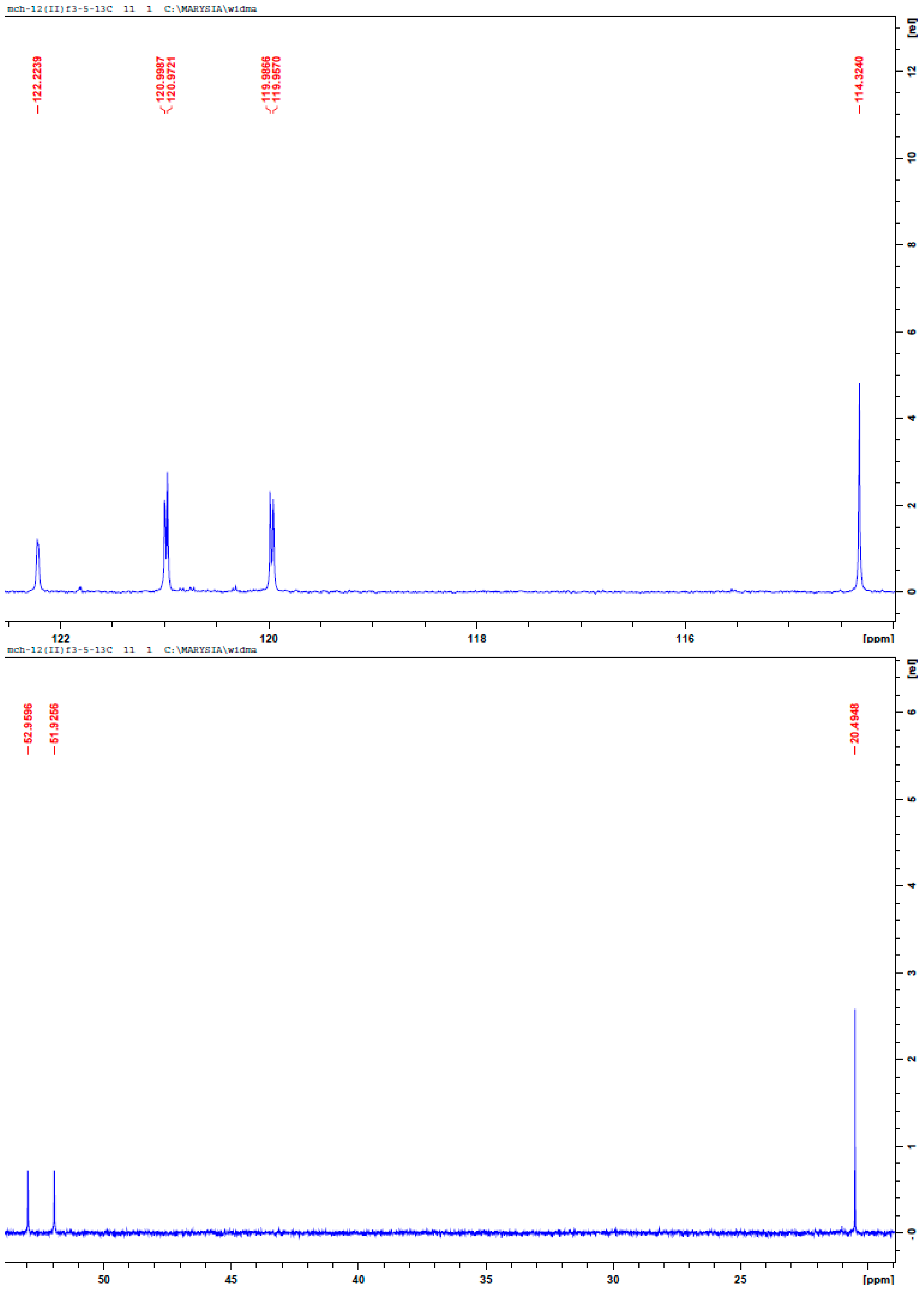

Figure S3. (**c**) Diphenyl *N*-(4-methylphenyl)amino(pyren-1-yl)methylphosphonate (**3c**). ^{13}C-NMR—followed by enlarged fragments.

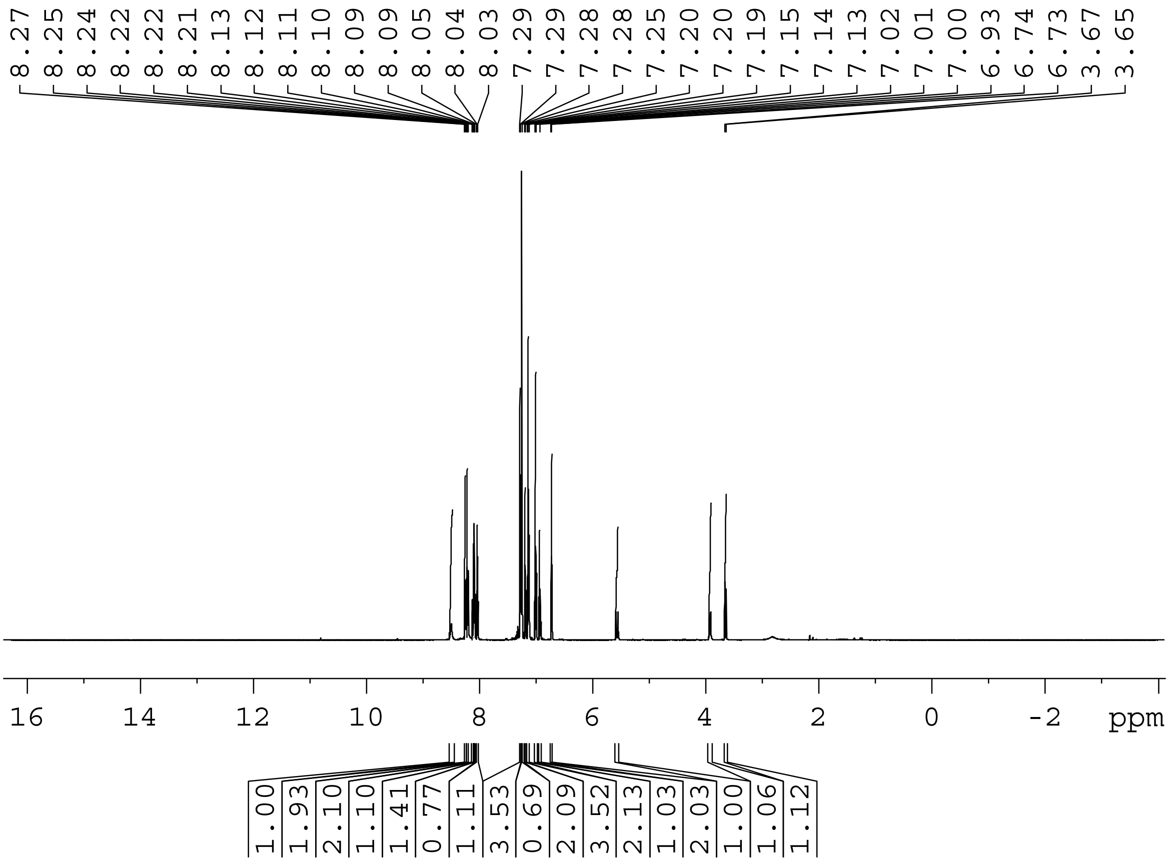

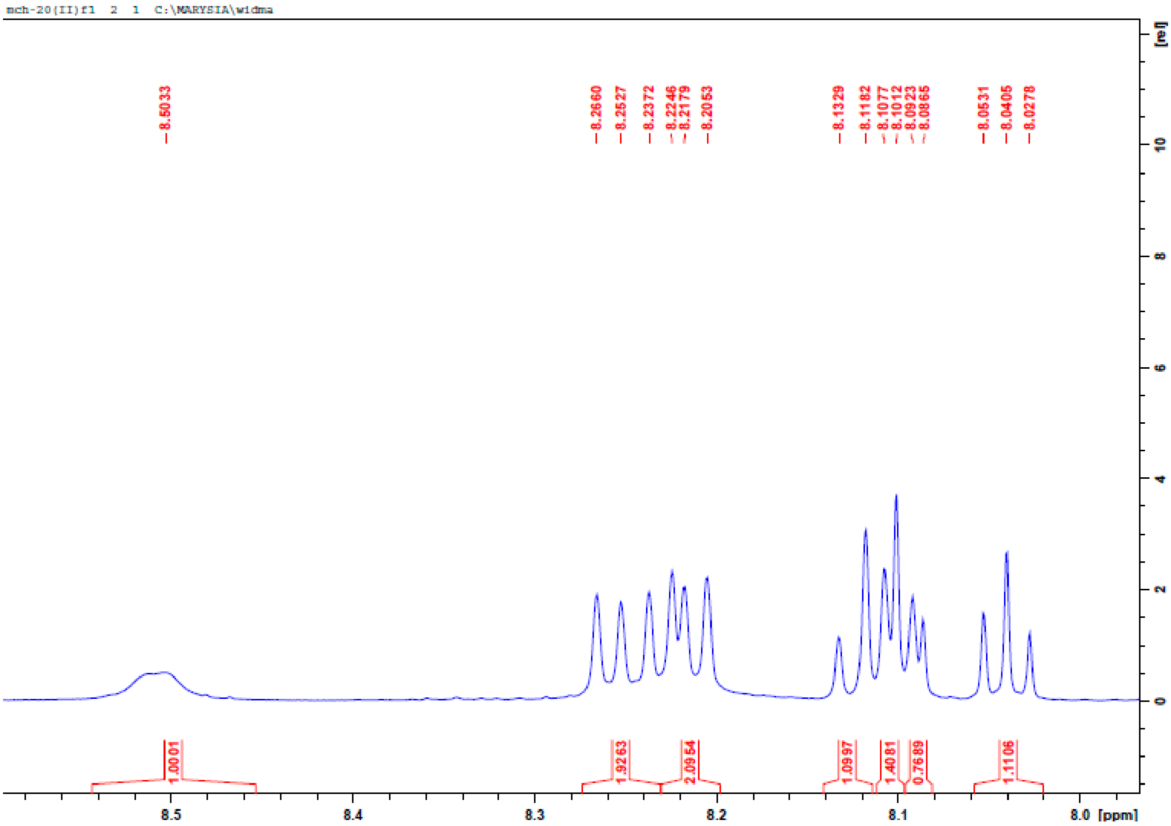
mch-20(II)f1 2 1 C:\MARYSIA\widma

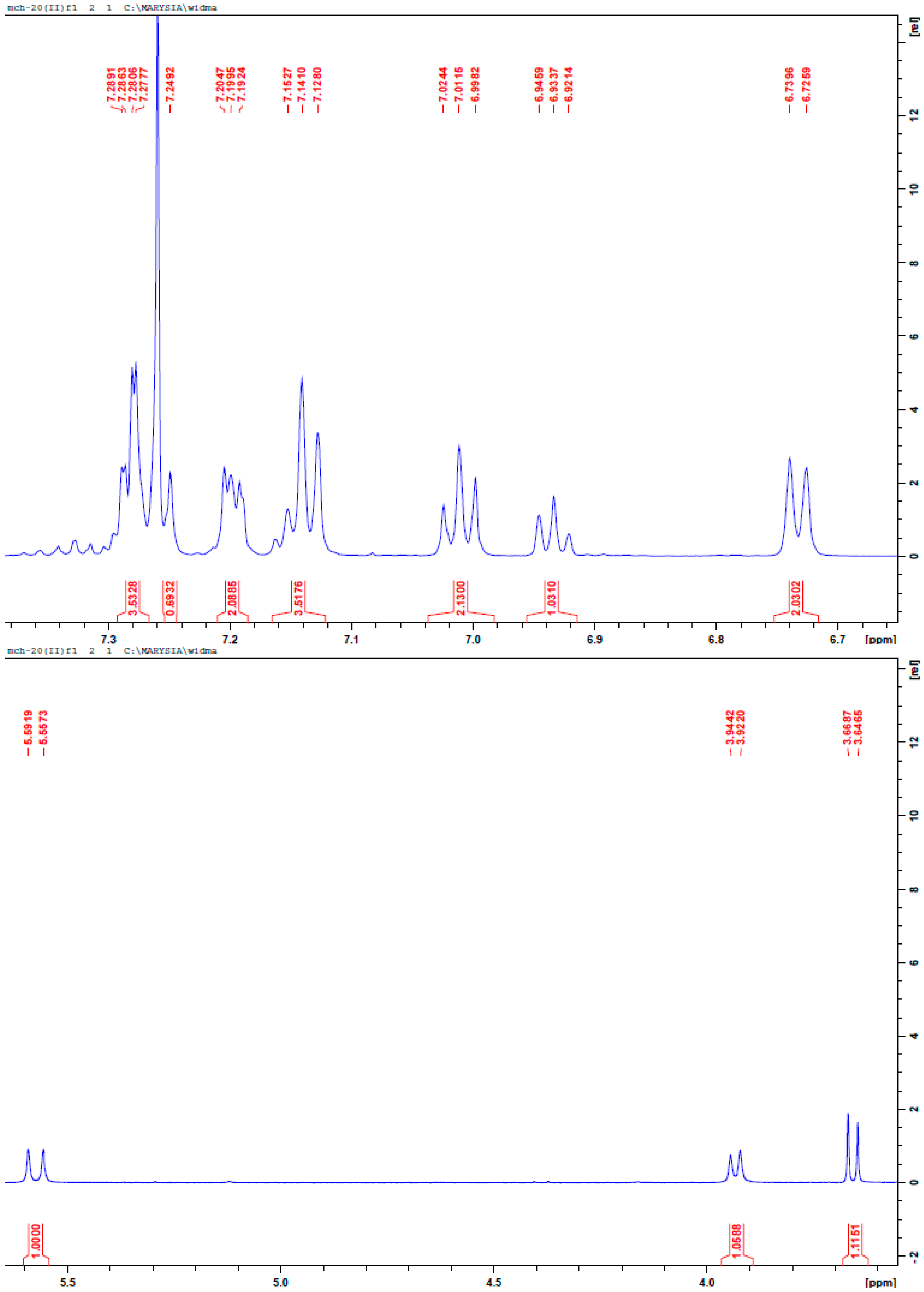

Figure S4. (**a**)Diphenyl *N*-benzylamino(pyren-1-yl)methylphosphonate (**3d**). ^{1}H-NMR—followed by enlarged fragments.

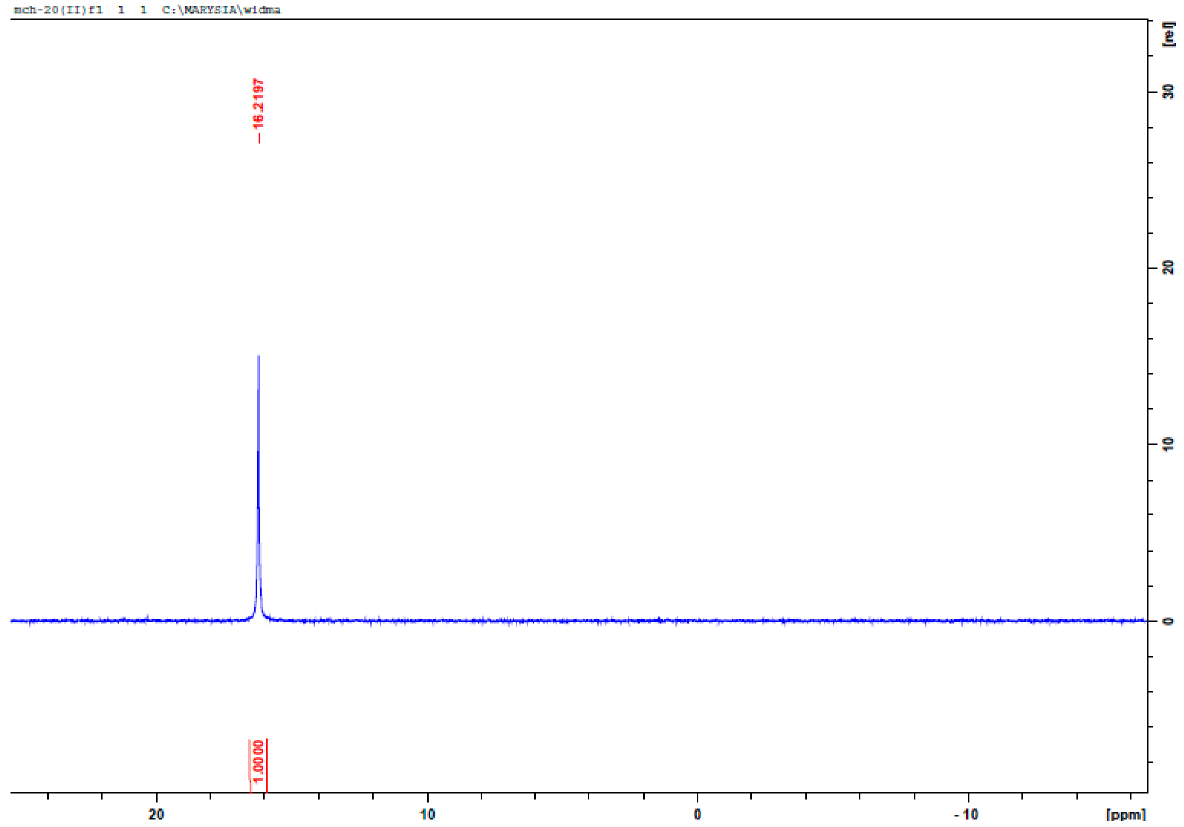

Figure S4. (**b**) Diphenyl *N*-benzylamino(pyren-1-yl)methylphosphonate (**3d**). ^{31}P-NMR.

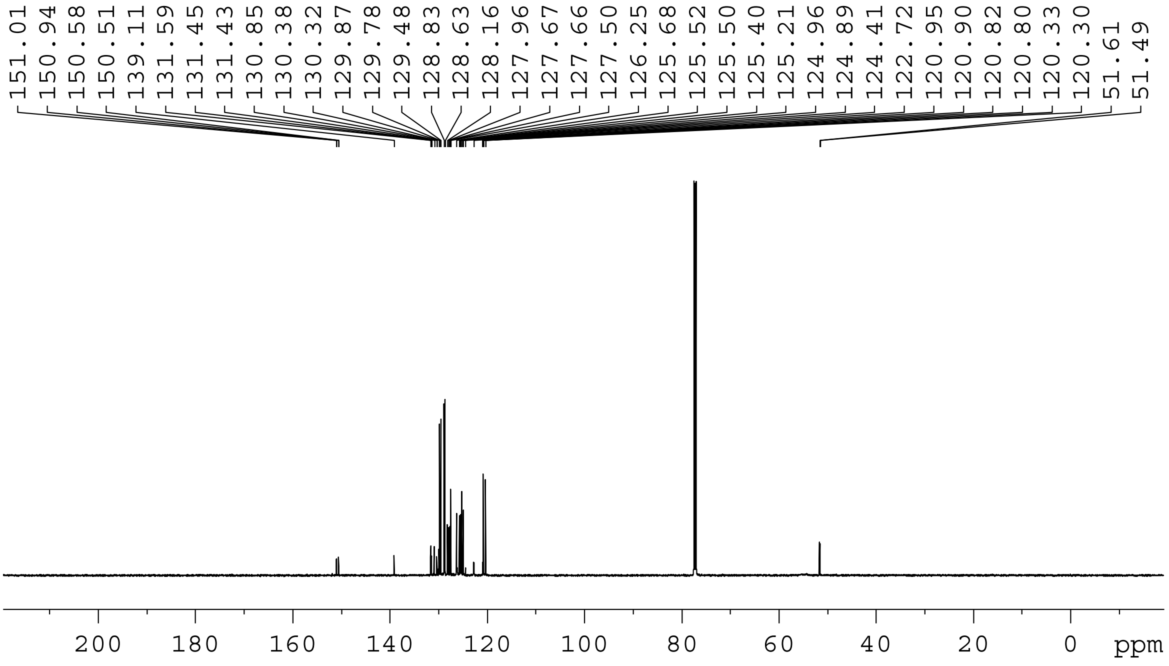

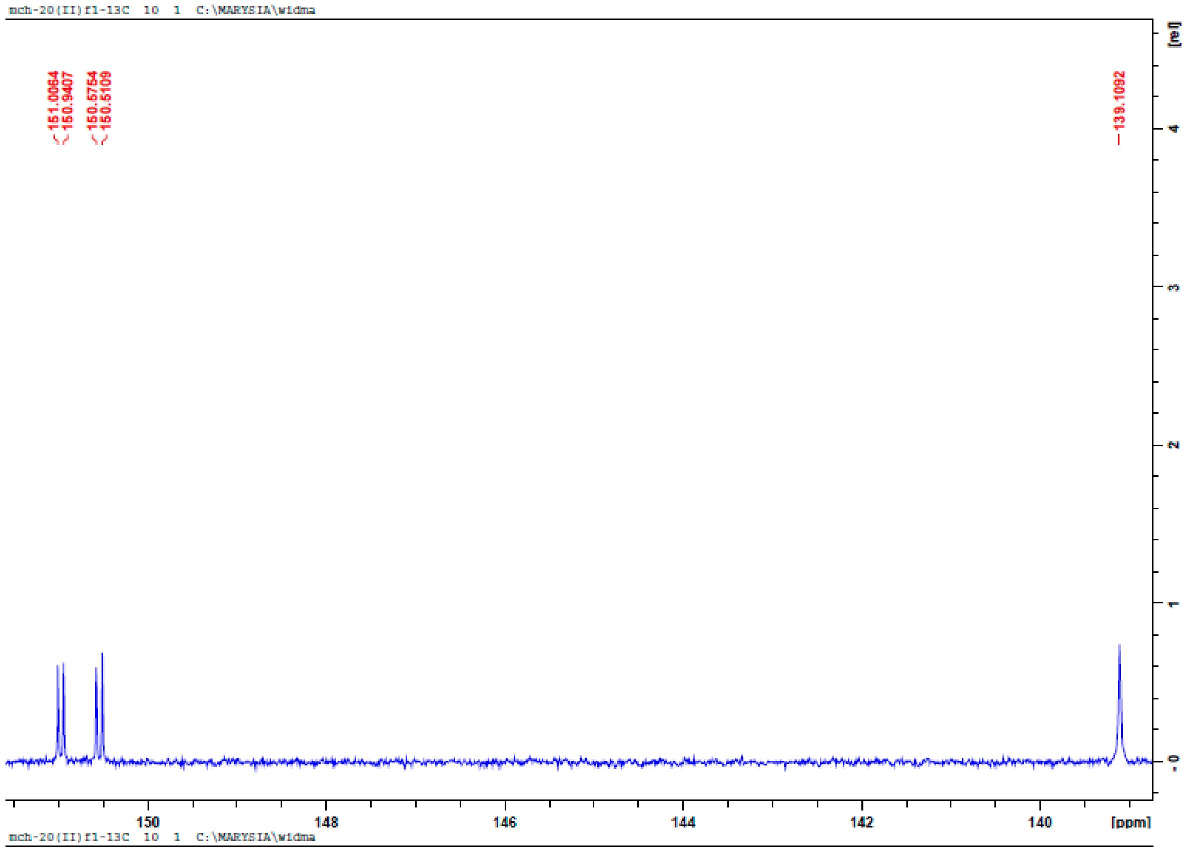
mch-20(II)f1-13C 10 1 C:\MARYSIA\widma
151.0064
150.9407
150.5754
150.5109
139.1092
150
148
146
144
142
140
[ppm]
[rel]

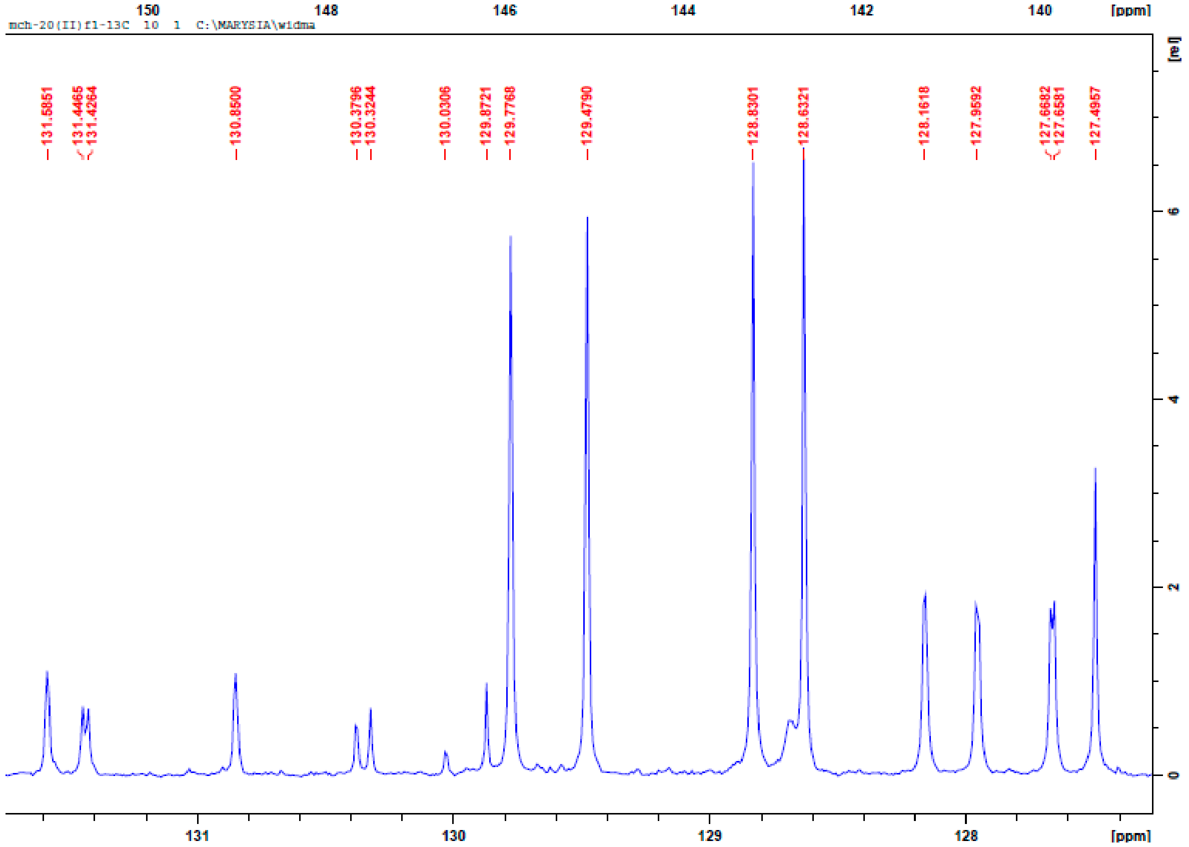
mch-20(II)f1-13C 10 1 C:\MARYSIA\widma
131.5851
131.4465
131.4264
130.8500
130.3796
130.3244
130.0306
129.8721
129.7768
129.4790
128.8301
128.6321
128.1618
127.9592
127.6682
127.6581
127.4957
131
130
129
128
[ppm]
[rel]

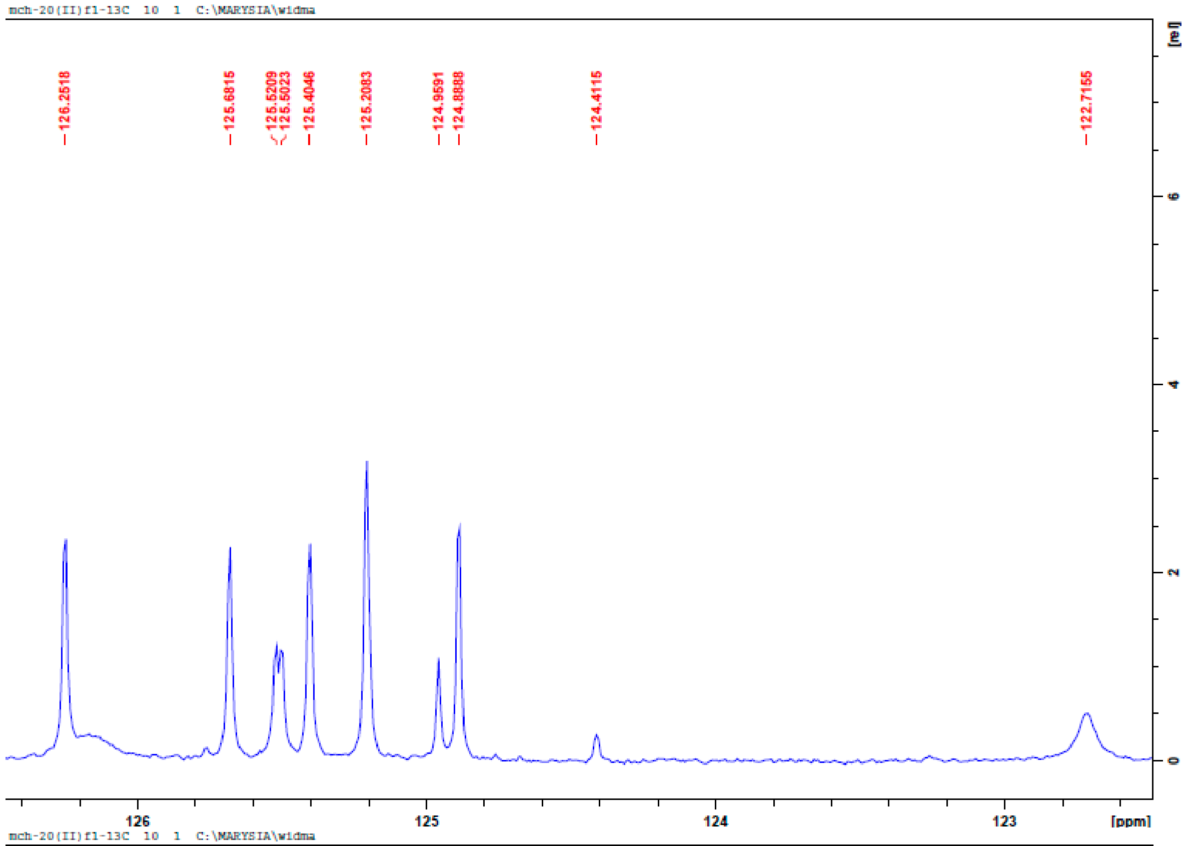
mch-20(II)f1-13C 10 1 C:\MARYSIA\widma
126.2518
125.6815
125.5209
125.5023
125.4046
125.2083
124.9591
124.8888
124.4115
122.7155
126
125
124
123
[ppm]
[rel]
0
2
4
6

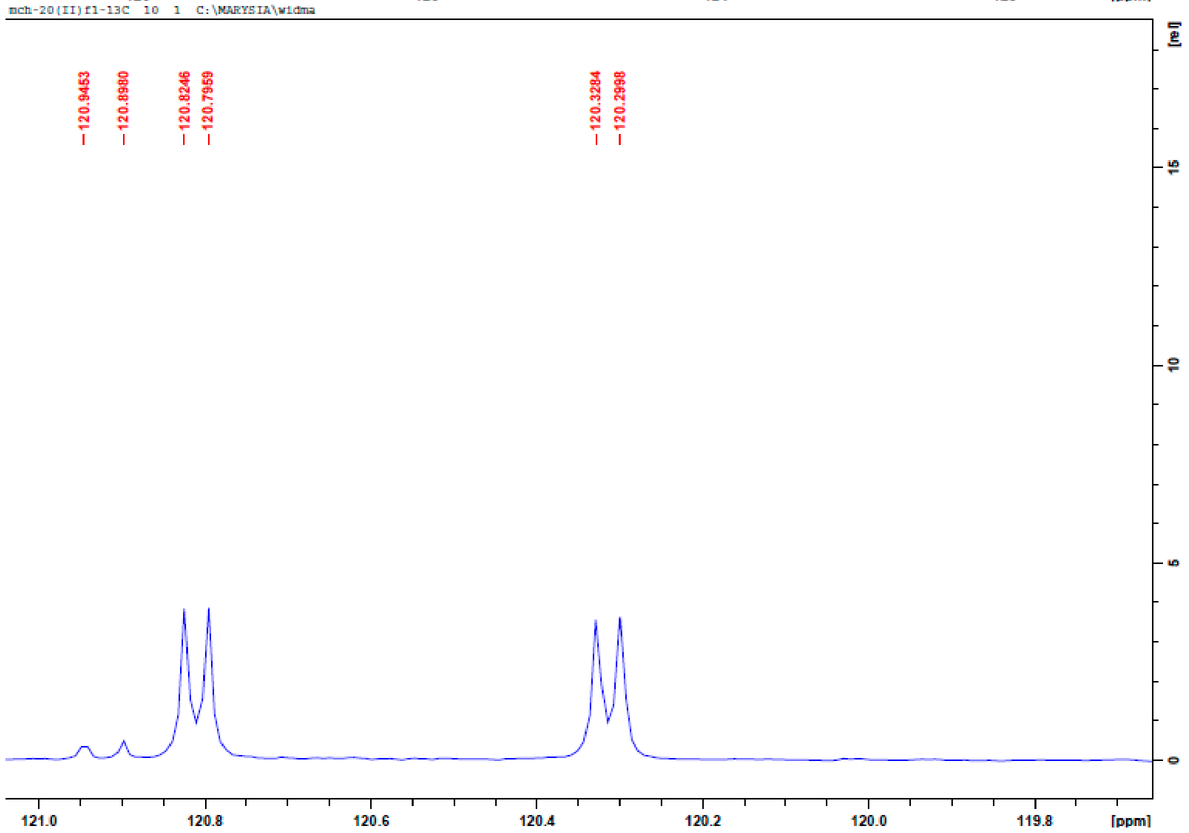
mch-20(II)f1-13C 10 1 C:\MARYSIA\widma
120.9453
120.8980
120.8246
120.7959
120.3284
120.2998
121.0
120.8
120.6
120.4
120.2
120.0
119.8
[ppm]
[rel]
0
5
10
15

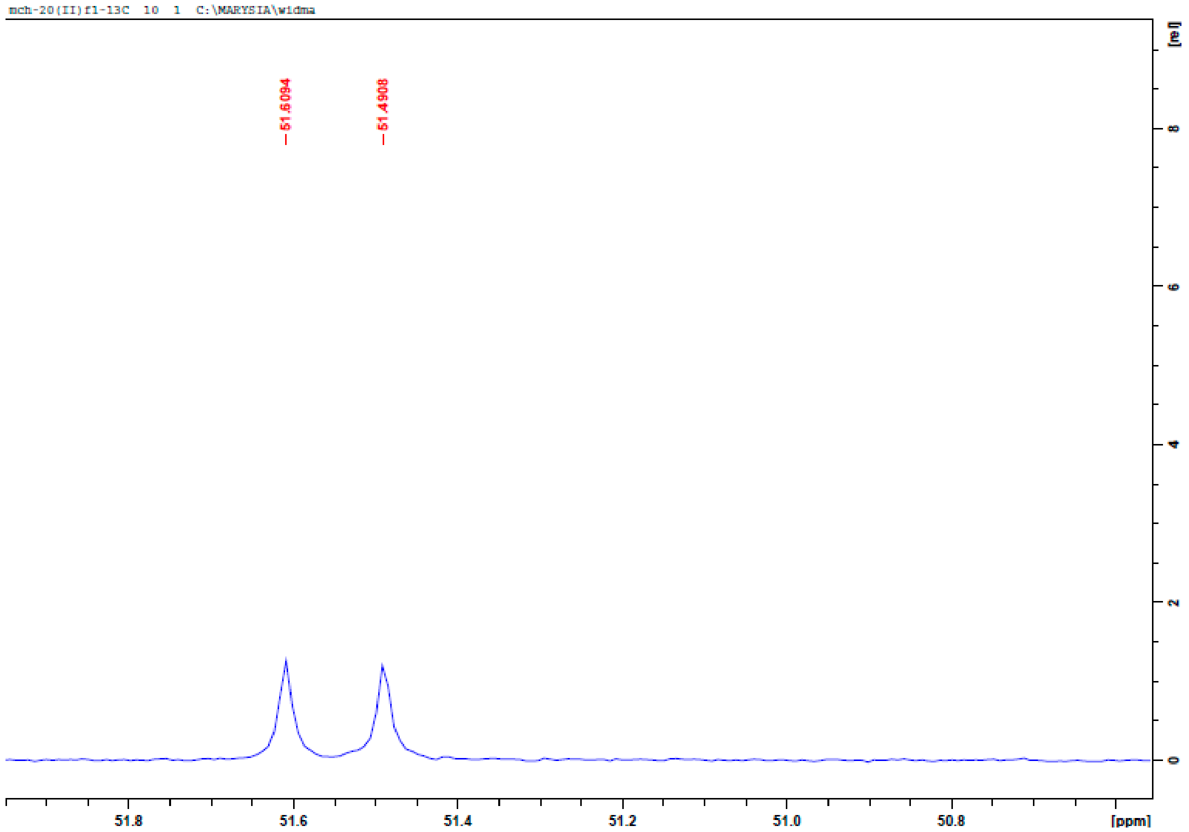

Figure S4. (**c**) Diphenyl *N*-benzylamino(pyren-1-yl)methylphosphonate (**3d**). ^{13}C-NMR—followed by enlarged fragments.

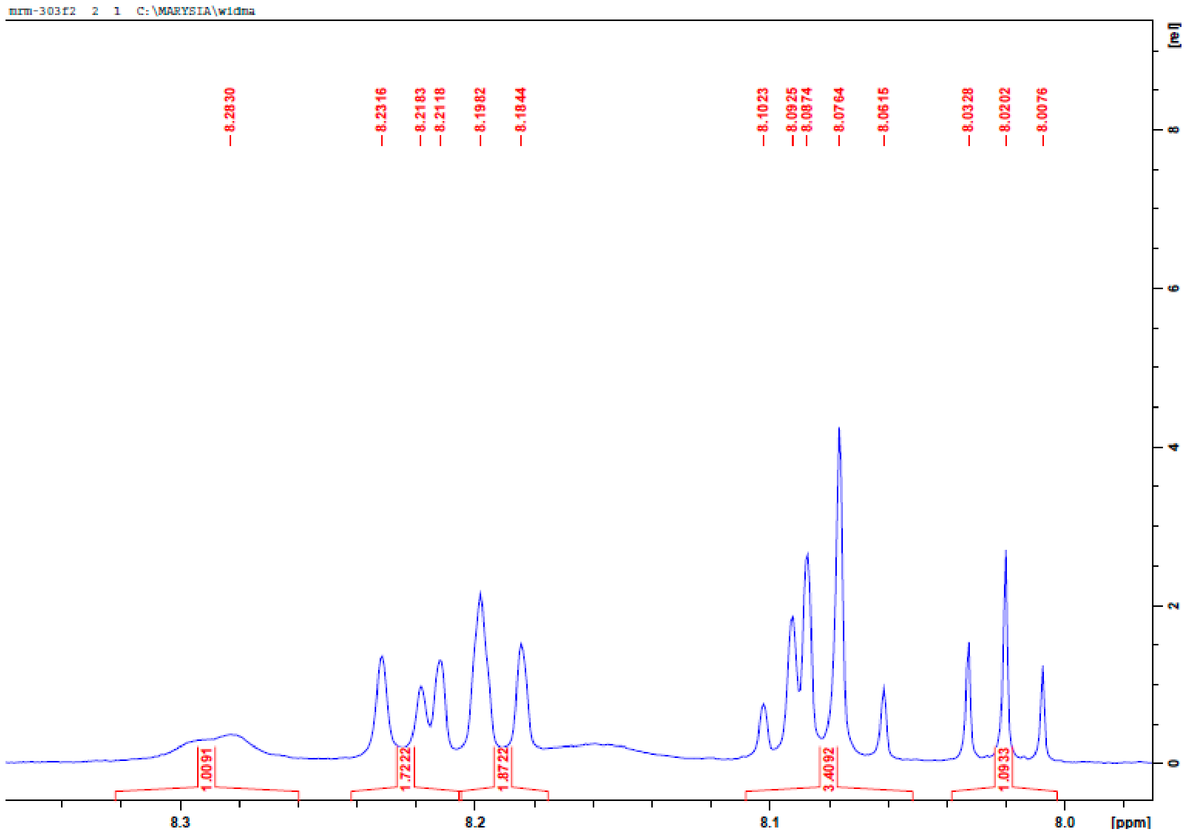

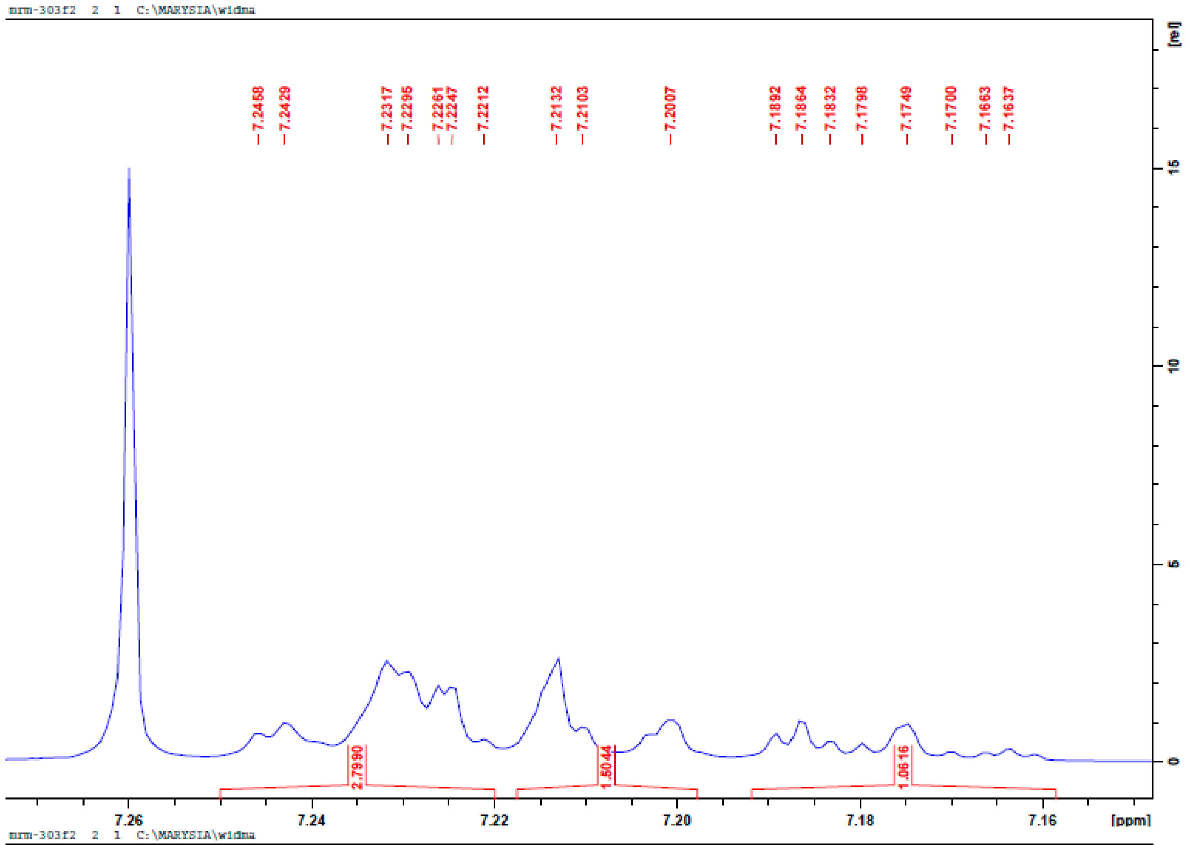
mrm-303f2 2 1 C:\MARYSIA\widma
7.2458
7.2429
7.2317
7.2295
7.2261
7.2247
7.2212
7.2132
7.2103
7.2007
7.1892
7.1864
7.1832
7.1798
7.1749
7.1700
7.1663
7.1637
2.7990
1.5044
1.0616
7.26
7.24
7.22
7.20
7.18
7.16
[ppm]
[rel]
0
5
10
15

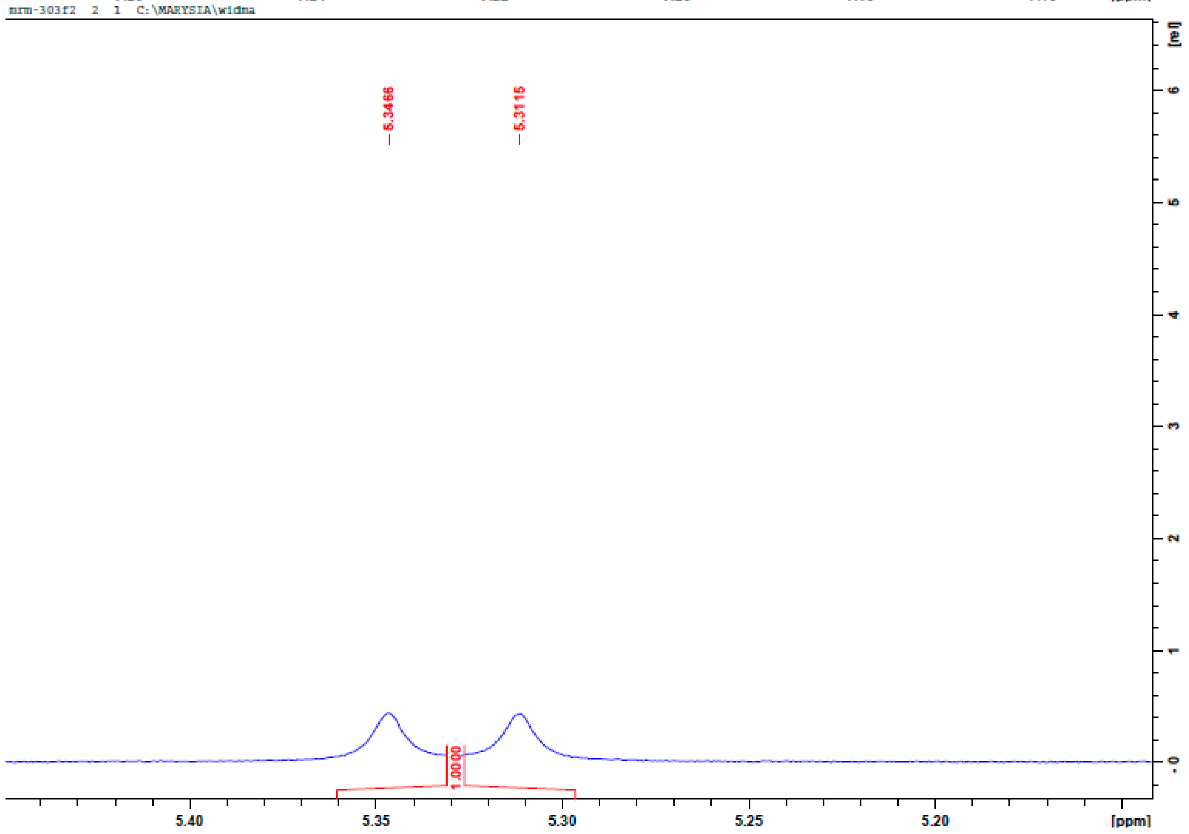
mrm-303f2 2 1 C:\MARYSIA\widma
5.3466
5.3115
1.0000
5.40
5.35
5.30
5.25
5.20
[ppm]
[rel]
0
1
2
3
4
5
6

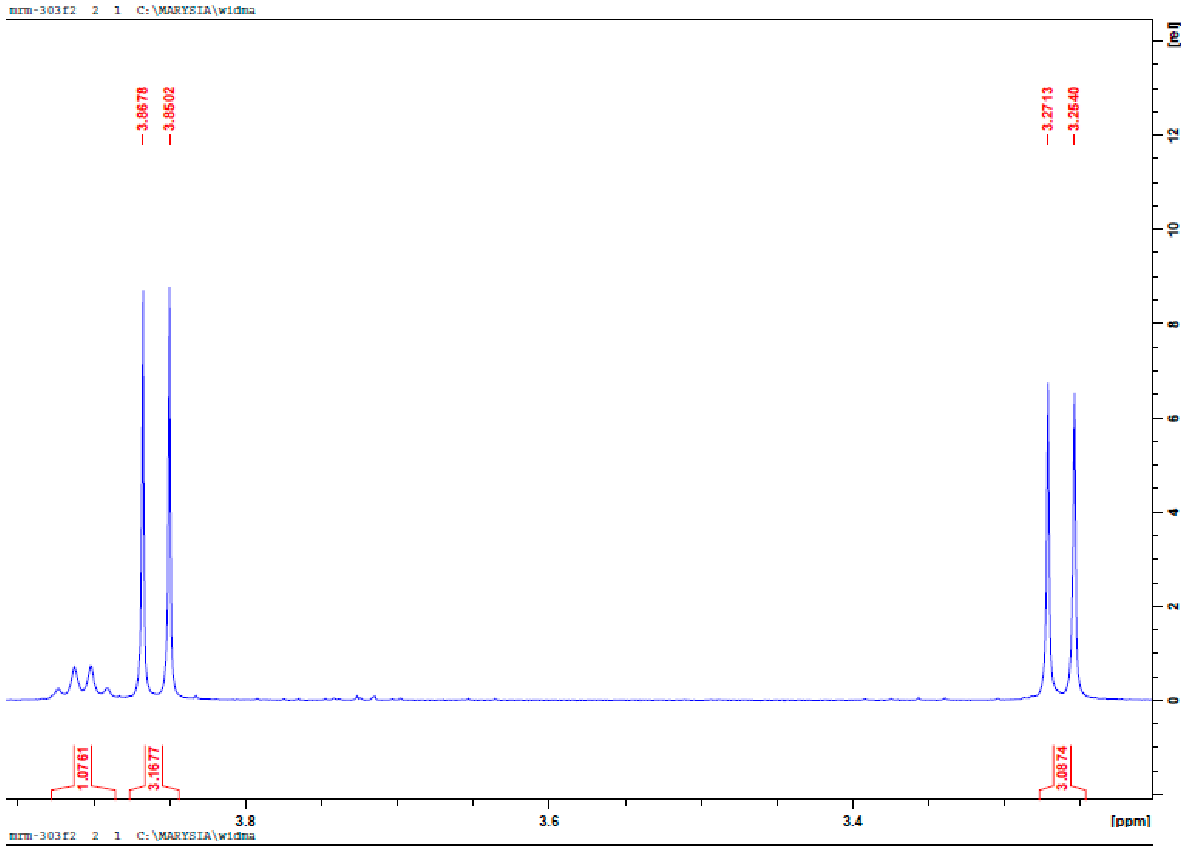

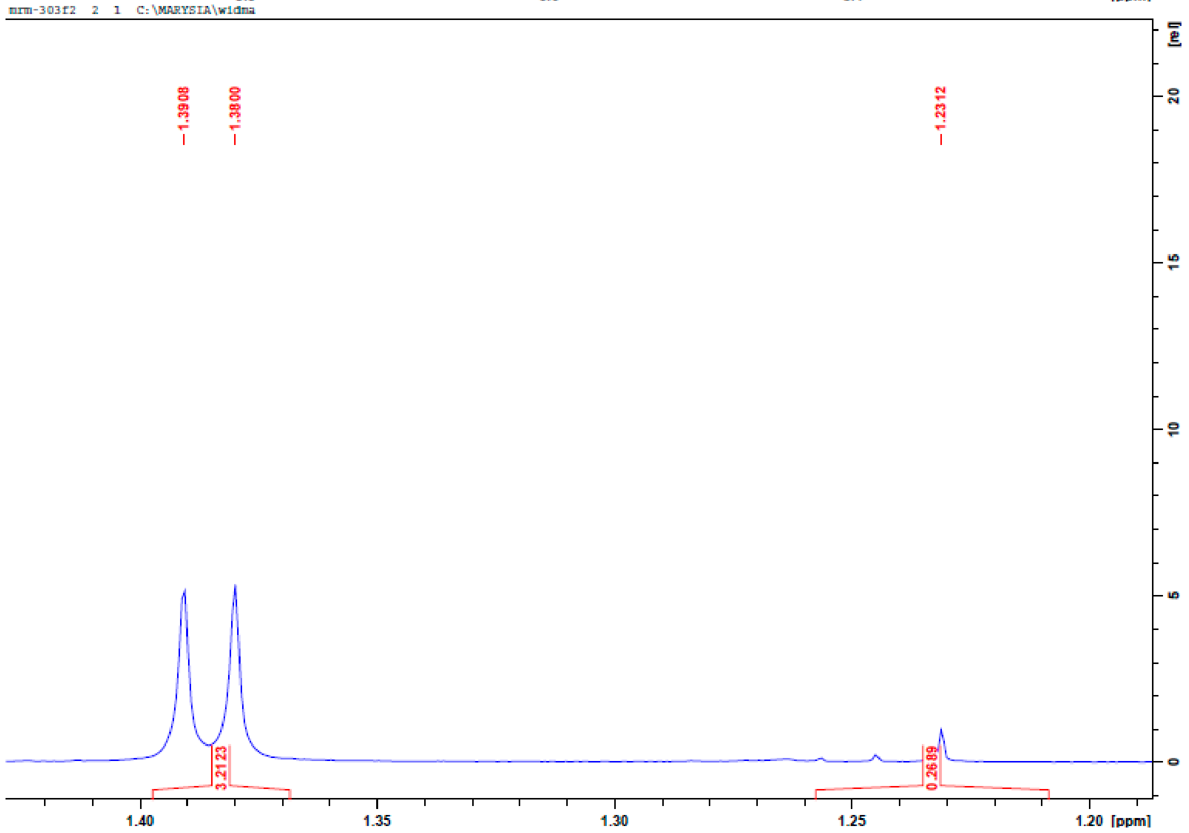

Figure S5. (**a**) Dimethyl *N*-(*R*)-α-methylbenzylamino(pyren-1-yl)methylphosphonate (**4**). Isolated predominant diastereoisomer ^{1}H-NMR—followed by enlarged fragments.

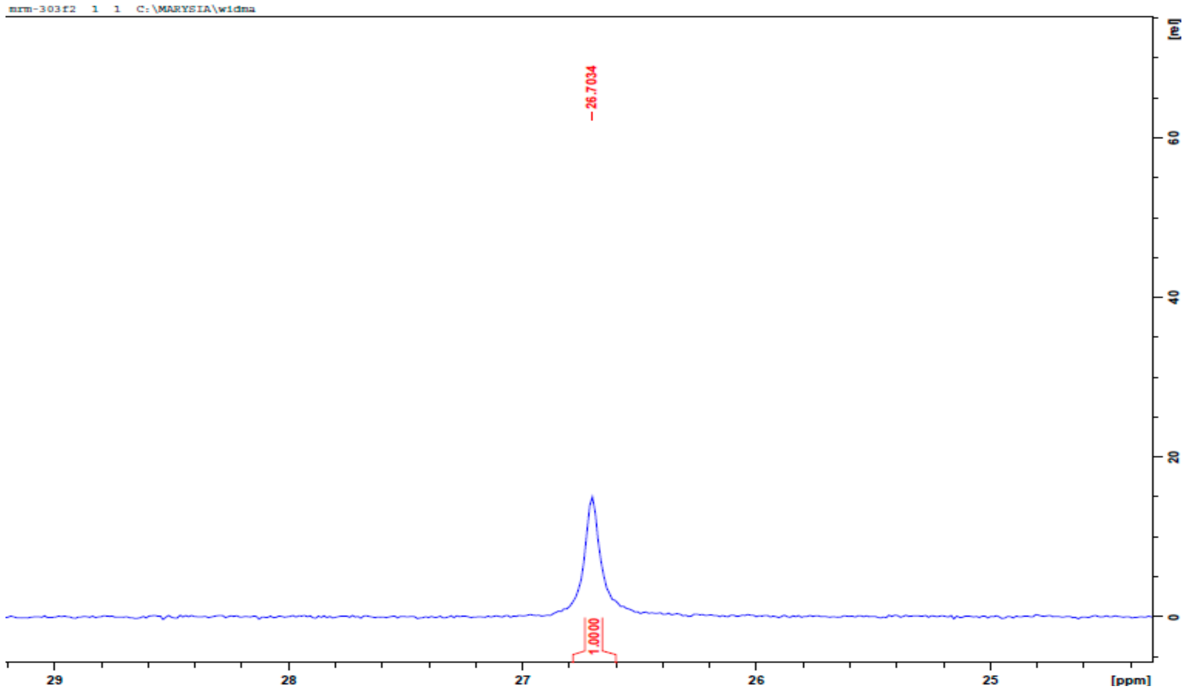

Figure S5. (**b**) Dimethyl *N*-(*R*)-α-methylbenzylamino(pyren-1-yl)methylphosphonate (**4**). Isolated predominant diastereoisomer ^{31}P-NMR.

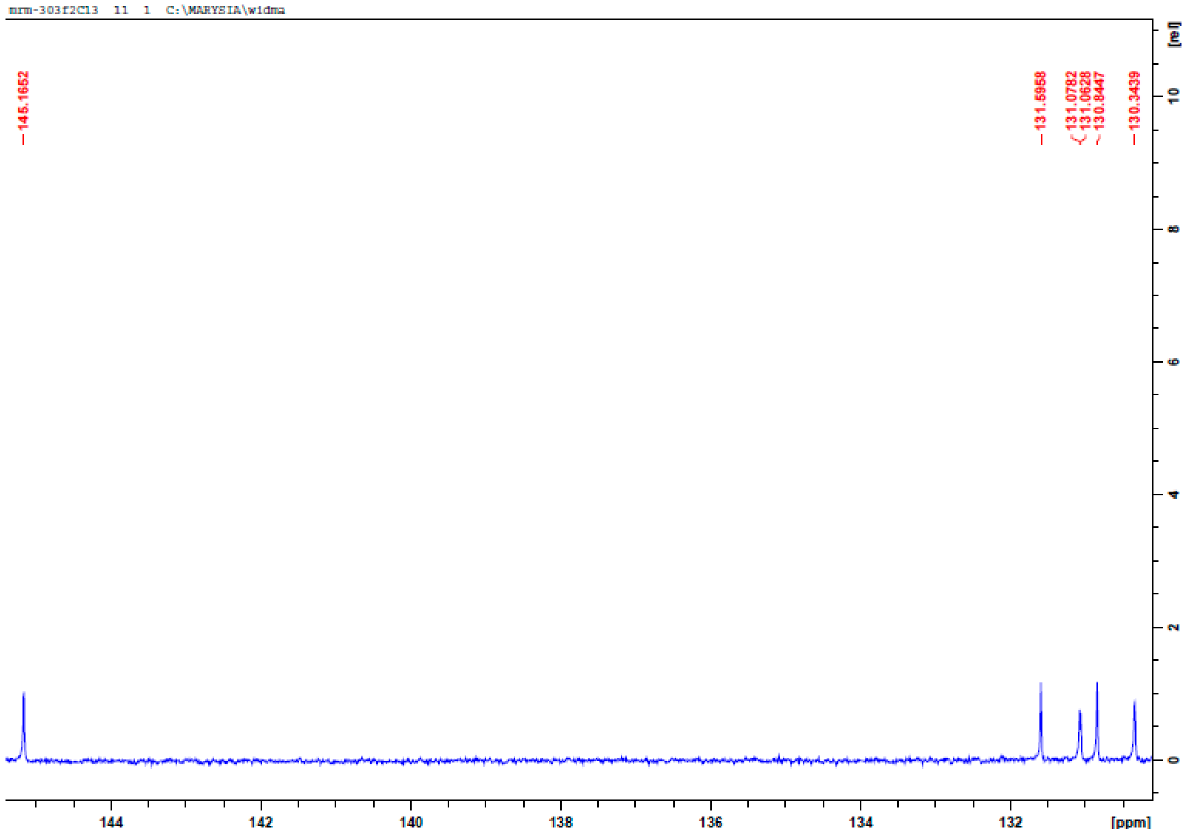

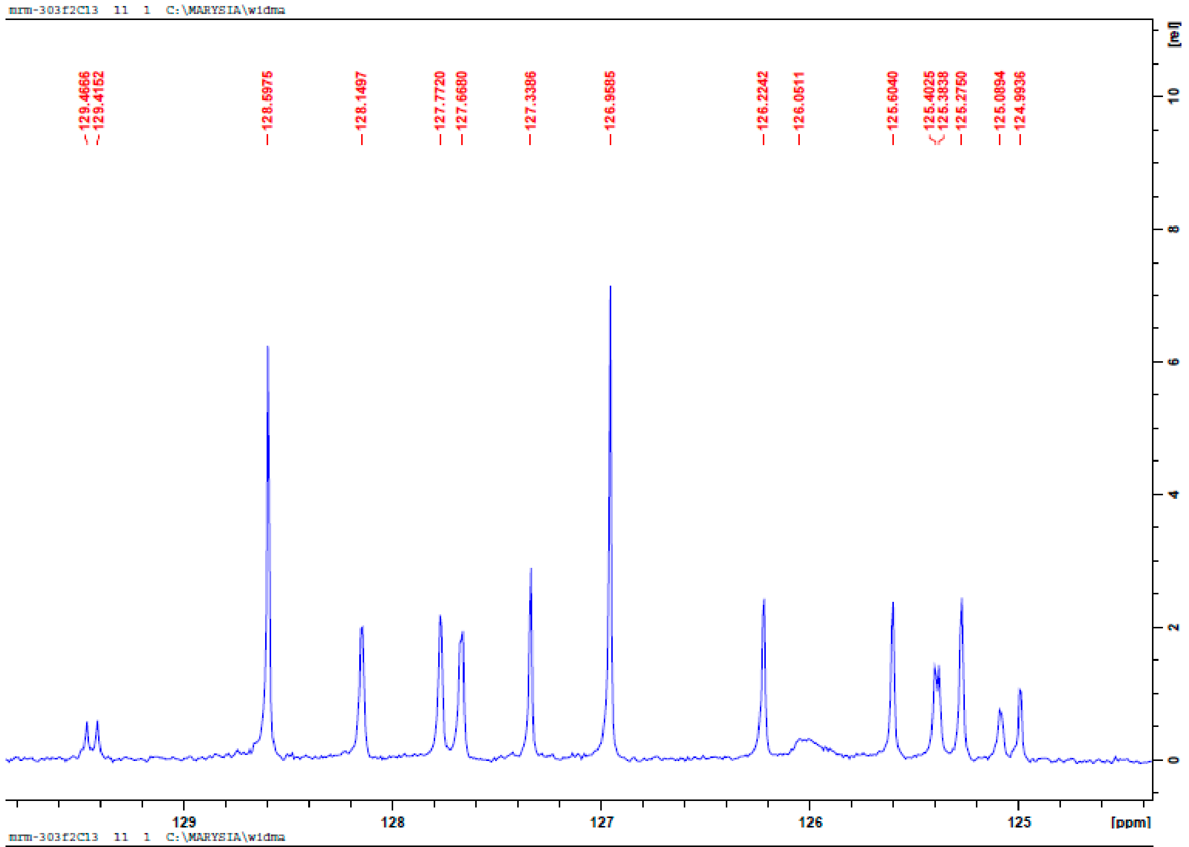
mrm-303f2C13 11 1 C:\MARYSIA\widma
129.4666
129.4152
128.5975
128.1497
127.7720
127.6680
127.3386
126.9585
126.2242
126.0511
125.6040
125.4025
125.3838
125.2750
125.0894
124.9936
[rel]
129
128
127
126
125
[ppm]

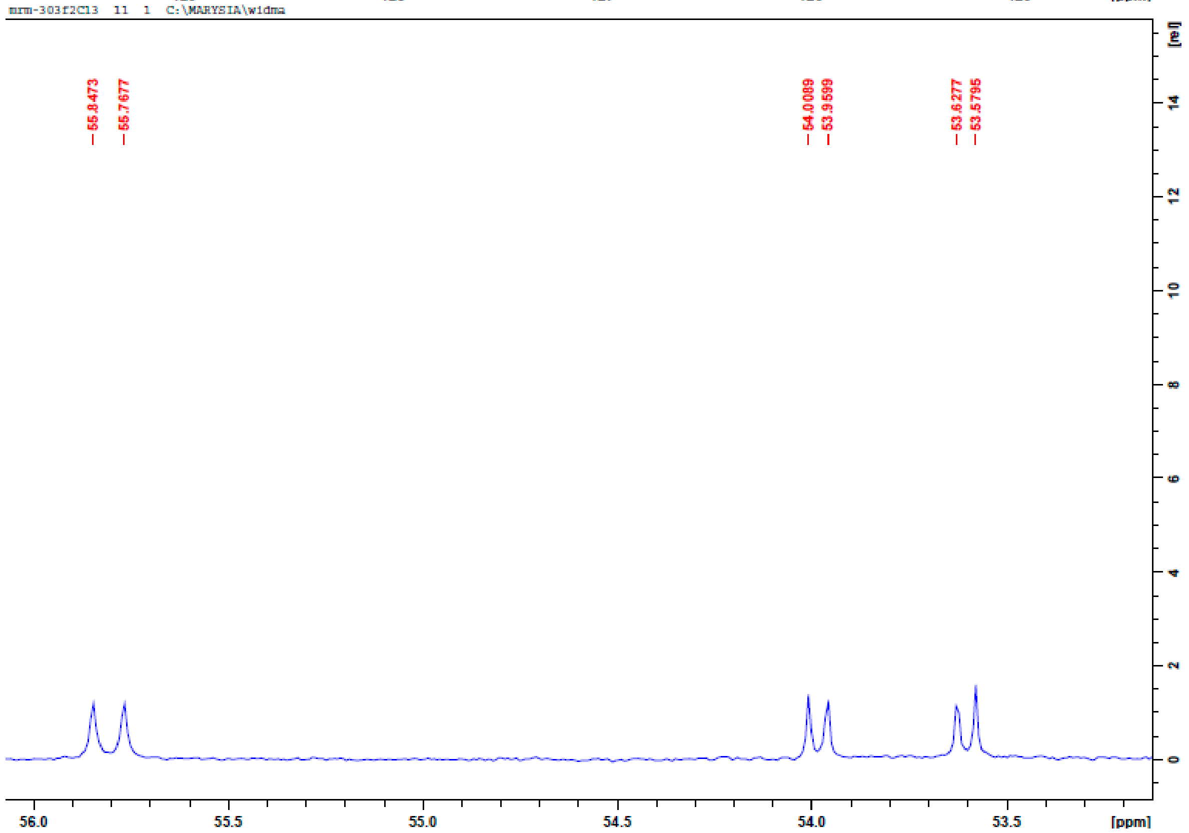
mrm-303f2C13 11 1 C:\MARYSIA\widma
55.8473
55.7677
54.0089
53.9599
53.6277
53.5795
[rel]
56.0
55.5
55.0
54.5
54.0
53.5
[ppm]

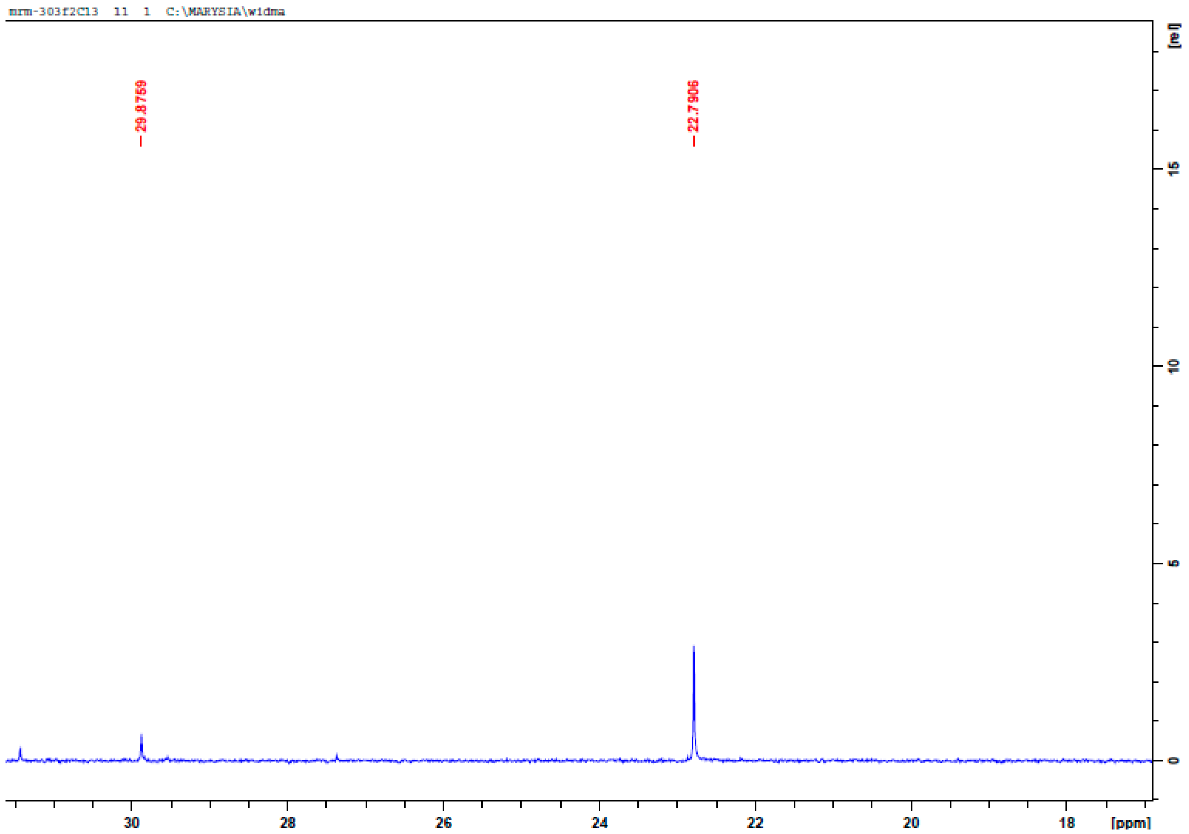

Figure S5. (**c**) Dimethyl *N*-(*R*)-α-methylbenzylamino(pyren-1-yl)methylphosphonate (**4**). Isolated predominant diastereoisomer ^{13}C-NMR—followed by enlarged fragments.

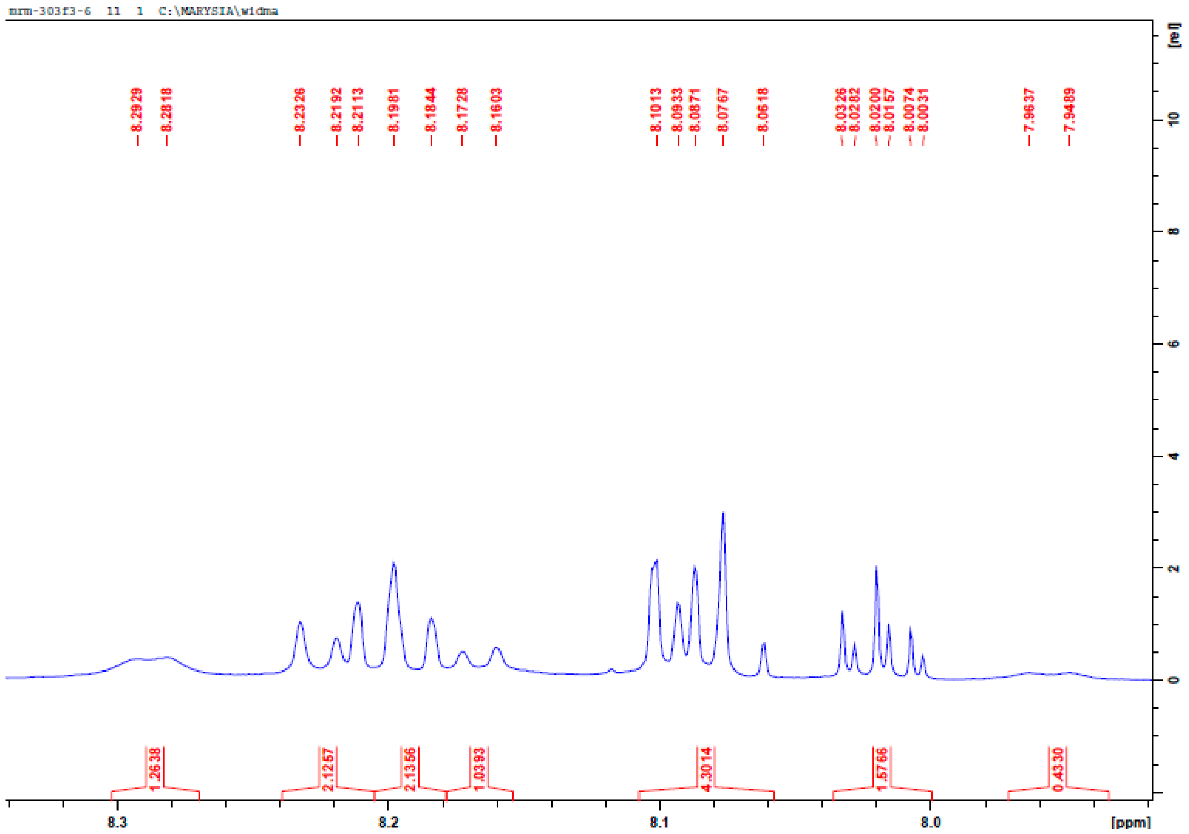

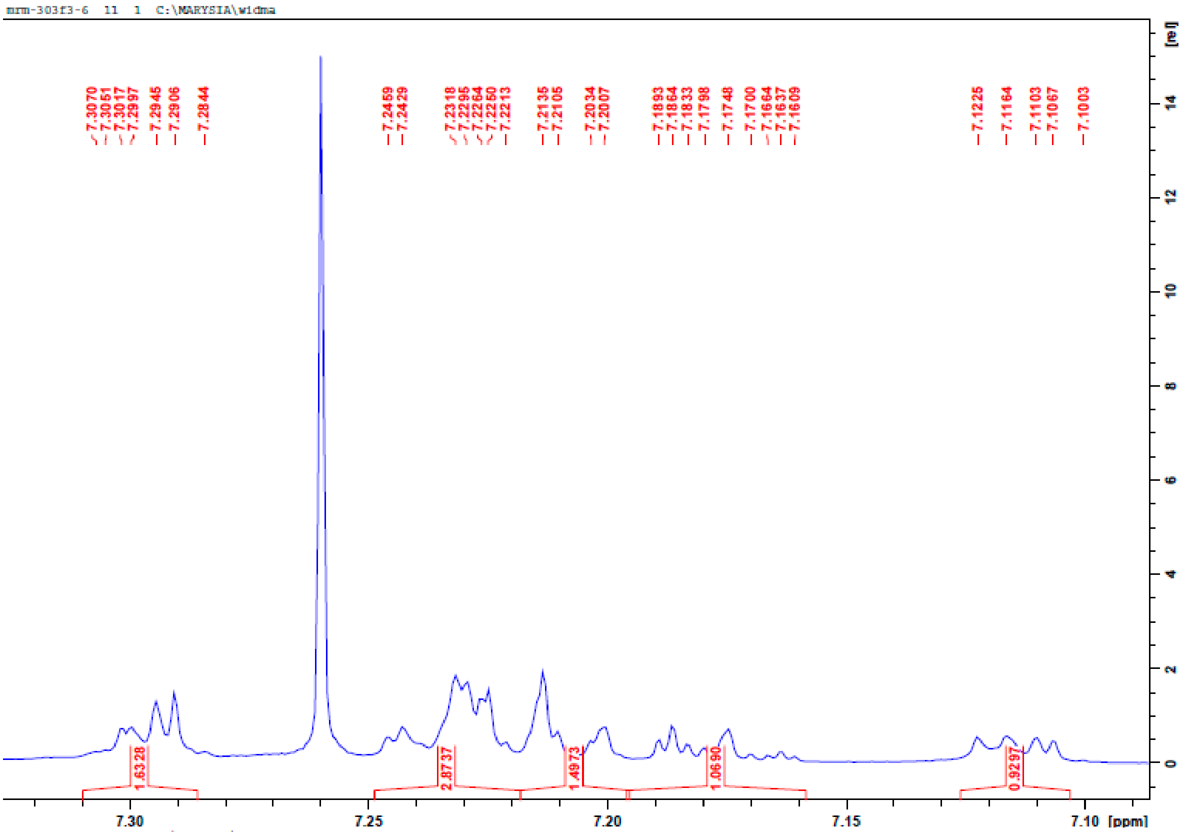
mrm-303f3-6 11 1 C:\MARYSIA\widma
7.3070
7.3051
7.3017
7.2997
7.2945
7.2906
7.2844
7.2459
7.2429
7.2318
7.2295
7.2264
7.2250
7.2213
7.2135
7.2105
7.2034
7.2007
7.1893
7.1864
7.1833
7.1798
7.1748
7.1700
7.1664
7.1637
7.1609
7.1225
7.1164
7.1103
7.1067
7.1003
1.6328
2.8737
1.4973
1.0690
0.9297
7.30
7.25
7.20
7.15
7.10
[ppm]
[rel]
14
12
10
8
6
4
2
0

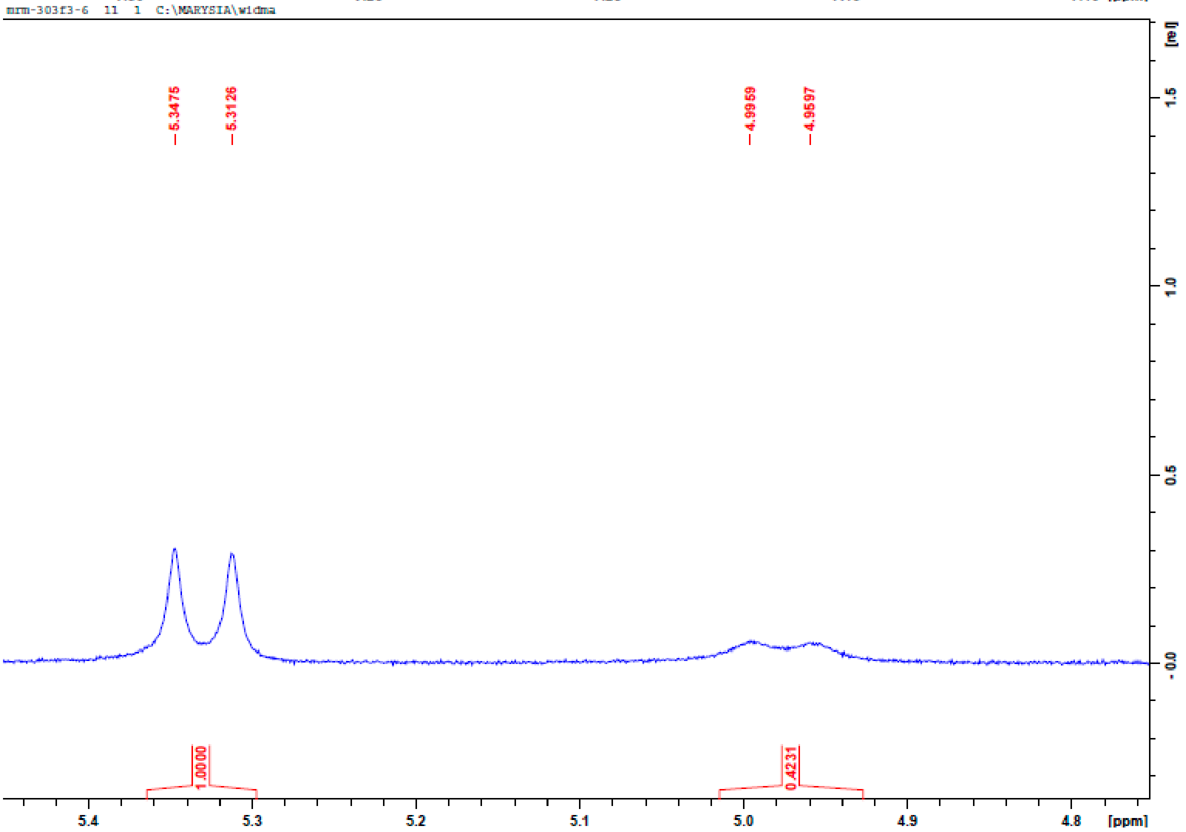
mrm-303f3-6 11 1 C:\MARYSIA\widma
5.3475
5.3126
4.9959
4.9597
1.0000
0.4231
5.4
5.3
5.2
5.1
5.0
4.9
4.8
[ppm]
[rel]
1.5
1.0
0.5
0.0

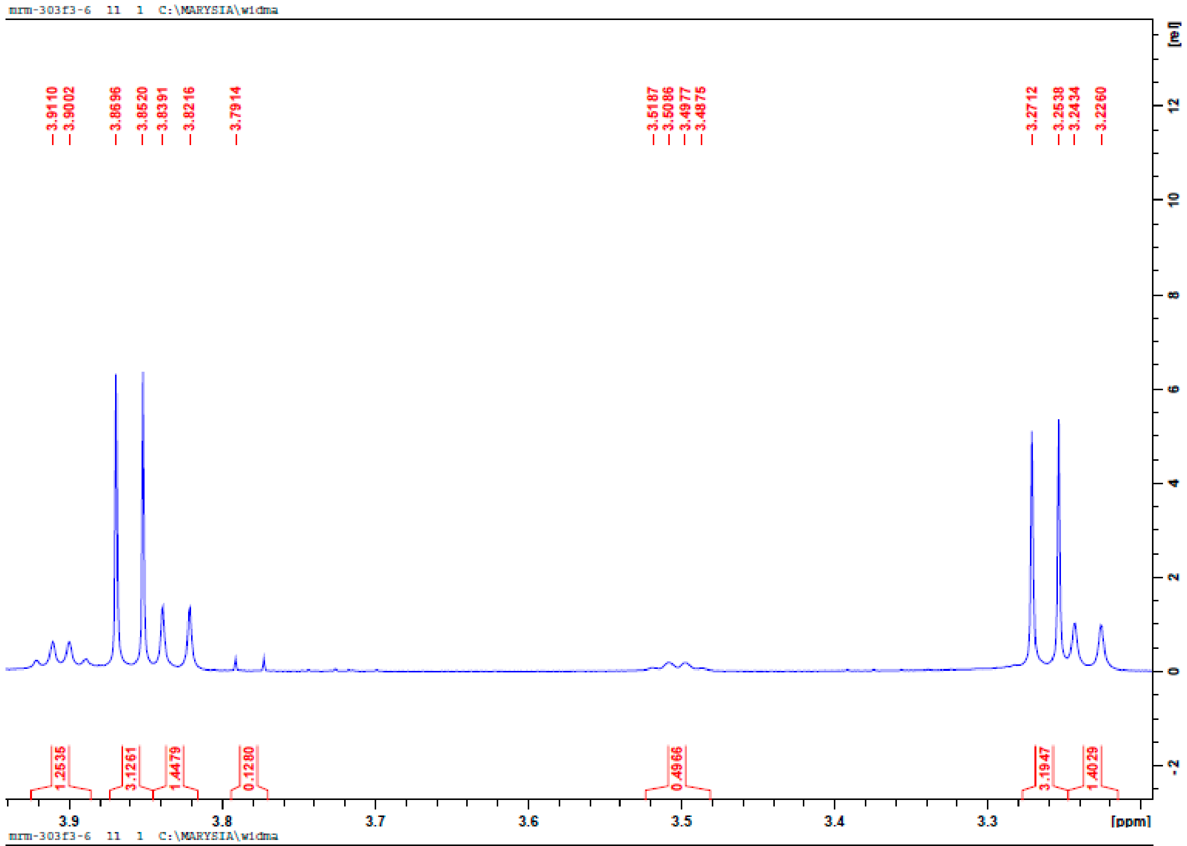

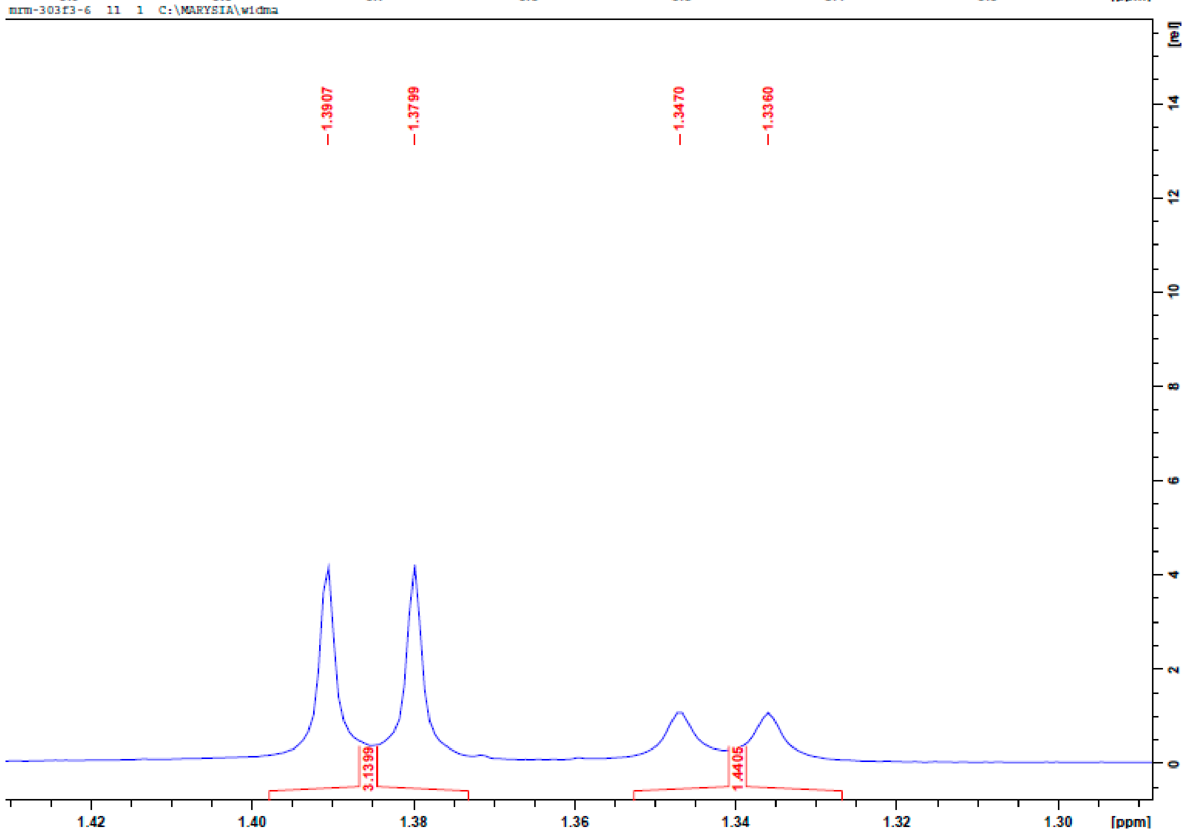

Figure S6. (**a**) Dimethyl *N*-(*R*)-α-methylbenzylamino(pyren-1-yl)methylphosphonate (**4**). Mixture of diastereoisomers ^{1}H-NMR—followed by enlarged fragments.

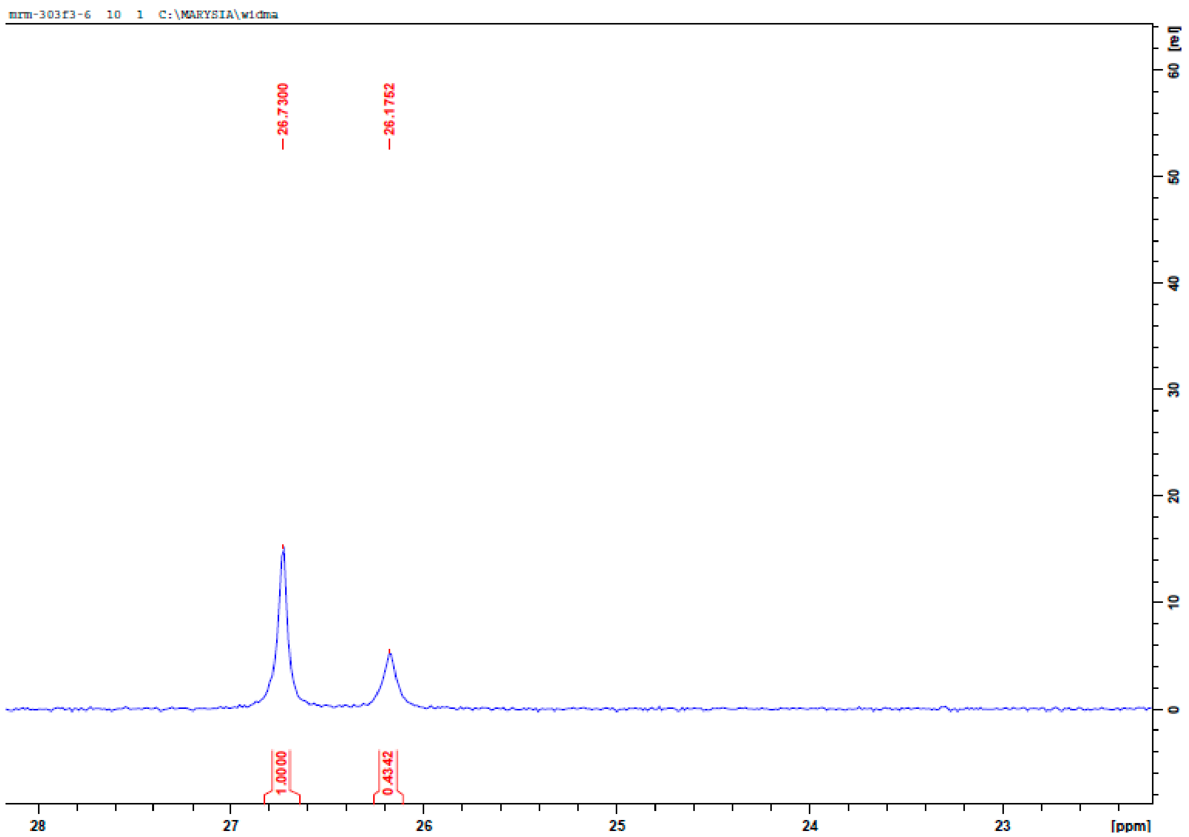

Figure S6. (**b**) Dimethyl *N*-(*R*)-α-methylbenzylamino(pyren-1-yl)methylphosphonate (**4**). Mixture of diastereoisomers ^{31}P-NMR.

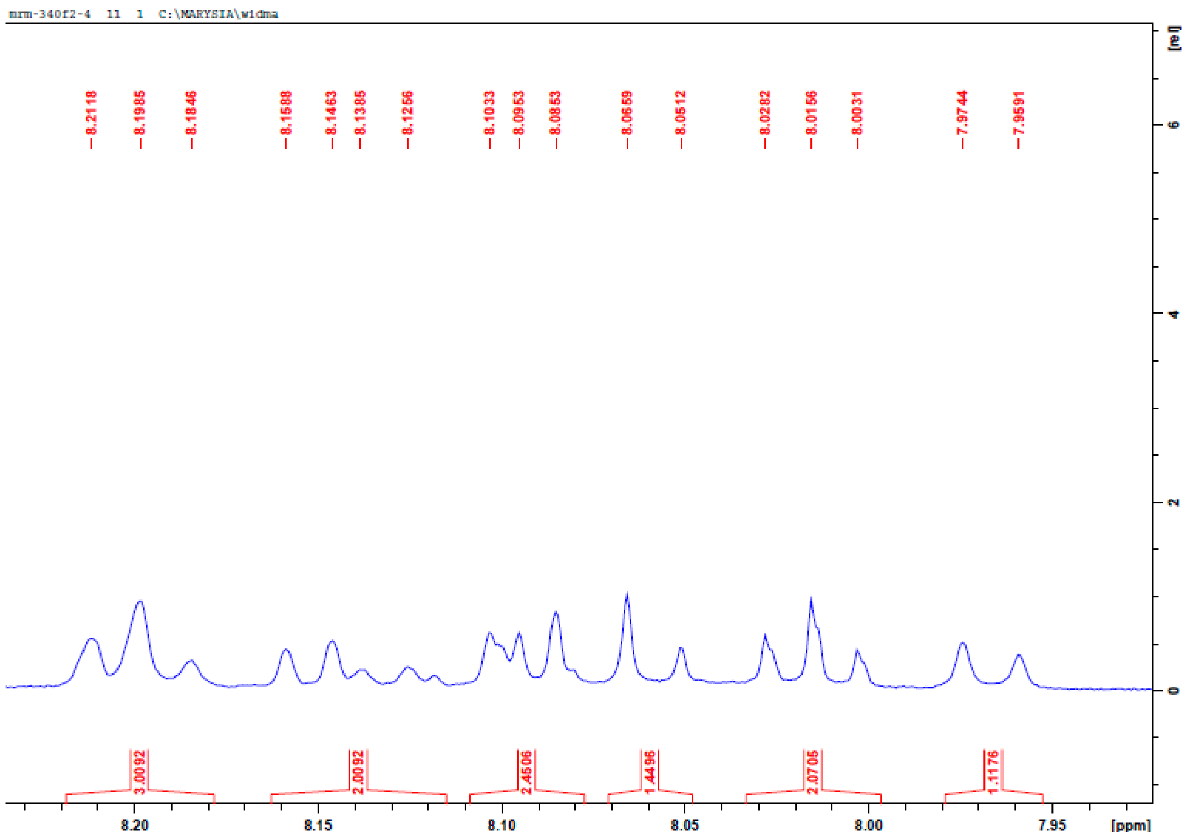

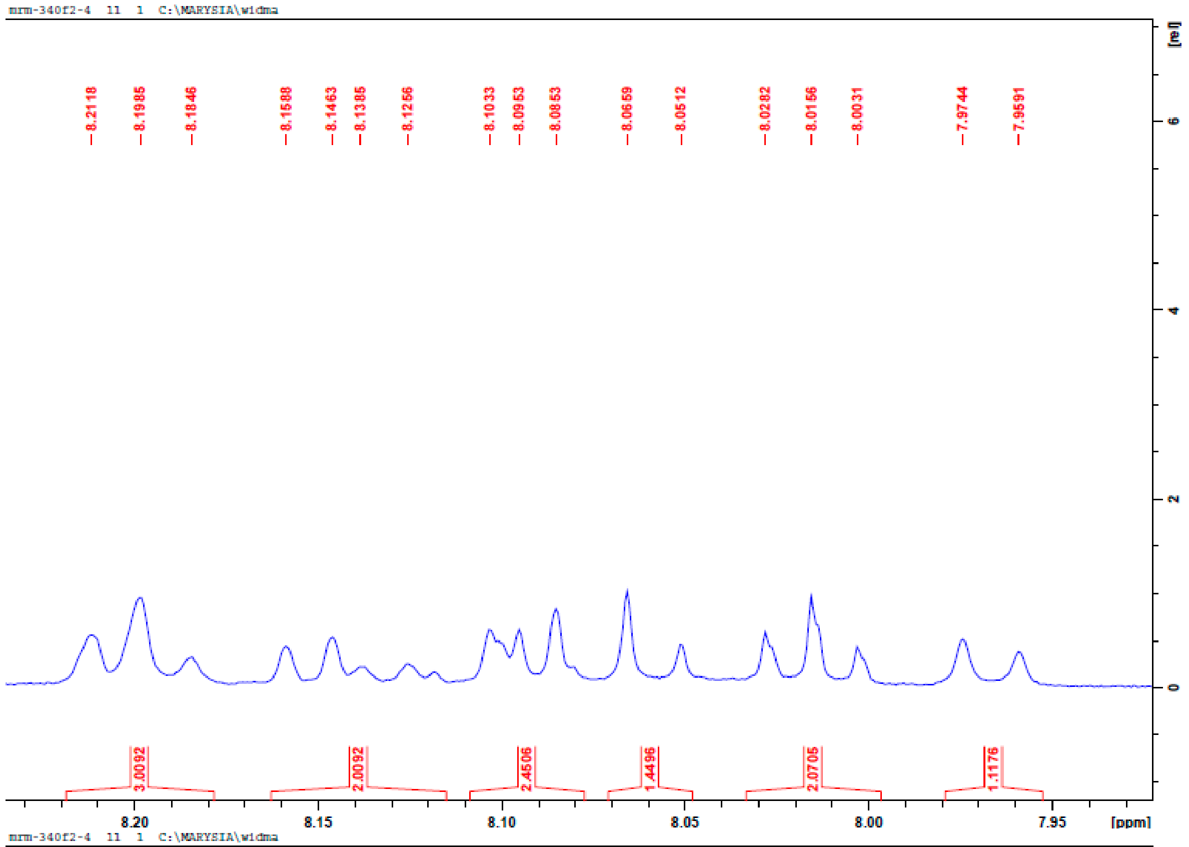

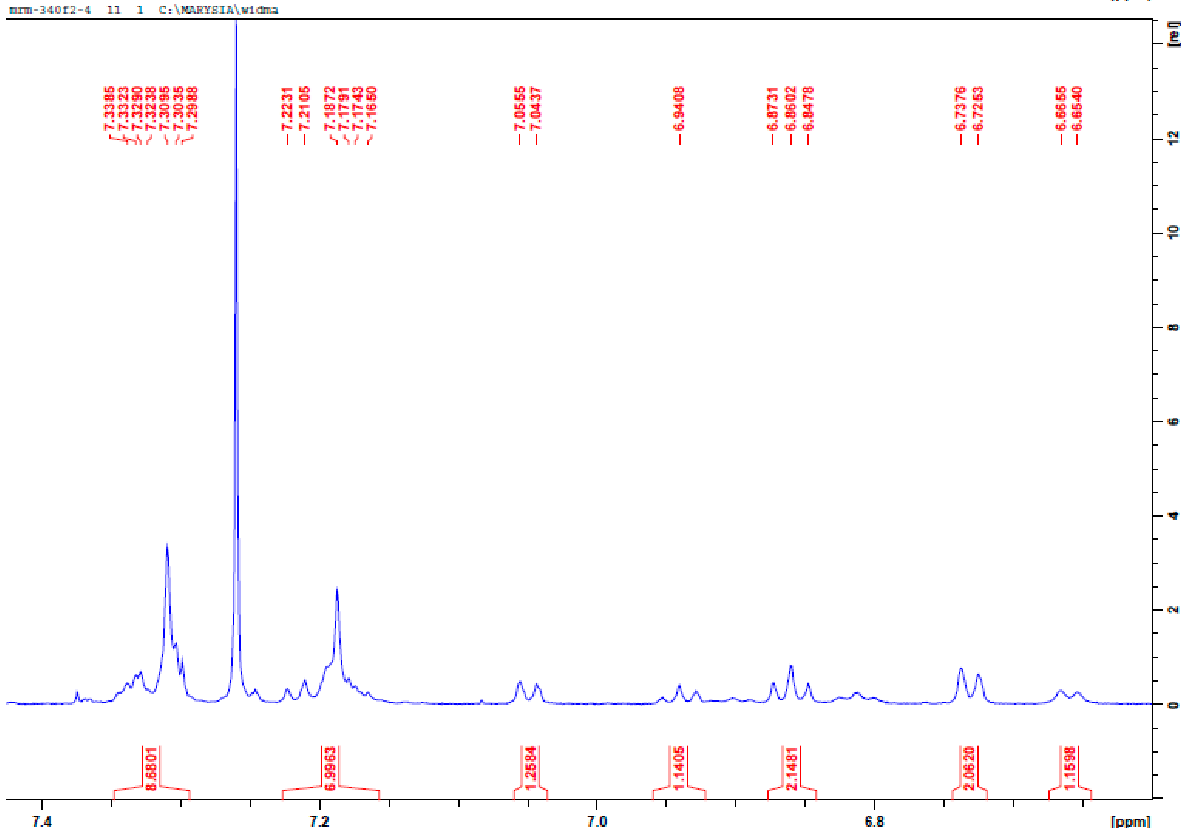

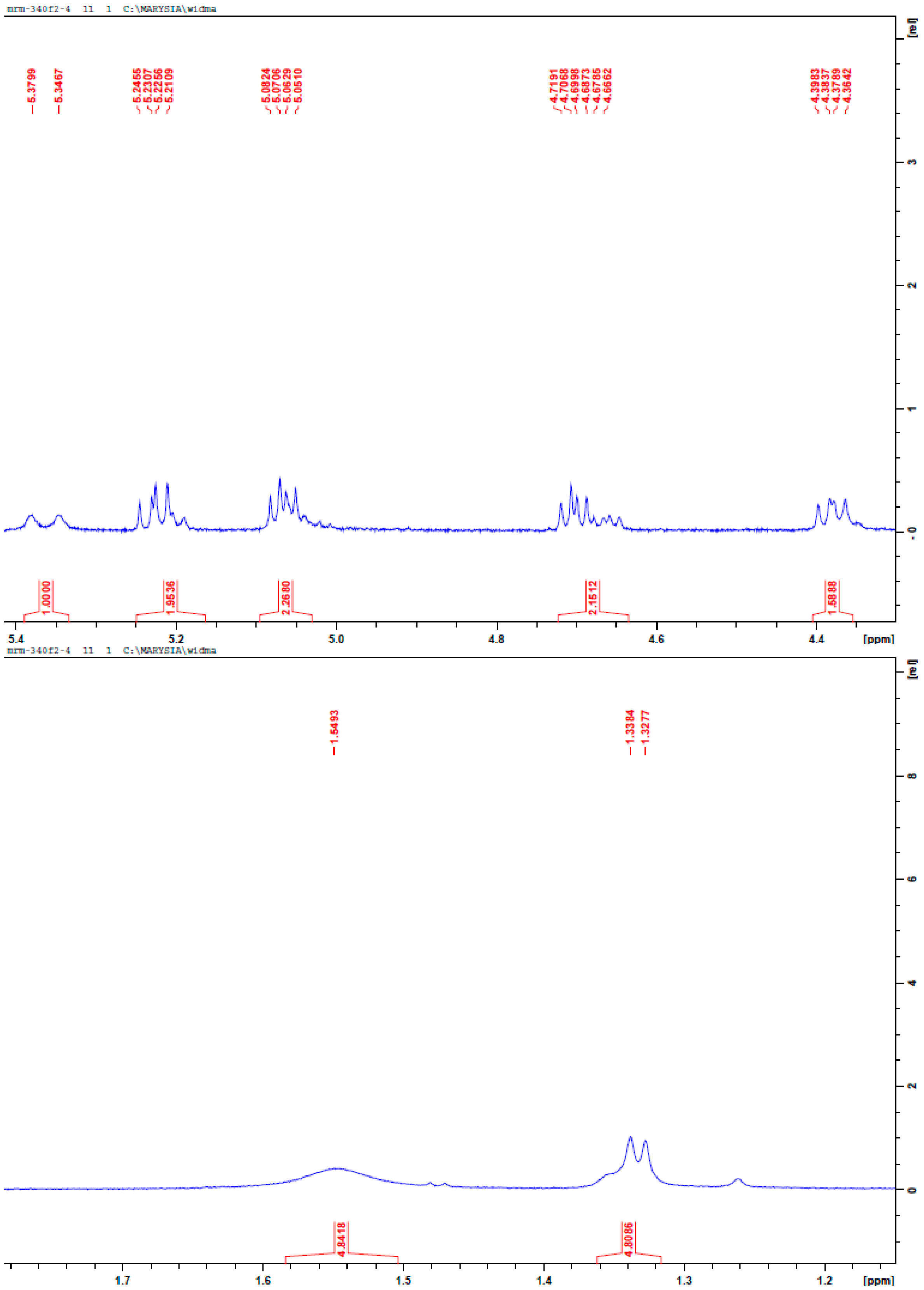

Figure S7. (**a**) Dibenzyl *N*-(*R*)-*α*-methylbenzylamino(pyren-1-yl)methylphosphonate (**5**). Mixture of diastereoisomers ^{1}H-NMR—followed by enlarged fragments.

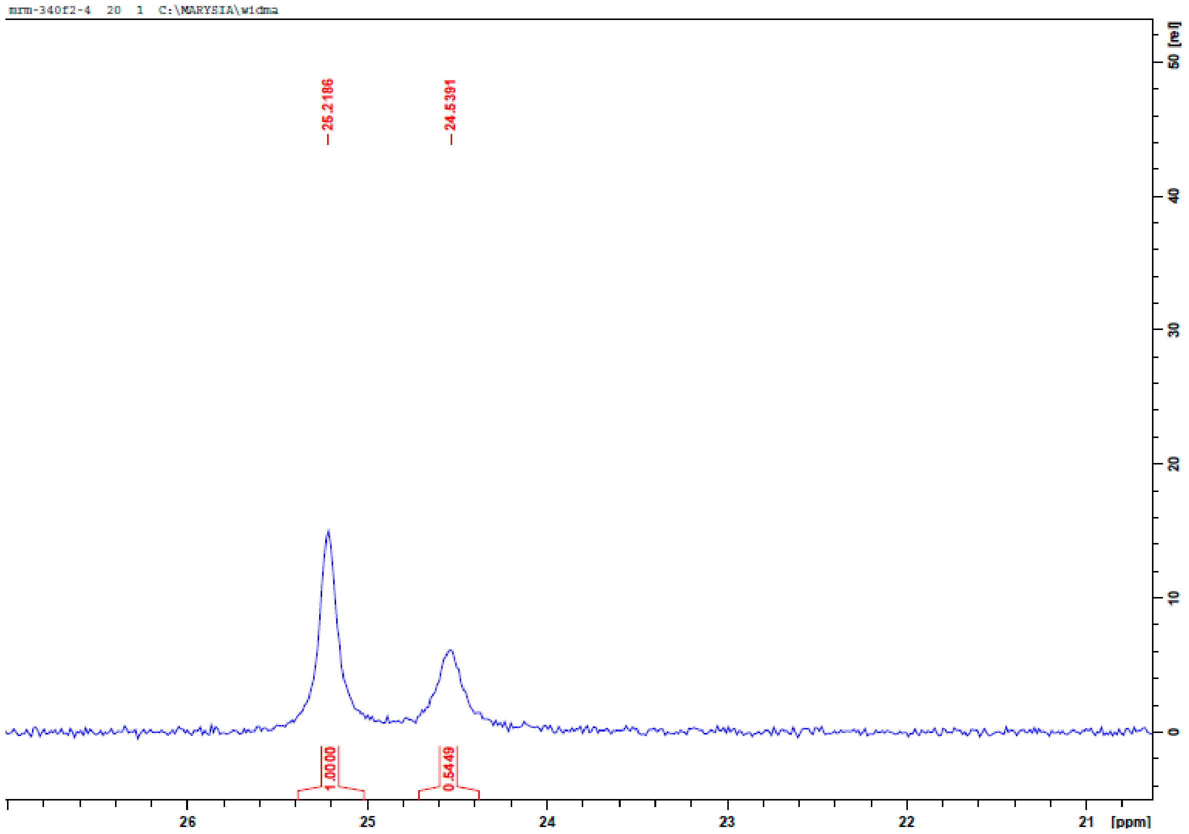

Figure S7. (**b**) Dibenzyl *N*-(*R*)-α-methylbenzylamino(pyren-1-yl)methylphosphonate (**5**). Mixture of diastereoisomers ^{31}P-NMR.

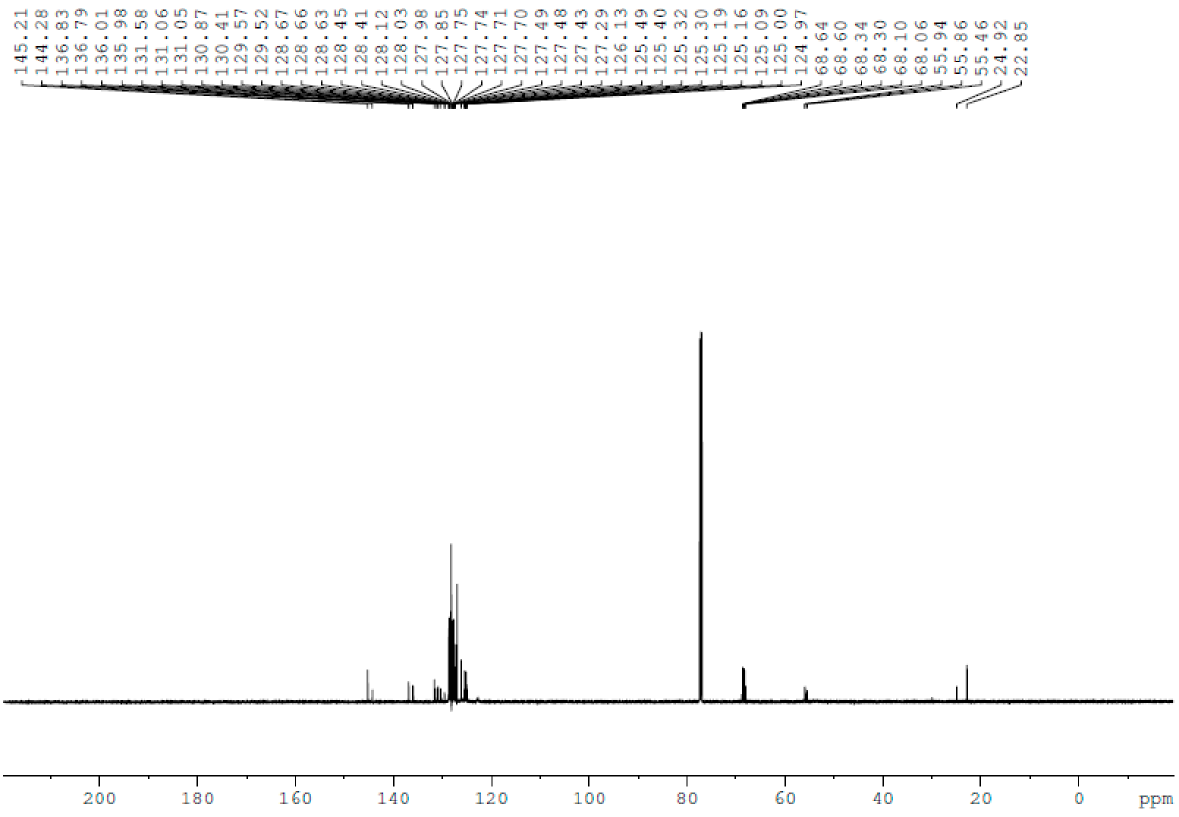

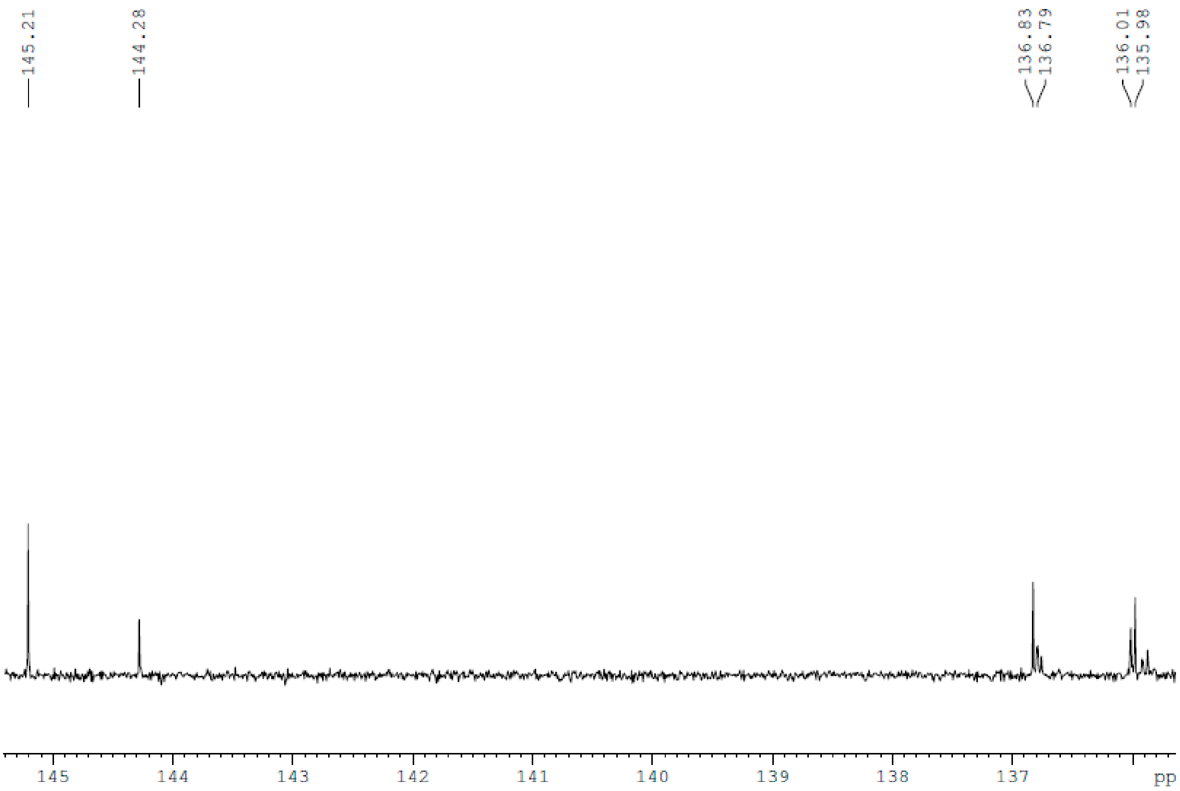
145.21
144.28
136.83
136.79
136.01
135.98
145
144
143
142
141
140
139
138
137
pp

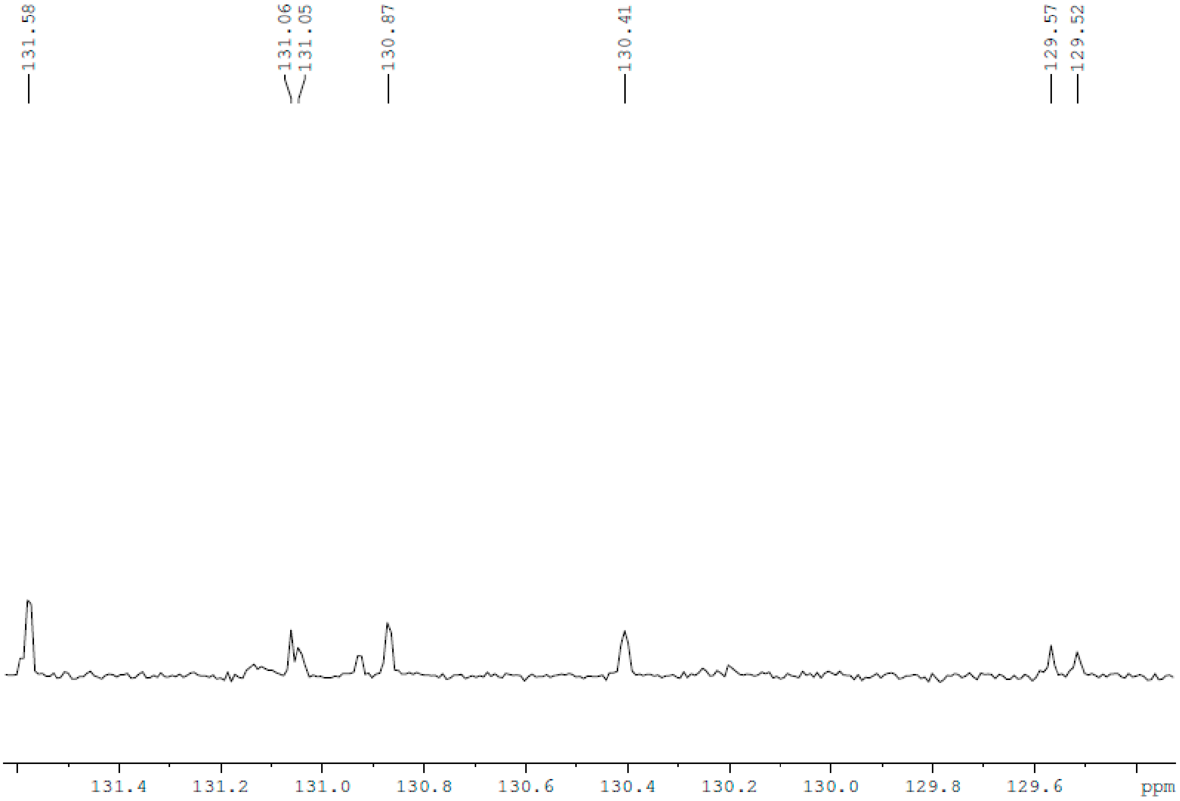
131.58
131.06
131.05
130.87
130.41
129.57
129.52
131.4
131.2
131.0
130.8
130.6
130.4
130.2
130.0
129.8
129.6
ppm

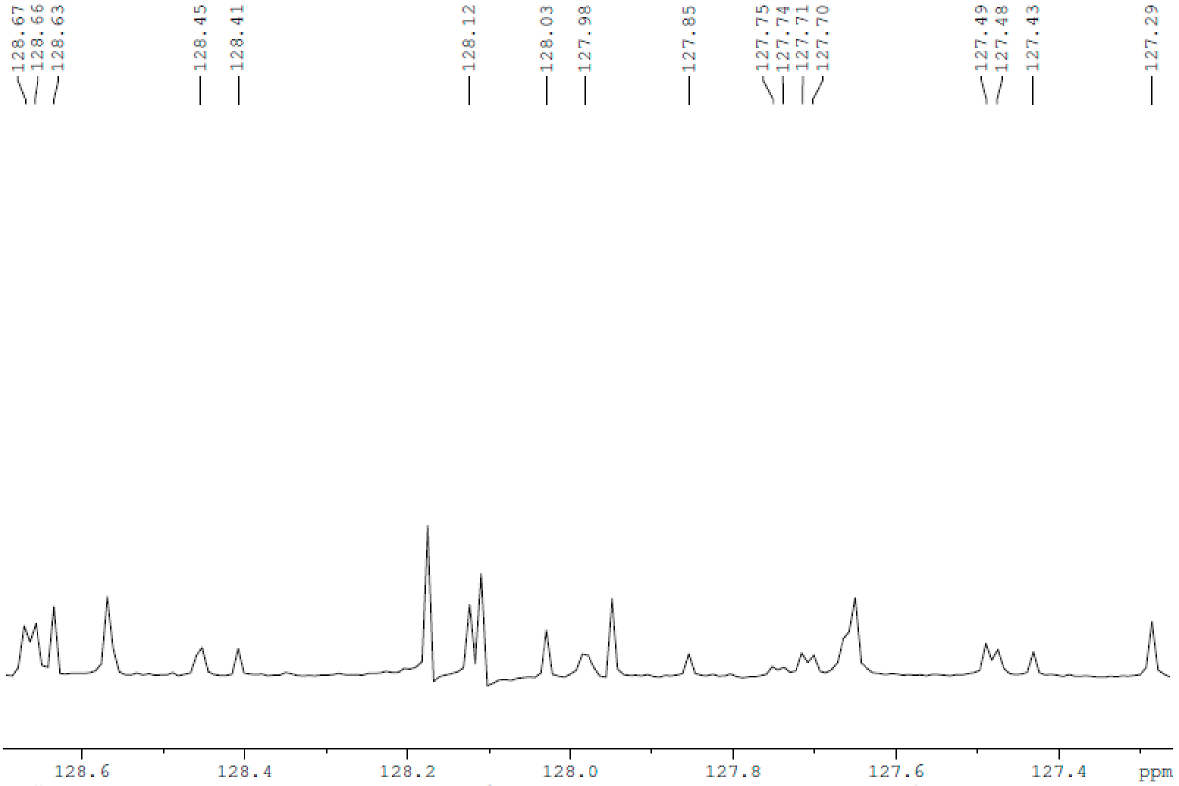
128.67
128.66
128.63
128.45
128.41
128.12
128.03
127.98
127.85
127.75
127.74
127.71
127.70
127.49
127.48
127.43
127.29
128.6
128.4
128.2
128.0
127.8
127.6
127.4
ppm

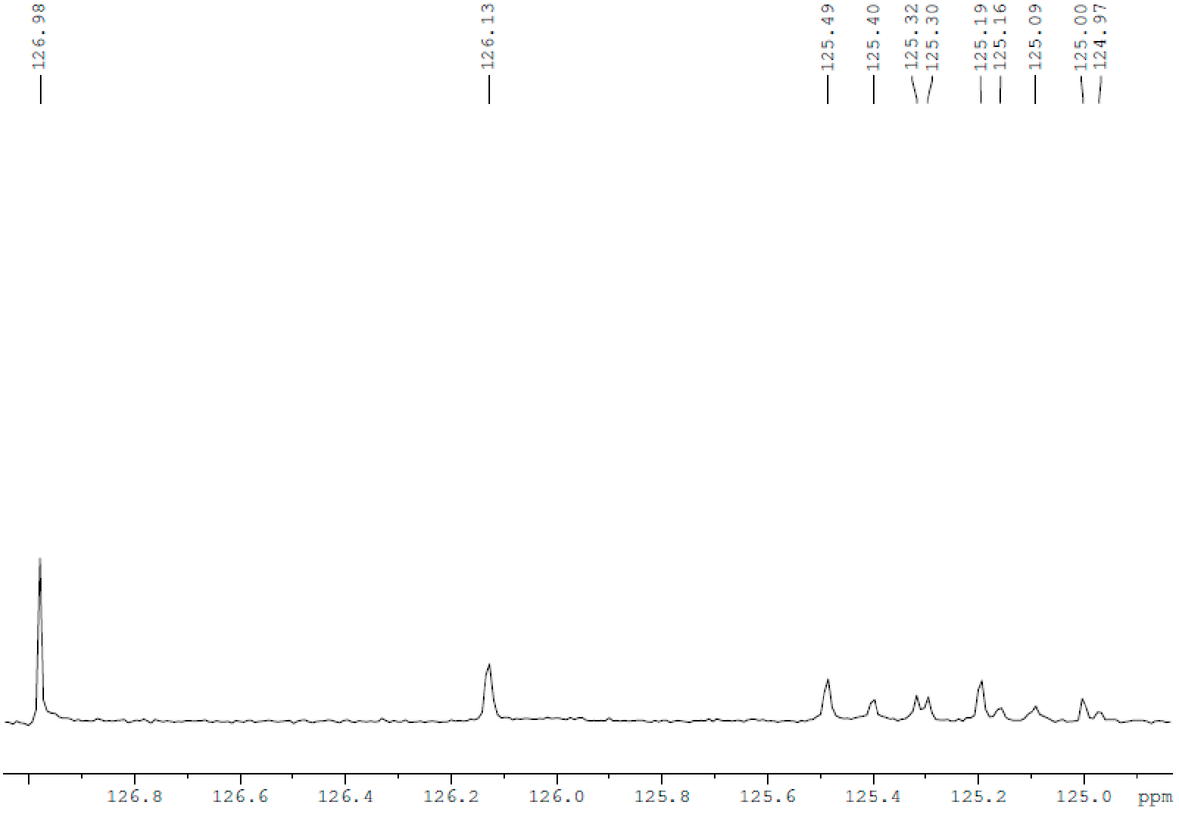
126.98
126.13
125.49
125.40
125.32
125.30
125.19
125.16
125.09
125.00
124.97
126.8
126.6
126.4
126.2
126.0
125.8
125.6
125.4
125.2
125.0
ppm

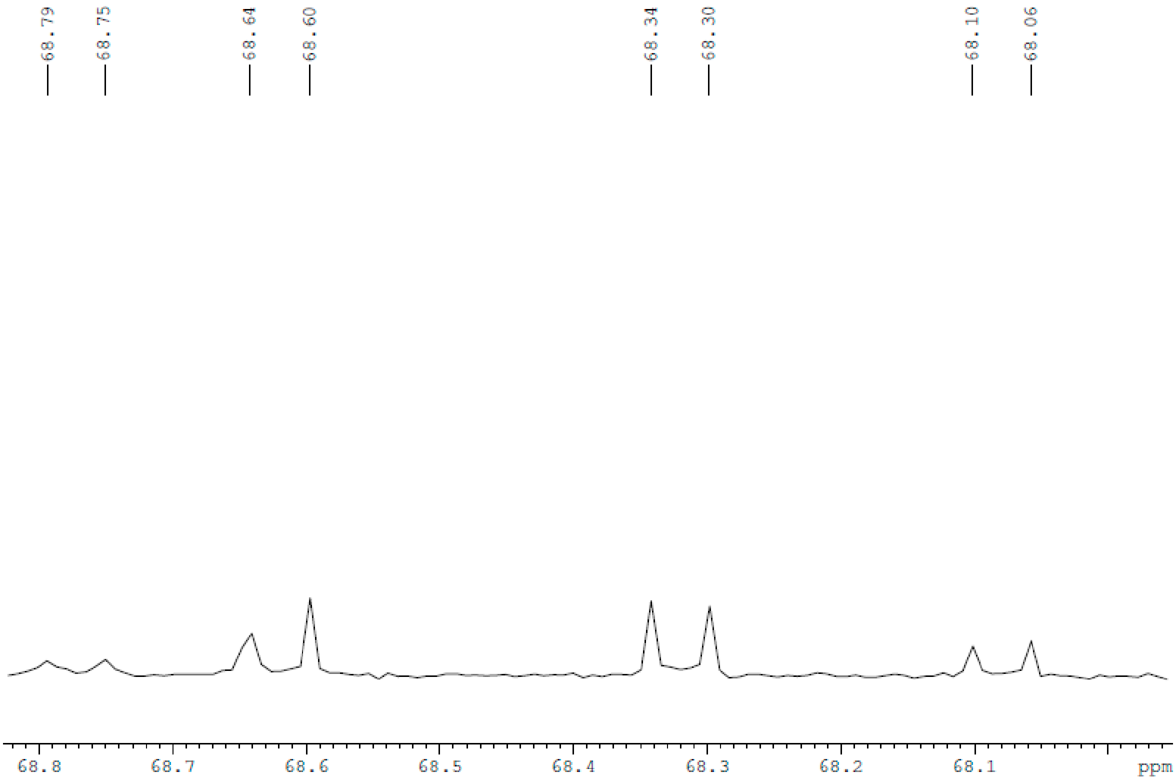
68.79
68.75
68.64
68.60
68.34
68.30
68.10
68.06
68.8
68.7
68.6
68.5
68.4
68.3
68.2
68.1
ppm

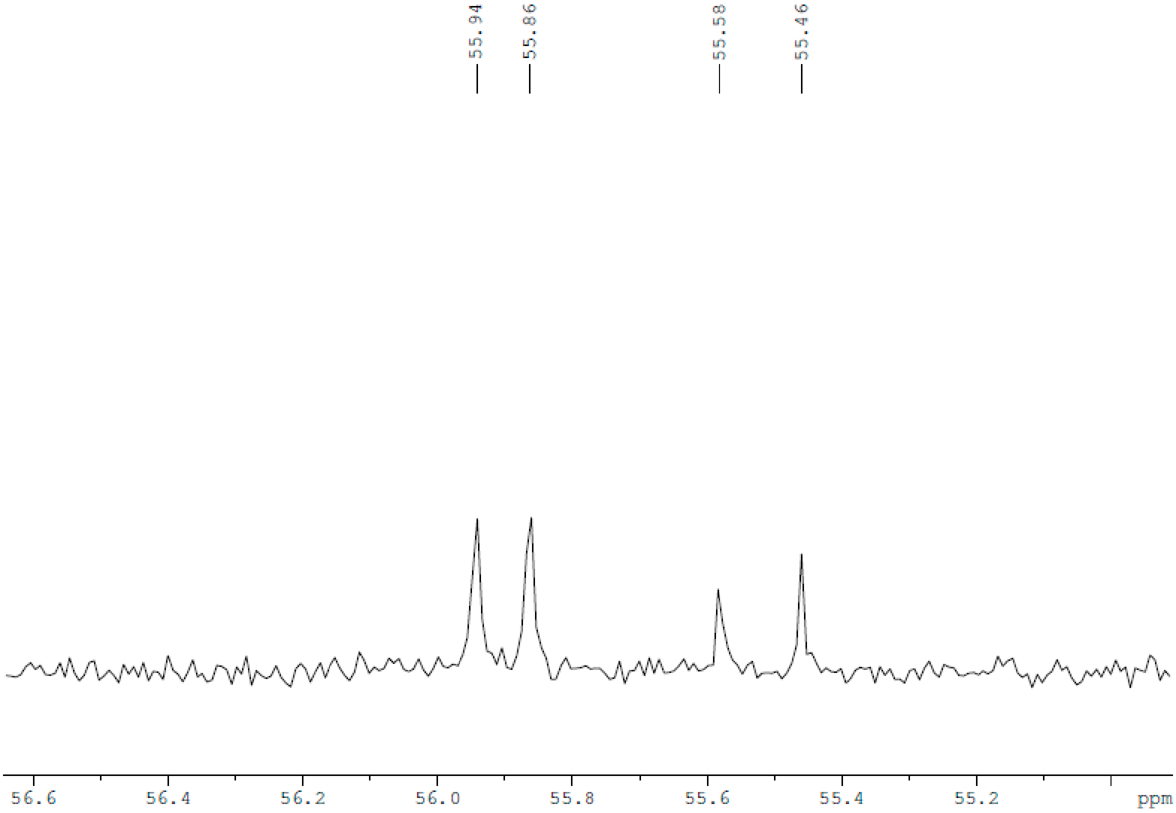
55.94
55.86
55.58
55.46
56.6
56.4
56.2
56.0
55.8
55.6
55.4
55.2
ppm

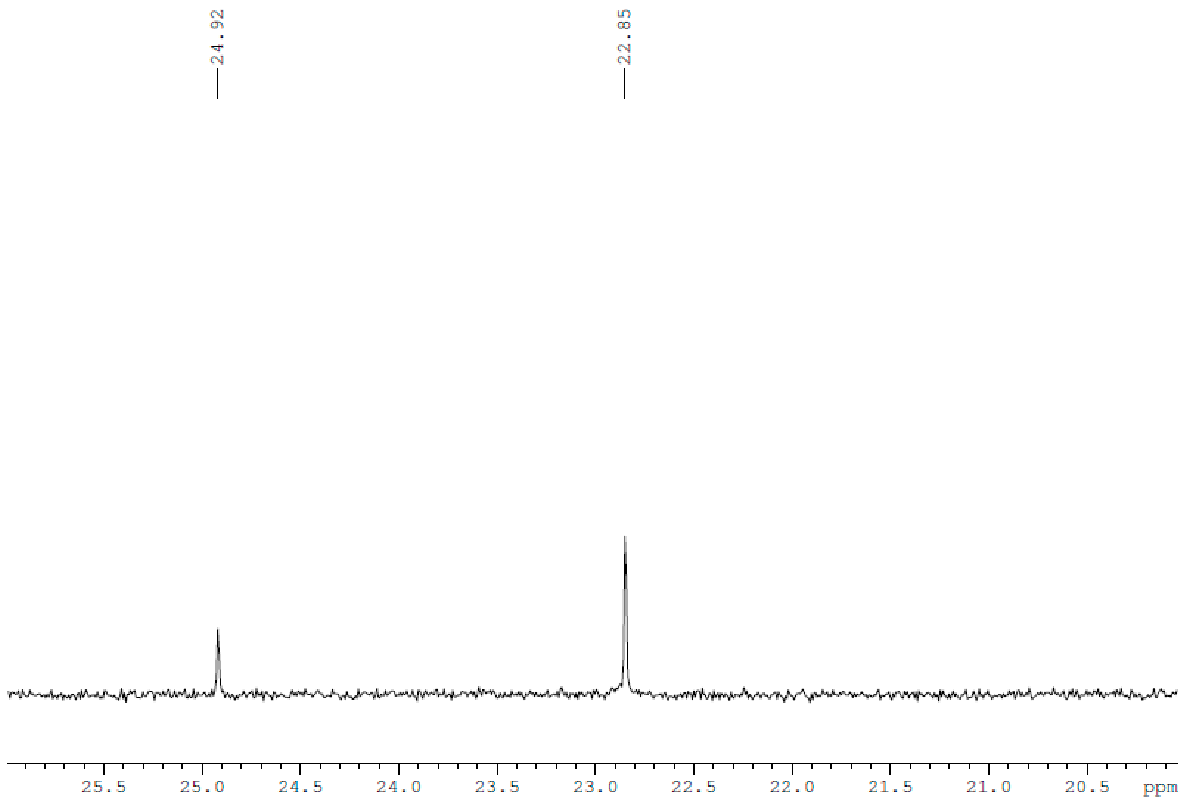

Figure S7. (**c**) Dibenzyl *N*-(*R*)-α-methylbenzylamino(pyren-1-yl)methylphosphonate (**5**). Mixture of diastereoisomers ^{13}C-NMR—followed by enlarged fragments.
